# Direct trifluoroethylation of carbonyl sulfoxonium ylides using hypervalent iodine compounds

**DOI:** 10.3762/bjoc.20.263

**Published:** 2024-12-04

**Authors:** Radell Echemendía, Carlee A Montgomery, Fabio Cuzzucoli, Antonio C B Burtoloso, Graham K Murphy

**Affiliations:** 1 São Carlos Institute of Chemistry, University of São Paulo, 13560-970, São Carlos, SP, Brazil; 2 Department of Chemistry, University of Waterloo, 200 University Ave W., Waterloo, Ontario, Canada

**Keywords:** alkylation, DFT calculations, fluorine chemistry, hypervalent iodine, sulfoxonium ylide, sulphur ylides

## Abstract

A novel study on the hypervalent iodine-mediated polyfluoroalkylation of sulfoxonium ylides was developed. Sulfoxonium ylides, known for their versatility and stability, are promising substrates for numerous transformations in synthetic chemistry. This report demonstrates the successful derivatization of sulfoxonium ylides with trifluoroethyl or tetrafluoropropyl groups, and provides valuable insights into the scope and limitations of this approach. Nineteen examples have been prepared (45–92% yields), with structural diversity modified at two key sites on the sulfoxonium ylide reactants. Finally, DFT calculations provided insights about the mechanism of this transformation, which strongly suggest that an S_N_2 reaction is operative.

## Introduction

**Figure 1:**
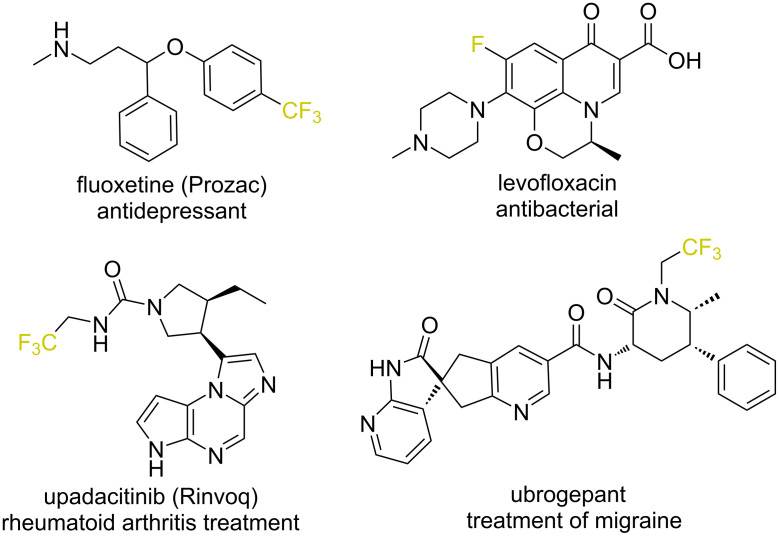
Representative examples of fluorine containing, biologically active compounds.

Introducing fluorine or fluoroalkyl motifs into organic molecules or key frameworks stands out as a crucial and appealing approach in uncovering and crafting innovative drugs, agrochemicals, and functional materials (Figure 1) [1–4]. Fluorinated functional groups can positively alter the electronic characteristics of compounds, increase their metabolic stability, and boost their lipophilicity [5–7]. Consequently, developing new synthetic techniques that incorporate fluorine and fluorinated groups represents a significant area of research in synthetic organic chemistry [8–9].

Among the various fluorine-containing functional groups, the 2,2,2-trifluoroethyl group (CF_3_CH_2_), is gaining significant interest from synthetic chemists. This is due to its reduced electron-withdrawing aspect compared to the CF_3_ group, its larger steric bulk and increased polarity. This moiety is also considered as a bioisostere of the ethyl or ethoxy groups and therefore it is very attractive for applications in medicinal chemistry and related areas [10–13].

α-Carbonyl sulfoxonium ylides are well recognized as more stable and more easily handled surrogates of diazo compounds [14–15]. They have also emerged as versatile intermediates in organic synthesis due to their unique reactivity and ability to participate in a wide range of chemical transformations. In this scenario, sulfoxonium ylides are excellent substrates for bifunctionalization reactions, due to the ambiphilic character in their ylidic carbon [16]. This synthetic potential has been demonstrated in a range of insertions into polar bonds [17–20], C−H activation transformations [21–23], and geminal difunctionalizations [24–25].

**Scheme 1:**
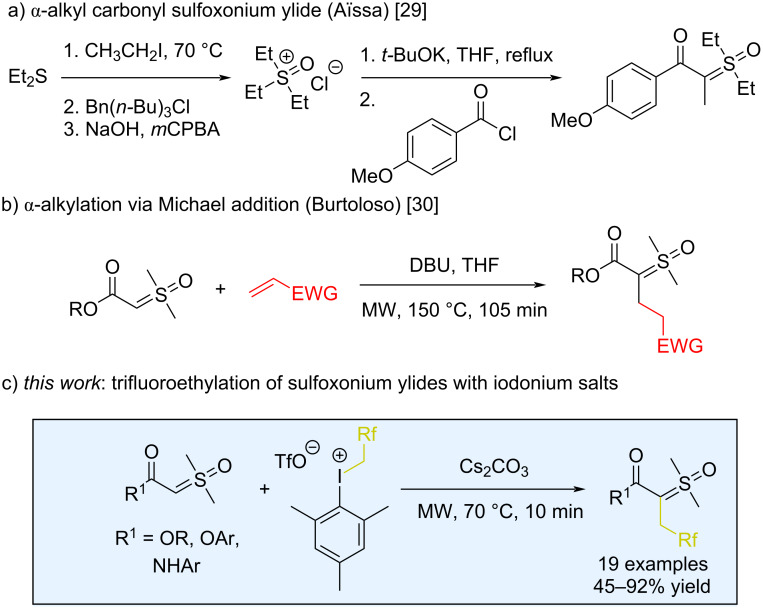
Strategies for the synthesis of α-alkyl sulfoxonium ylides.

Within the literature, a broad array of classical methods describes the synthesis of sulfoxonium ylides [26]. The most frequently used involves deprotonating the corresponding sulfoxonium salt with strong base, followed by the addition of an acylating agent (usually an acid chloride or chloroformate). Nevertheless, achieving a wide range of structural variations in sulfoxonium salts or ylides, particularly those that lead to α-alkyl-substituted compounds, is still challenging [27]. For example, in the S_N_2 reaction of alkyl halides with sulfoxonium ylides, the initially formed α-alkyl-substituted ylide reacts further with the halide to expel the sulfoxide and ultimately generate an α-halogenated product [28]. In 2017, the Aϊssa group described a procedure to better synthesize such α-alkyl-substituted carbonyl sulfoxonium ylides [29]. This protocol involved the alkylation of a dialkyl thioether, counterion exchange, oxidation, and eventual acylation (Scheme 1a). More recently, the Burtoloso group reported the α-alkylation of carbonyl sulfoxonium ylides via a Michael addition approach that occurred without any competition from cyclopropanation [30]. While this reaction represented the first direct alkylation of sulfoxonium ylides, it was nonetheless limited to the more reactive ester ylide variants (Scheme 1b). As far as we know, aside from the methodologies mentioned above, there are no other reports on the direct alkylation or fluoroalkylation of these ylide compounds.

In line with our ongoing interest in the chemistry of sulfoxonium ylides, we aimed to develop a new alkylation methodology using fluoroalkyliodonium salts as sources of electrophilic trifluoroethyl synthon. Given the non-nucleophilic nature of the iodoarene byproduct, this protocol should not suffer from further reactivity that decomposes the ylide. We describe here the coupling of α-carbonyl sulfoxonium ylides with polyfluoroalkyl(aryl) hypervalent iodonium salts, for the efficient synthesis of fluorinated sulfoxonium ylides (Scheme 1c).

## Results and Discussion

Since the introduction of hypervalent iodonium salts in organic chemistry, these valuable reagents have led to many new strategies for carbon–carbon bond formation [31–32]. Our research groups recently reported the α-arylation between sulfoxonium ylides and diaryliodonium salts [33], and encouraged by this precedent, we envisioned that the chemistry between sulfoxonium ylides and hypervalent iodine compounds might be ripe for further exploitation. The trifluoroethyliodonium salt discovered by Umemoto has proven an effective electrophilic trifluoroethyl transfer reagent [34–35], and to further explore the potential of fluoroalkyliodonium salts we evaluated the reactivity of such compounds in the context of sulfoxonium ylide derivatization.

As depicted in Table 1, we began our studies using methyl ester sulfoxonium ylide **1a** and 2,2,2-trifuoroethyl(mesityl)iodonium triflate salt (**2a**), as model substrates (see also Table S1 in Supporting Information File 1). Combining these at room temperature in acetonitrile produced **3a** in 8% ^1^H NMR yield (Table 1, entry 1). Repeating the reaction with Cs_2_CO_3_ (1.3 equiv) produced **3a** in a much improved 60% yield (Table 1, entry 2). We screened other solvents and tested the impact of other inorganic bases but none of these changes improved the formation of the ylide **3a** (Table 1, entries 3–6). And while increasing the reaction concentration was also not effective (Table 1, entry 7; 56% yield), extending the reaction time to 24 hours at room temperature gave **3a** in 69% yield (Table 1, entry 8). Other chlorinated, ethereal or polar solvents were also tested under this prolonged reaction time, but none proved better than acetonitrile (Table 1, entries 9–12). We attempted to decrease the reaction time by increasing the temperature, but these changes resulted in decreased yields of **3a** (Table 1, entries 13 and 14). Surprisingly, when using microwave (MW) heating at 70 ⁰C for 10 min in ACN, product **3a** was formed in 74% yield (Table 1, entry 15). An additional solvent screen under microwave conditions offered no improvement (Table 1, entries 16–18). Finally, multivariate screening ultimately showed that **3a** could be obtained in 79% ^1^H NMR yield (75% isolated yield, Table 1, entry 19) when using **1a** (1.0 equiv), **2a** (2.0 equiv) in ACN (1 M) with Cs_2_CO_3_ (1.0 equiv) under microwave irradiation at 70 °C for 10 min. Though the yield only improved by 5% compared with using 1.3 equiv of **2a**, these were nonetheless adopted as the optimal reaction conditions.

**Table 1:** Optimization of reaction conditions.

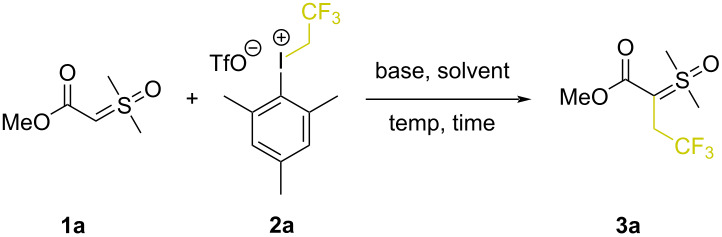					

Entry^a^	Temperature	Time	Solvent	Base	Yield **3a** (%)^b^

1	rt	6 h	ACN	–	8
2	rt	6 h	ACN	Cs_2_CO_3_	60
3	rt	6 h	dioxane	Cs_2_CO_3_	NR
4	rt	6 h	Et_2_O	Cs_2_CO_3_	22
5	rt	6 h	ACN	Na_2_CO_3_	27
6	rt	6 h	ACN	K_3_PO_4_	48
7^c^	rt	6 h	ACN	Cs_2_CO_3_	56
8	rt	24 h	ACN	Cs_2_CO_3_	69
9	rt	24 h	DCM	Cs_2_CO_3_	7
10	rt	24 h	DCE	Cs_2_CO_3_	2
11	rt	24 h	THF	Cs_2_CO_3_	NR
12	rt	24 h	AcOEt	Cs_2_CO_3_	9
13	50 °C	1 h	ACN	Cs_2_CO_3_	46
14	60 °C	30 min	ACN	Cs_2_CO_3_	49
15	70 °C (MW)	10 min	ACN	Cs_2_CO_3_	74
16	70 °C (MW)	10 min	DCE	Cs_2_CO_3_	62
17	70 °C (MW)	10 min	AcOEt	Cs_2_CO_3_	70
18	70 °C (MW)	10 min	TFE	Cs_2_CO_3_	2
**19** **^c,d^**	**70 °C (MW)**	**10 min**	**ACN**	**Cs** **_2_** **CO** **_3_**	**79 (75** **^e^** **)**

^a^Reaction performed with **1a** (0.2 mmol, 1.0 equiv), **2a** (0.26 mmol, 1.3 equiv), solvent (0.4 mL) and base (0.26 mmol, 1.3 equiv). ^b1^H NMR yield using (trifluoromethyl)benzene as internal standard. ^c^Reaction performed with 0.2 mL of solvent. ^d^Using 2.0 equiv of **2a** and 1.0 equiv Cs_2_CO_3_. ^e^Isolated yield.

**Scheme 2:**
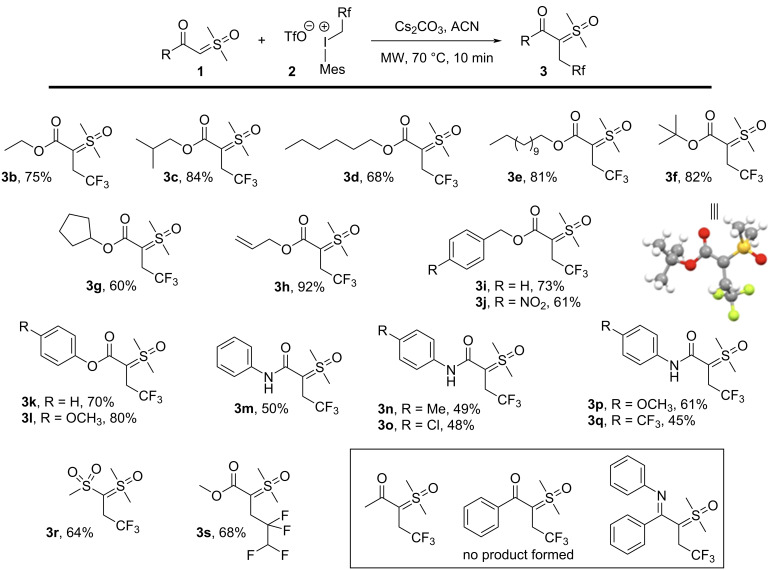
Exploring substrate scope in the direct α-fluoroalkylation of sulfoxonium ylides.

Once the optimal reaction conditions were established, we then investigated the scope and limitations of this novel transformation (Scheme 2). Initially, we investigated the effects of introducing various substituents around the ester group of the carbonyl sulfoxonium ylide. We discovered that the reaction worked very well for various alkyl ester derived substrates (**3b–g**). For instance, when the bulky *tert*-butyl ester sulfoxonium ylide was used, the fluoroalkyl product **3f** was obtained in 82% yield. A 60% yield was obtained for **3g** when the reaction was carried out with the cyclopentyl ester ylide derivative. The allyl sulfoxonium ylide reacted to produce **3h** in an excellent 92% yield, however, the related benzyl derived ylides gave **3i** and **3j** in 73% and 61% yields, respectively. Phenyl esters performed well with this methodology (**3k,l**), but switching to anilide-derived ylides were consistently poorer performing. The *N*-phenyl ylide derivative reacted to produce **3m** in only 50% yield, and comparable yields were observed for the *p*-tolyl and *p*-chlorophenyl derivatives **3n** and **3o**. A slight increase in yield was found with the *p*-anisyl derivative (**3p**, 61% yield), whereas the yield decreased when the arene was appended with an electron-withdrawing CF_3_ group (**3q**, 45% yield). Though we were unable to isolate any N-alkylated products, it is possible that competing C-alkylation and N-alkylation processes were responsible for the decreased yields observed with the anilides (compared to the ester-derived precursors). Finally, the bis-sulfonyl ylide reacted to produce **3r** in good yield (64%), and the methyl ester-derived sulfoxonium ylide could be reacted with a tetrafluoropropyl(mesityl)iodonium salt to produce tetrafluoropropyl ylide **3s** in 68% yield. These results show that a wide range of sulfoxonium ylides can be efficiently transformed to their corresponding polyfluoroalkylated derivatives in moderate to very good (45–92%) yields. It is nonetheless crucial to underscore that the reaction developed herein was ineffective with aromatic and aliphatic variants of both keto and imino sulfoxonium ylides, and no reaction was observed in any of the attempts (see Supporting Information File 1 for details). This limitation is attributed to the diminished reactivity of these ylides compared to ester and amide-derived sulfoxonium ylides.

**Scheme 3:**
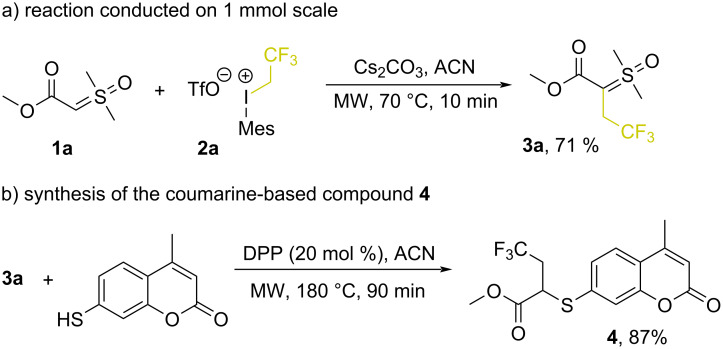
Synthetic applications of fluoroalkylated sulfoxonium ylides.

Having established this new methodology, we then turned our attention to demonstrate additional synthetic applications of this procedure. The reaction could be easily performed on a 1 mmol scale, which gave the desired product **3a** in 71% yield (Scheme 3a). Lastly, product **3a** was subjected to our previously developed S–H insertion reaction protocol with sulfoxonium ylides [36], which generated a new 2,2,2-trifluoroethylcoumarine-based compound **4** in 87% yield (Scheme 3b).

**Figure 2:**
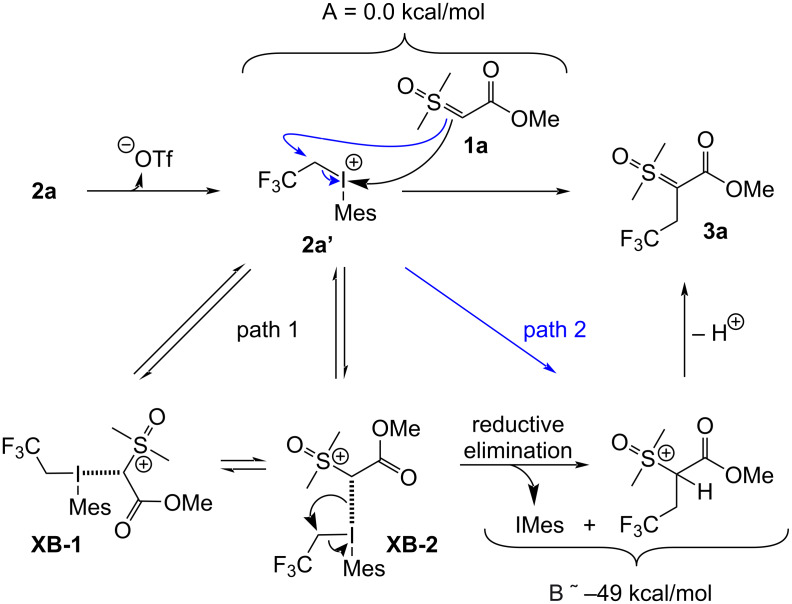
Possible mechanisms for the reaction of **1a** and **2a** leading to **3a** (via **B**), proceeding via either halogen-bonded adducts and reductive elimination (path 1) or directly via an S_N_2 reaction (path 2).

To gain insight into the mechanism, we modelled two reaction pathways commonly suggested for the 2,2,2-trifuoroethyl(mesityl)iodonium triflate salt, and for related diaryliodonium salts (Figure 2). The first mechanism explored was the associative pathway that terminates via reductive elimination (path 1) [37–39]. This pathway initiates by formation of a halogen bond complex between **1a** and the trifuoroethyl(mesityl)iodonium ion **2a’**, where adduct **XB-1** is presumably in equilibrium with isomeric **XB-2**. Reductive elimination of the iodoarene from **XB-2** would furnish **B**, whose deprotonation would complete the pathway. The alternative mechanistic proposal is an S_N_2 reaction [40–42] between **1a** and the iodonium ion **2a’**, which directly furnishes **B** without invoking halogen bonded adducts.

**Figure 3:**
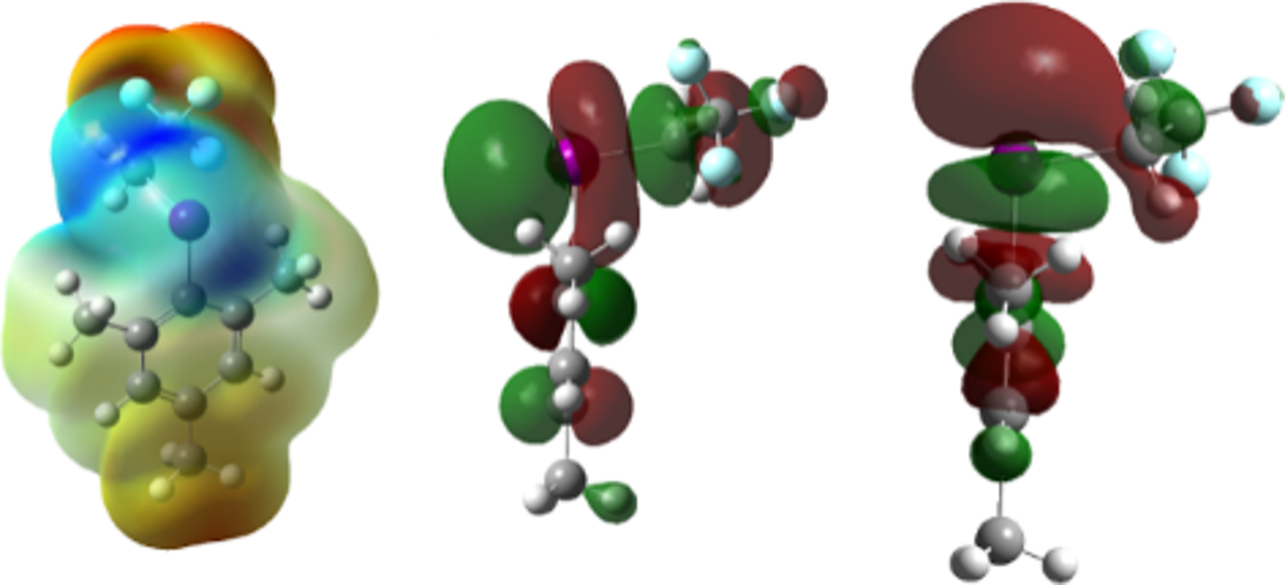
Electrostatic potential of **2a’** from 0.075 *e* to 0.21 *e*, showing two sigma holes of potentials 0.20 and 0.21 *e* (left). LUMO (middle) and LUMO+1 (right) of **2a’**.

To assess which of these mechanistic possibilities was more probable, we turned to computational analysis. The geometries of starting materials **1a** and **2a’** were pre-optimized in the solvated phase using Gaussian 16 at the PBE0/def2-TZVP/def2-TZVPD level of theory, after a conformation search using Crest [43–49]. Next, the electrostatic potential map of the iodonium ion **2a’** was generated using an isodensity surface of 0.001 a.u. [50]. This showed two sigma holes of different potentials, with the stronger (0.21 *e*) residing opposite the arene, and the slightly weaker (0.20 *e*) hole residing opposite the trifluoroethyl motif (Figure 3, left). These sigma-hole potentials are consistent with them being viable electrophilic sites for halogen bond formation with **1a**, supporting the mechanism in path 1. We also expressed the LUMO and LUMO+1 molecular orbitals of **2a’** (Figure 3, middle and right, respectively), which showed lobes centered on the I–C bonds to both the trifluoroethyl and arene moieties. These observations confirmed the LUMO as an appropriate lobe for nucleophilic attack via the S_N_2 pathway (path 2), and confirmed the LUMO+1 as an appropriate lobe for substitution via reductive elimination (path 1). As such, neither mechanism could be immediately discarded, and we were encouraged to further explore these computationally.

For both pathways, a potential energy surface (PES) was used to generate an ‘initial guess’ for stationary and saddle point geometries (see also section 4.1 in Supporting Information File 1). The PES scan strongly indicated that the S_N_2 mechanistic proposal was operative, owing to its lower-energy barrier; however, the saddle point geometries identified at this low level of theory were not close enough to the true transition state geometries to meet convergence criteria. Thus, using the guidance of the PES scans, **A**, **XB-2**, and **B** were subjected to the nudged elastic band climbing image (NEB-CI) method using Orca 5.0.1 at the PBE/def2-SVP D4 level of theory in the CPCM solvation model (see also section 4.2 in Supporting Information File 1) [51–56].

Both climbing images were subjected to transition state optimization and successfully met convergence criteria. Consistent with the PES scan outcome, the NEB-CI approach also indicated that the S_N_2 mechanistic proposal (Figure 2, path 2) dominates.

**Figure 4:**
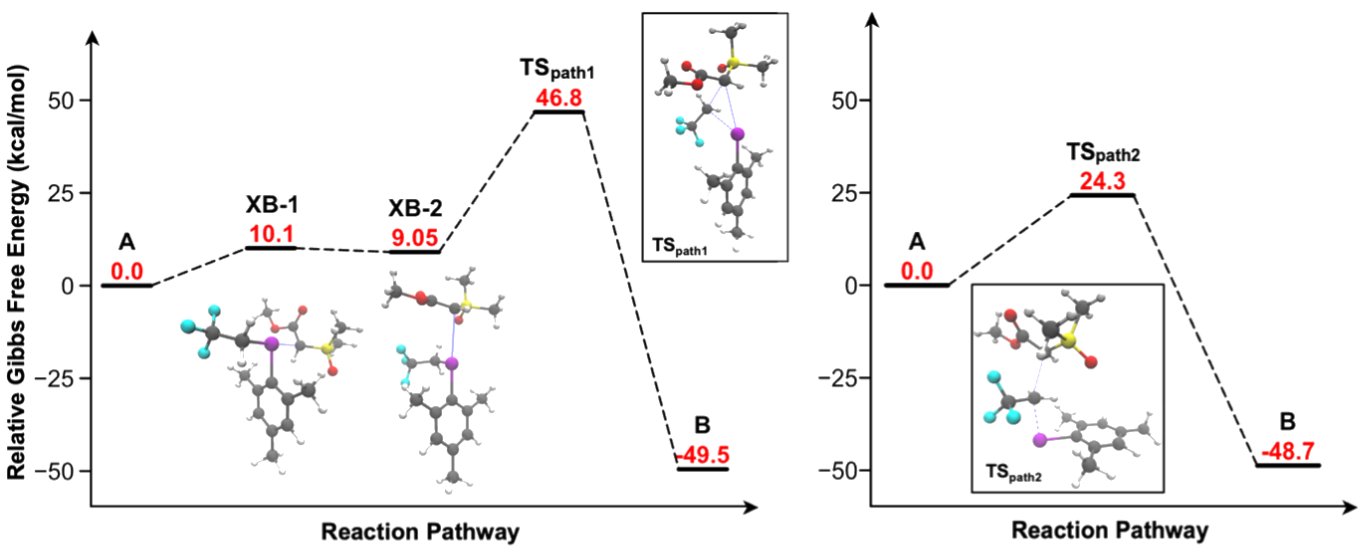
The optimized reaction coordinate diagrams for the halogen bond-mediated mechanism (path 1, left) and the S_N_2 mechanism (path 2, right).

Finally, for both mechanistic pathways, all intermediates and transition states were subjected to optimization and frequency calculations using Orca 5.0.1 at the PBE0/def2-TZVPD [57] level of theory, to generate reaction coordinate diagrams for both pathways (Figure 4). Both barrier steps were confirmed using the intrinsic reaction coordinate (IRC) method. Ion **2a’** was found to have an initial bond length of 2.1 Å. The transition to **XB-1** was found at a relative energy of 10.1 kcal/mol, where the C–I–C bond angle is 178° with C–I bond lengths of 2.2 Å (I–CH_2_CF_3_) and 2.9 Å. The reaction coordinate diagram for path 1 showed a near barrierless equilibrium between halogen bond adducts **XB-1** and **XB-2**, where **XB-2** has a C–I–C bond angle of 86° and C–I bond lengths of 2.1 Å (I–CH_2_CF_3_) and 3.2 Å. Finally, a 37.8 kcal/mol activation energy between **XB-2** and **B** for path 1 was calculated. On the other hand, path 2 had a much lower Gibbs free energy of activation of 24.3 kcal/mol, where the angle of attack from **1a** to **2a’** was found at approximately 160° with equal C–I bond lengths of 2.5 Å in the transition state. The significantly lower activation energy allowed us to conclude that the S_N_2 mechanism was the more favourable pathway.

## Conclusion

In conclusion, we have developed a direct polyfluoroalkylation reaction of sulfoxonium ylides. The easily available 2,2,2-trifluoroethyl(mesityl)iodonium triflate reagent enabled the straightforward trifluoroethylation of diverse sulfoxonium ylides under mild conditions and short reaction times. Various computational strategies were also employed to differentiate between competing halogen bond-mediated and S_N_2 reaction mechanisms. Ultimately, the nudged elastic band climbing image (NEB-CI) method predicted the S_N_2 pathway to be favoured, and transition state optimization showed this to possess a Gibbs free energy of activation of 24.3 kcal/mol. This report shows the ease with which sulfoxonium ylides can be derivatized using hypervalent iodine reagents, and our continued efforts towards this will be reported in due course.

## Experimental

### Representative procedure for 2,2,2-trifluoroethylation of sulfoxonium ylides

An oven dried 5 mL microwave flask containing a magnetic stirrer was charged with Cs_2_CO_3_ (70.5 mg, 0.2 mmol, 1.0 equiv), sulfoxonium ylide (0.2 mmol, 1.0 equiv) and the corresponding fluoroethyliodonium salt (0.40 mmol, 2.0 equiv). The reaction vessel was capped with a rubber septum and filled with nitrogen. Then ACN (0.2 mL) was added. The rubber septum was removed and the microwave vial was quickly capped with a Teflon microwave cap. The reaction was heated to 70 ºC for 10 min. The crude mixture was dissolved with DCM (3 mL), the solvent was removed under reduced pressure to furnish a crude product that was purified by flash column chromatography, using silica gel 60 (200–400 mesh) as a stationary phase (eluent *n*-hex/AcOEt 5:95%).

## Supporting Information

File 1 Experimental part, NMR spectra, computational details and crystallgraphic data.

## Data Availability

All data that supports the findings of this study is available in the published article and/or the supporting information to this article.
