# Supplementary material for: Direct trifluoroethylation of carbonyl sulfoxonium ylides using hypervalent iodine compounds
Source: Beilstein J Org Chem. 2024 Dec 4;20:3182–90. doi: 10.3762/bjoc.20.263 (PMC11635294; doi:10.3762/bjoc.20.263)
Supplement: File 1 — Experimental part, NMR spectra, computational details and crystallgraphic data. [file Beilstein_J_Org_Chem-20-3182-s001.pdf]

BEILSTEIN JOURNAL OF ORGANIC CHEMISTRY

Supporting Information

for

Direct trifluoroethylation of carbonyl sulfoxonium ylides using hypervalent iodine compounds

Radell Echemendía, Carlee A. Montgomery, Fabio Cuzzucoli, Antonio C. B. Burtoloso and Graham K. Murphy

Beilstein J. Org. Chem. **2024,** *20,* 3182–3190. doi:10.3762/bjoc.20.263

Experimental part, NMR spectra, computational details and crystallgraphic data

License and Terms: This is a supporting information file under the terms of the Creative Commons Attribution License (https://creativecommons.org/licenses/by/4.0). Please note that the reuse, redistribution and reproduction in particular requires that the author(s) and source are credited and that individual graphics may be subject to special legal provisions.
The license is subject to the *Beilstein Journal of Organic Chemistry* terms and conditions: (https://www.beilstein-journals.org/bjoc/terms)

TABLE OF CONTENTS

1. General information S2

2. General procedures and experimental section. S3

General procedure A: preparation of sulfoxonium ylides. S3

General procedure B: synthesis of mesityl(2,2,2-trifluoroethyl)iodonium salts S9

Table S1. Optimization of the reaction conditions. S11

Frequently asked questions (FAQ's) S13

3. NMR spectra. S26

4. Computational general details S66

4.1 PES scan results S68

4.2 NEB results S70

4.3 Cartesian coordinates of fully optimized structures S72

5. X-ray crystallography data for compound **3f.** S77

1. General information

Reaction setup: Air- and moisture-sensitive reactions were conducted in flame- or oven-dried glassware equipped with tightly fitted rubber septa and under a positive pressure of ultra-purified argon (>99.999%) or nitrogen. Unless stated otherwise, all the yields refer to isolated products after flash column chromatography.

NMR Spectroscopy: Proton nuclear magnetic resonance (^{1}H NMR) spectra were recorded using 300 MHz Bruker Ascend instrument and a 600 MHz Bruker instrument and 400 MHz Agilent Technologies, 400/54 Premium Shielded. For ^{1}H NMR spectra, chemical shifts are referenced from TMS (0.00 ppm). Coupling constants (*J*) are reported in Hz. Carbon nuclear magnetic resonance (^{13}C NMR) spectra were recorded using an NMR spectrometer at 75 MHz and 151 MHz. For ^{13}C NMR spectra, chemical shifts are given from $CDCl_3$ (77.16 ppm). ^{1}H NMR multiplicities are reported as follows: s = singlet; brs= broad singlet, d = doublet; t = triplet; q = quartet; m = multiplet.

Infrared spectroscopy: InfraRed (IR) data was recorded using an ATR-FTIR (Attenuated Total Reflection Fourier Transform InfraRed) instrument.

Mass spectrometry: High-resolution mass spectroscopy was performed on a Thermo Fisher Scientific Q-Exactive hybrid mass spectrometer and with a UPLC H-class liquid chromatograph connected to a Waters Xevo G2-XS QToF mass spectrometer via an electrospray ionization (ESI) interface. Accurate mass determinations were performed at a mass resolution of 70,000 and recorded using positive ion electrospray ionization. Samples were infused at 10 µL/min in 1:1 CH_3CN/H_2O + 0.1% formic acid.

Melting points: All melting points were measured in a Mel-Temp II Capillary Melting Point apparatus and are uncorrected.

Solvents/chemicals: Ethyl acetate, hexanes, methanol, dioxane and chloroform were used as received. Acetonitrile, was stirred with flame dried K_2CO_3 overnight before sequential drying with 4 Å molecular sieves (2×), $CHCl_3$ and methyl *tert*-butyl ether were dried over 4 Å molecular sieves prior to use (2×). Other solvents were dried and purified using a JC Meyer solvent purification system and were used without any further purification. All other reagents were used as received from the manufacturer.

X-ray: The single-crystal X-ray diffraction studies were performed by using a Bruker Kappa Apex II CCD equipped with Mo−Kα radiation at room and low temperature. X-ray crystal

structure was determined by Dr. Jalil Assoud at the University of Waterloo and figures of X-ray crystal structure was generated by using Mercury.

Microwave synthesis: Microwave experiments were performed in two different microwave synthesis reactors, AntonPaar Monowave 300 and Biotage-Initiator Robot Eight, using sealed glass vials (0.5–2.0 mL) equipped with a snap cap and a silicon septum.

2. General procedures and experimental section.

General procedure A: preparation of sulfoxonium ylides.

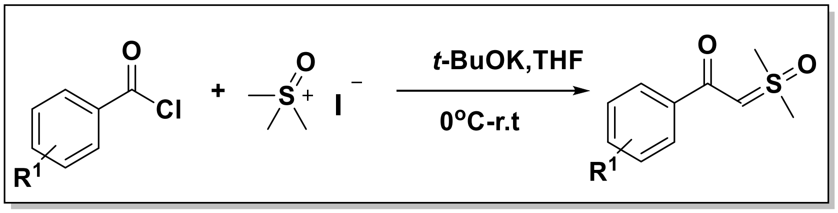

A 125 mL oven-dried round-bottom flask was attached to a reflux condenser, under an argon atmosphere, to which 1.59 g of potassium *tert*-butoxide (14.2 mmol, 2.1 equiv), 1.65 g of trimethylsulfoxonium iodide (7.5 mmol, 1.1 equiv), and 20 mL of anhydrous THF were added. The obtained suspension was heated at reflux for 30 min. After this time, the mixture was cooled at 0 °C, followed by the slow addition of a 1 mol L^{-1} solution of the appropriate acyl chloride, carbonate, chloroformate, or isocyanate (6.8 mmol, 1.0 equiv) in THF. Afterward, the cooling bath was removed, allowing the reaction mixture to naturally reach room temperature. The mixture was then stirred for an additional 30 minutes. Next, the solvent was removed on a rotary evaporator, 70 mL of water was added and the product extracted with a 3:1 CH_2Cl_2:iPrOH mixture (8 × 20 mL). The organic phase was washed with water (3 × 10 mL), dried over Mg_2SO_4, and the solvent was removed on a rotary evaporator. The crude material was purified by solubilization in the minimal amount of hot AcOEt (10–15 mL), followed by slow addition of 15 mL of hexanes. The solid was filtered and washed with two portions of a 2:1 mixture of hexanes: AcOEt (2 × 10 mL), furnishing the respective ketosulfoxonium ylide. It is worth to mention that some of the compounds were also purified by column chromatography using mixtures of 3–10% MeOH/CH_2Cl_2 or MeOH/$CHCl_3$).[1]

[1] Souza, J.H.; Vargas, J. M.; Burtoloso, A.C.B. *Synthesis,* 2023; DOI: 10.1055/a-2222-3695.

Methyl 2-(dimethyl(oxo)-λ^6-sulfaneylidene)acetate (1a)

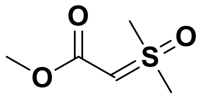

Prepared according to general procedure A from methyl chloroformate (526µL, 6.8 mmol). The spectroscopy data were in good agreement with the literature.[2] White solid (480 mg, 47%) *Rf*= 0.33 (9:1 $CHCl_3$:MeOH). **^{1}H NMR** (300 MHz, $CDCl_3$) δ = 3.93 (s, 1H), 3.61 (s, 3H), 3.37 (s, 6H) ppm.

Ethyl 2-(dimethyl(oxo)-λ^6-sulfaneylidene)acetate (1b)

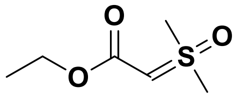

Prepared according to general procedure A from ethyl chloroformate (647µL, 6.8 mmol). The spectroscopy data were in good agreement with the literature.[3] Pale yellow oil (669 mg, 60%) *Rf*= 0.29 (9:1 $CHCl_3$:MeOH). **^{1}H NMR** (300 MHz, $CDCl_3$) δ = 4.12-4.05 (m, 2H), 3.95 (s, 1H), 3.38 (s, 6H), 1.23 (t, *J* = 7.1 Hz, 3H) ppm.

Isobutyl 2-(dimethyl(oxo)-l6-sulfaneylidene)acetate (1c)

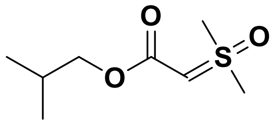

Prepared according to general procedure A from isobutyl chloroformate (883 µL, 6.8 mmol). White solid (914 mg, 70%) *Rf*= 0.34 (9:1 $CHCl_3$:MeOH). The spectroscopy data were in good agreement with the literature.[4] **^{1}H NMR** (300 MHz, $CDCl_3$) δ = 3.92 (s, 1H), 3.76 (d, *J* = 6.7 Hz, 2H), 3.34 (s, 6H), 1.87 (hept, *J* = 6.6 Hz, 1H), 0.93 (d, *J* = 6.7 Hz, 6H) ppm.

Hexyl 2-(dimethyl(oxo)-λ^6-sulfaneylidene)acetate (1d)

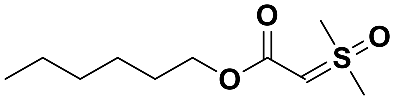

Prepared according to general procedure A from hexyl chloroformate (1.1 mL, 6.8 mmol). Yellow solid (973 mg, 65%) *Rf*= 0.34 (10:1 $CHCl_3$:MeOH). mp=62±2 ºC. **^{1}H NMR** (300 MHz, $CDCl_3$) δ = 3.98 (t, *J* = 6.7 Hz, 2H), 3.91 (s, 1H), 3.35 (s, 6H), 1.65 – 1.49 (m, 2H), 1.37 – 1.19 (m, 6H), 0.84 (t, *J* = 6.9 Hz, 3H) ppm. **^{13}C NMR** (76 MHz, $CDCl_3$) δ 167.5, 63.0, 55.3, 42.4, 31.6, 29.2, 25.8, 22.6, 14.1 ppm. **IR (neat):** ν (cm^{-1}) = 3089 3010 2952 2927 2857

[2] Talero, A.G.; Martins, B. S.; Burtoloso, A. C.B.; *Org. Lett.* **2018**, 20, 22, 7206–7211.
[3] Furniel, L. G.; Echemendía, R.; Burtoloso, A. C. B. *Chem. Sci*., **2021**, *12*, 7453-7459.
[4] G. D. Bisag, S. Ruggieri, M. F and L. Bernardi, *Adv. Synth. Catal.*, **2021**, *363*, 3053-3059.

1634 1614 1334 1129 1024 872 761; **HRMS** (ESI): calcd for $C_{10}H_{21}O_3S$ [M+H+]: 221.1205; found: 221.1205.

Dodecyl 2-(dimethyl(oxo)-λ⁶-sulfaneylidene)acetate (1e)

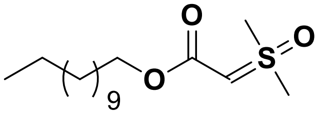

Prepared according to general procedure A from lauryl chloroformate (1.78 mL, 6.8 mmol). The spectroscopy data were in good agreement with the literature.[5] White solid (1.45 g, 70%) *Rf*= 0.31 (9:1 $CHCl_3$:MeOH); mp=79±2 ºC. **^{1}H NMR** (300 MHz, $CDCl_3$) δ = 4.01 (t, *J* = 6.8 Hz, 2H), 3.92 (s, 1H), 3.37 (s, 6H), 1.64-1.55 (m, 2H),1.35-1.24 (m, 18H), 0.87 (t, *J* = 6.9 Hz, 3H).

***tert*-Butyl 2-(dimethyl(oxo)-λ⁶-sulfaneylidene)acetate (1f)**

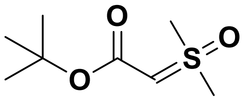

Prepared according to general procedure A from di-*tert*-butyl decarbonate (1.56 mL, 6.8 mmol). The spectroscopy data were in good agreement with the literature.[2] White solid (771 mg, 59%) *Rf*= 0.31 (9:1 $CHCl_3$:MeOH). **^{1}H NMR** (300 MHz, $CDCl_3$) δ = 3.84 (s, 1H), 3.35 (s, 6H), 1.46 (s, 9H) ppm.

Cyclopentyl 2-(dimethyl(oxo)-λ⁶-sulfaneylidene)acetate (1g)

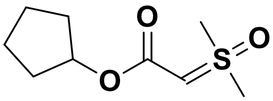

Prepared according to general procedure A from cyclopentyl chloroformate (849 µL, 6.8 mmol). White solid (1125 mg, 81%) *Rf*= 0.35 (10:1 $CHCl_3$:MeOH). mp=88±2 ºC. **^{1}H NMR** (300 MHz, $CDCl_3$) δ = 5.12 (s, 1H), 3.88 (s, 1H), 3.35 (s, 6H), 1.86-1.17 (m, 2H), 1.68-1.63 (m, 4H), 1.56-1.50 (m, 2H) ppm. **^{13}C NMR** (76 MHz, $CDCl_3$) δ 165.7, 140.4, 128.2, 123.8, 119.2, 59.4, 41.3 ppm. HRMS (ESI): calcd for $C_9H_{17}O_3S$ [M+H+]: 205.0892; found: 205.0892.

Allyl 2-(dimethyl(oxo)-λ⁶-sulfaneylidene)acetate (1h)

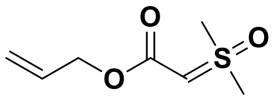

Prepared according to general procedure A from ethyl chloroformate (722 µL, 6.8 mmol). The spectroscopy data were in good agreement with the literature.[6] Pale yellow oil (1.2 g, 68 %) **^{1}H NMR** (400 MHz, $CDCl_3$) δ = 5.96 – 5.86 (m, 1H), 5.29 – 5.21 (m, 1H), 5.17 – 5.14 (m, 1H), 4.52 (d, *J* = 5.5 Hz, 2H), 3.97 (br, 1H), 3.36 (s, 6H) ppm.

[5] Echemendía, R.; To, A.; Murphy, G.K.; Burtoloso, A.C.B.; *Adv. Synth.Catal*. **2024**, *366*, 396-401.
[6] Guo, W.; Wang, M.; Han, Z.; Huang, H.; Sun, J.; *Chem. Sci*., **2021**, *12*, 11191-11196.

Benzyl 2-(dimethyl(oxo)-λ^6-sulfaneylidene)acetate (1i)

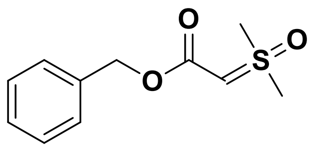

Prepared according to general procedure A from benzyl chloroformate (1.1 mL, 6.8 mmol). The spectroscopy data were in good agreement with the literature.[7] Pale yellow solid (769 mg, 50%) *Rf*= 0.29 (9:1 $CHCl_3$:CH_3OH). **^{1}H NMR** (300 MHz, $CDCl_3$) δ = 7.36 -7.27 (m, 5H), 5.08 (s, 2H), 4.02 (s, 1H), 3.32 (s, 6H) ppm.

4-Nitrobenzyl 2-(dimethyl(oxo)-λ^6-sulfaneylidene)acetate (1j)

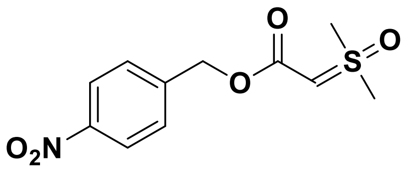

Prepared according to general procedure A from 4-nitrobenzylchlorofomate (1.45 g, 6.8 mmol). Yellow solid (959 mg, 52%) *Rf*= 0.31 (10:1 $CHCl_3$:MeOH). mp=128±2 ºC. **^{1}H NMR** (300 MHz, $CDCl_3$) δ = 8.19 (d, *J* = 8.7 Hz, 2H), 7.50 (d, *J* = 8.5 Hz, 2H), 5.17 (s, 2H), 4.08 (s, 1H), 3.40 (s, 6H) ppm. **^{13}C NMR** (76 MHz, $CDCl_3$) δ 166.7, 147.4, 145.3, 128.0, 123.8, 63.1, 55.5, 42.4 ppm. **IR (neat)**: ν (cm^{-1}) = 3089, 3017, 2930, 1634, 1602, 1519, 1379, 1331, 1135, 1134, 737; **HRMS (ESI)**: calcd for $C_{11}H_{14}O_5NS$ [M+H+]: 272.0587; found: 272.0584.

Phenyl 2-(dimethyl(oxo)-λ^6-sulfaneylidene)acetate (1k)

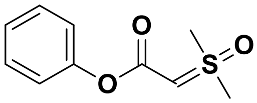

Prepared according to general procedure A from phenyl chloroformate (853 µL, 6.8 mmol). The spectroscopy data were in good agreement with the literature.[3] White solid (880 mg, 61%) *Rf*= 0.32 (9:1 $CHCl_3$: MeOH). **^{1}H NMR** (300 MHz, $CDCl_3$) δ 7.37 - 7.30 (m, 2H), 7.18 - 7.09 (m, 3H), 4.15 (s,1H), 3.41 (s, 6H) ppm.

4-Methoxyphenyl 2-(dimethyl(oxo)-λ^6-sulfaneylidene)acetate (1l)

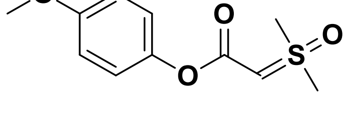

Prepared according to general procedure A from 4-methoxyphenyl chloroformate (1.01 mL, 6.8 mmol). The spectroscopy data were in good agreement with the literature.

[7] Furniel, L. G.; Burtoloso, A.C.B.; *Tetrahedron.*, **2020**, *76* ,131313.

[8]White solid (889 mg, 54%) *Rf*= 0.33 (9:1 $CHCl_3$:MeOH. **^{1}H NMR** (300 MHz, DMSO-d_6) δ = 8.54 (s, 1H), 7.40 -7.31 (m, 2H), 6.80 - 6.72 (m, 2H), 4.04 (s, 1H), 3.68 (s, 3H), 3.42 (s, 6H) ppm.

2-(Dimethyl(oxo)-λ^6-sulfaneylidene)-*N*-phenylacetamide (1m)

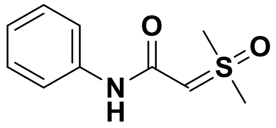

Prepared according to general procedure A from phenyl isocyanate (739 µL, 6.8 mmol). The spectroscopy data were in good agreement with the literature.[2] White solid (804 mg, 56%) *Rf*= 0.05 (9:1 $CHCl_3$:MeOH). **^{1}H NMR** (300 MHz, DMSO-d_6) δ = 8.54 (s, 1H), 7.40 -7.31 (m, 2H), 6.80 - 6.72 (m, 2H), 4.04 (s, 1H), 3.68 (s, 3H), 3.42 (s, 6H) ppm.

2-(Dimethyl(oxo)-λ^6-sulfaneylidene)-*N*-(*p*-tolyl)acetamide (1n)

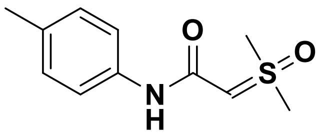

Prepared according to general procedure A from *p*-tolyl isocyanate (857 µL, 6.8 mmol). White solid (674 mg, 44%) *Rf*= 0.11 (10:1 $CHCl_3$:MeOH). mp=172±2 ºC. **^{1}H NMR** (300 MHz, DMSO-d_6) δ = 8.59 (s, 1H), 7.35 (d, *J* = 8.4 Hz, 2H), 6.97 (d, *J* = 8.3 Hz, 2H), 4.10 (s, 1H), 3.43 (s, 6H), 2.19 (s, 3H) ppm. **^{13}C NMR** (76 MHz, DMSO-d_6) δ 165.7, 138.8, 129.1, 128.8, 118.0, 67.0, 58.8, 41.3, 20.3 ppm. **IR (neat)**: ν (cm^{-1}) = 3340, 3030, 3017, 2925, 1738, 1619, 1583, 1512, 1364, 1137, 1047, 817; **HRMS** (ESI): calcd for $C_{11}H_{16}O_2NS$ [M+H+]: 226.0896; found: 226.0894.

***N*-(4-Chlorophenyl)-2-(dimethyl(oxo)-λ^6-sulfaneylidene)acetamide (1o)**

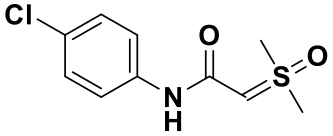

Prepared according to general procedure A from 4-chlorophenyl isocyanate (1.04 g, 6.8 mmol). White solid (818 mg, 49%) *Rf*= 0.06 (10:1 $CHCl_3$:MeOH). mp=178±2 ºC. **^{1}H NMR** (300 MHz, DMSO-d_6) δ = 8.88 (s, 1H), 7.52 -7.47 (m, 2H), 7.22 – 7.17 (m, 2H), 4.14 (s, 1H), 3.44 (s, 6H) ppm. **^{13}C NMR** (76 MHz, DMSO-d_6) δ 165.7, 140.4, 128.2, 123.8, 119.2, 59.4, 41.3 ppm. **IR (neat)**: ν (cm^{-1}) = 3335, 3075, 3015, 2924, 1617, 1579, 1516, 1489, 1396, 1139; **HRMS** (ESI): calcd for $C_{10}H_{13}ClNO_2S$ [M+H+]: 246.0350; found: 246.0349.

[8] Xu, Y.; Gao, Y.; Su, L.; Wu, H.; Tian, H.; Zeng, M.; Xu, C.; Zhu, X.; Liao, K.; *Angew. Chem.Int. Ed.* **2023**, *62*, e20231363.

2-(Dimethyl(oxo)-λ^{6}-sulfaneylidene)-*N*-(4-methoxyphenyl)acetamide (1p)

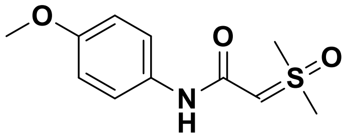

Prepared according to general procedure A from 4-methoxyphenyl isocyanate (777 µL, 6.8 mmol). White solid (705 mg, 43%) *Rf*= 0.07 (9:1 $CHCl_3$:MeOH). mp=182±2 ºC. **^{1}H NMR** (300 MHz, DMSO-d_6) δ = 8.54 (s, 1H), 7.40 -7.31 (m, 2H), 6.80 - 6.72 (m, 2H), 4.04 (s, 1H), 3.68 (s, 3H), 3.42 (s, 6H) ppm. **^{13}C NMR** (76 MHz, DMSO-d_6) δ 165.7, 153.5, 134.6, 119.5, 113.6, 58.4, 55.1, 41.4 ppm. **IR (neat)**: ν (cm^{-1}) = 3335, 3075, 3015, 2924, 1617, 1579, 1516, 1489, 1396, 1139; **HRMS** (ESI): calcd for $C_{11}H_{16}O_3NS$ [M+H+]: 242.0845; found: 242.0844.

2-(Dimethyl(oxo)-λ^{6}-sulfaneylidene)-*N*-(4-(trifluoromethyl)phenyl)acetamide (1q)

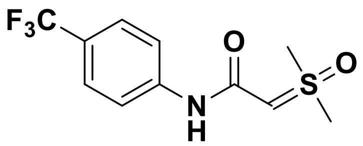

Prepared according to general procedure A from 4-trifluorotolyl isocyanate (971 µL, 6.8 mmol). White solid (854 mg, 45%) *Rf*= 0.18 (10:1 $CHCl_3$:MeOH). mp=196±2 ºC. **^{1}H NMR** (300 MHz, DMSO-d_6) δ = 7.67 (d, *J* = 8.5 Hz, 2H), 7.50 (d, *J* = 8.5 Hz, 2H), 4.22 (s, 1H), 3.47 (s, 6H) ppm. **^{13}C NMR** (76 MHz, DMSO-d_6) δ 165.7, 145.1, 126.6, 125.7, 123.0, 120.4, 120.0, 117.2, 67.0, 60.1, 41.1 ppm. **^{19}F NMR** (282 MHz, DMSO-d_6) δ -55.1 ppm. **IR (neat)**: ν (cm^{-1}) = 3341, 3075, 3018, 2970, 2930, 1738, 1623, 1589, 1514, 1370, 1140, 1100, 1066, 1047, 833; **HRMS** (ESI): calcd for $C_{11}H_{13}F_3O_2NS$ [M+H+]: 280.0613; found: 280.0611.

(Dimethyl(oxo)-λ^{6}-sulfaneylidene)(methylsulfonyl)methane (1r)

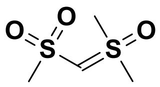

Prepared according to general procedure A from methanosulfonyl chloride (526µL, 6.8 mmol). The spectroscopy data were in good agreement with the literature.[9] White solid (752 mg, 65%) *Rf*= 0.30 (10:1 $CHCl_3$: MeOH). **^{1}H NMR** (300 MHz, $CDCl_3$) δ 4.21 (s, 1H), 3.44 (s, 6H), 3.10 (s, 3H) ppm.

[9] Truce, W. E.; Madding, G.D.; *Tetrahedron. Lett*. **1966**, *31*, 3681-3687.

General procedure B: synthesis of mesityl(2,2,2-trifluoroethyl)iodonium salts

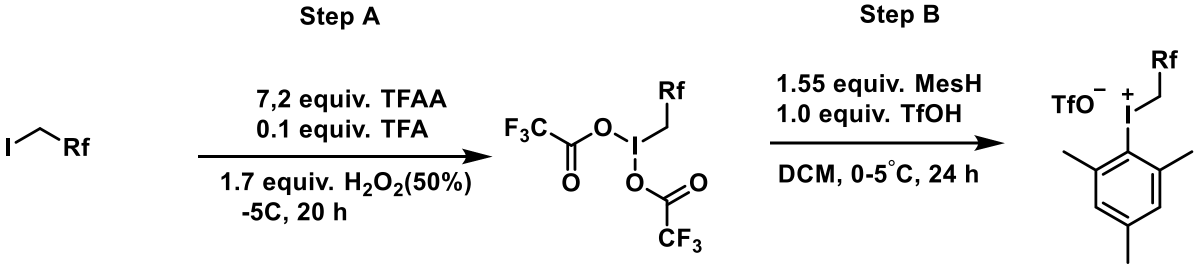

STEP A

Into a 20 mL round-bottom flask 2,2,2-trifluoroacetic anhydride (7.2 mL, 52 mmol, 6.5 equiv) and trifluoroacetic acid (62 µL, 10 mol %, 0.8 mmol, 0.1 equiv) was measured and the mixture was cooled to 0 °C. H_2O_2 (1.3 mL, 30% aqueous solution, 11.8 mmol, 1.5 equiv) was added dropwise and the mixture was stirred for 5 minutes. 1,1,1-trifluoro-2-iodoethane (788 µL, 8 mmol) was added, then the reaction was allowed to warm up to room temperature and it was stirred for 20 hours. The volatile was evaporated under reduced pressure and white oil was obtained, which crystallized in refrigerator and gave the white solid in quantitative yield.

STEP B

In a 100 mL round-bottom flask (2,2,2-trifluoroethyl)-λ^3-iodanediyl bis(2,2,2-trifluoroacetate) (3.48 g, 8 mmol, 1.0 equiv) was dissolved in dichloromethane (13–15 mL), then the solution was cooled to 0 °C and mesitylene (1.25 mL, 12 mmol, 1.5 equiv) was added. Then trifluoromethanesulfonic acid (706 µL, 8 mmol) was added dropwise. The reaction mixture turned ***to a dark red solution*** and it was kept at 0 °C for 24 hours. The solvent was evaporated under reduced pressure, then diethyl ether was added. White crystals precipitated from the mixture. The product was filtered off and washed with diethyl ether and white solid was obtained.

Mesityl(2,2,2-trifluoroethyl)-λ^3-iodaneyl trifluoromethanesulfonate (2a)

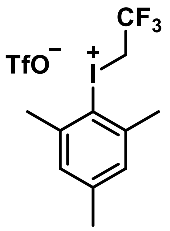

Prepared according to general procedure B. The spectroscopy data were in good agreement with the literature.[10] White solid (2.67 g, 70%). **^{1}H NMR** (300 MHz, DMSO-d$_6$) δ= 6.96-9.95 (m, 2H), 5.00 (q, J = 8.7 Hz, 2H), 2.36 (s, 6H), 2.19 (s, 3H) ppm. **^{19}F NMR** (283 MHz, DMSO-d$_6$) δ = -73.03, -77.77 ppm.

[10] G. L. Tolnai, A. Székely, Z. Makó, T. Gáti, J. Daru, T. Bihari, A. Stirling, Z. Novák *Chem. Commun.* **2015**, *51*, 4488-4491.

Mesityl(2,2,3,3-tetrafluoropropyl)-λ^3-iodaneyl trifluoromethanesulfonate (2b)

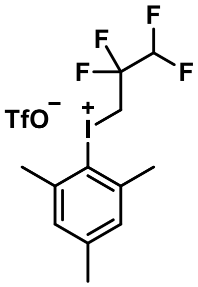

Prepared according to general procedure B. The spectroscopy data were in good agreement with the literature.[11] White solid (2.44 g, 60%). **^{1}H NMR** (300 MHz, CD_2Cl_2) δ= 6.90 (s, 1H), 6.00 (tt, *J* = 52.7, 3.1 Hz, 1H), 4.80 (tt, *J* = 12.0, 1.3 Hz, 2H), 2.42 (s, 6H), 2.23 (s, 3H) ppm. **^{19}F NMR** (283 MHz, CD_2Cl_2) δ = -74.41, -123.73 (t, *J* = 12.0 Hz), -137.01 (d, *J* = 52.4 Hz) ppm.

[11] Tóth, B.L., Sályi, G.; Domján, A., Egyed, O., Bényei, A., Gonda, Z., Novák, Z. *Adv. Synth. Catal.* **2022**, *364*, 348–354.

Table S1. Optimization of the reaction conditions.

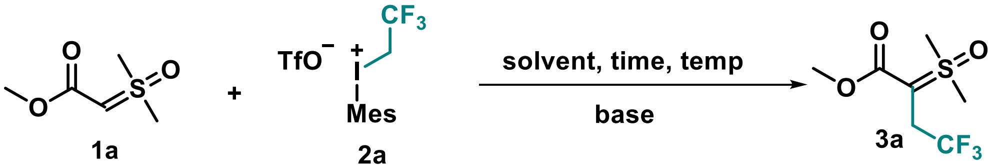

Entry[a]	Solvent	Temp	Time	Concentr.	Base	Yield 3a(%)
**1**	DCM	RT	24 h	0.5 M	Cs_2CO_3	7
**2**	DCE	RT	24 h	0.5 M	Cs_2CO_3	2
**3**	THF	RT	24 h	0.5 M	Cs_2CO_3	NR
**4**	AcOEt	RT	24 h	0.5 M	Cs_2CO_3	9
**5**	ACN	RT	24 h	0.5 M	Cs_2CO_3	69
**6**	ACN	RT	6 h	0.5 M	Cs_2CO_3	60
**7**	ACN	50 ºC	1h	0.5 M	Cs_2CO_3	46
**8**	ACN	60 ºC	30 min	0.5 M	Cs_2CO_3	49
**9**	ACN	RT	6h	0.5 M	Na_2CO_3	27
**10**	ACN	RT	6h	0.5 M	K_3PO_4	48
**11**	ACN	RT	6h	1.0 M	Cs_2CO_3	56
**12**	ACN	RT	6 h	0.5 M	–	8
**13**	Dioxane	RT	6 h	0.5 M	Cs_2CO_3	NR
**14**	Et_2O	RT	6 h	0.5 M	Cs_2CO_3	22
**15**	ACN	70 ºC(μW)	10 min	0.5 M	Cs_2CO_3	74
**16**	DCE	70 ºC(μW)	10 min	0.5 M	Cs_2CO_3	62
**17**	DMSO	70 ºC(μW)	10 min	0.5 M	Cs_2CO_3	15
**18**	AcOEt	70 ºC(μW)	10 min	0.5 M	Cs_2CO_3	70
**19**	TFE	70 ºC(μW)	10 min	0.5 M	Cs_2CO_3	2

**20**	MTBE	70 ºC(μW)	10 min	0.5 M	Cs_2CO_3	61
**21**	DMF	70 ºC(μW)	10 min	0.5 M	Cs_2CO_3	10
**23**	ACN	80 ºC(μW)	10 min	0.5 M	Cs_2CO_3	71
**24**	ACN	50 ºC(μW)	30 min	0.5 M	Cs_2CO_3	72
**25**	ACN	60 ºC(μW)	20 min	0.5 M	Cs_2CO_3	74
**26**	ACN	70 ºC(μW)	10 min	0.5 M	$(iPr)_2EtN$	45
**27**	ACN	70 ºC(μW)	10 min	0.5 M	Et_3N	24
**28**	ACN	70 ºC(μW)	10 min	0.5 M	DABCO	34
**34**[b]	ACN	70 ºC (μW)	10 min	1.0 M	Cs_2CO_3	79(75)
**35**[c]	ACN	70 ºC (μW)	10 min	0.5 M	Cs_2CO_3	60
**36**[d]	ACN	70 ºC (μW)	10 min	0.5 M	Cs_2CO_3	67
**37**[e]	ACN	70 ºC (μW)	10 min	0.5 M	Cs_2CO_3	73
**38**[f]	ACN	70 ºC (μW)	10 min	0.5 M	Cs_2CO_3	65
**39**[g]	ACN	70 ºC (μW)	10 min	0.5 M	Cs_2CO_3	76
**40**[h]	ACN	70 ºC (μW)	10 min	0.5 M	Cs_2CO_3	53
**41**[b,i]	ACN	70 ºC (μW)	10 min	1.0 M	Cs_2CO_3	68

a- Reaction performed with **1a** (0.1 mmol, 1.0 equiv.), **2a** (0.13 mmol, 1.3 equiv.) and **base** (0.13 mmol, 1.3 equiv.). *b*- 2.0 equiv. of **2a** and 1.0 equiv. of Cs_2CO_3. *c*- 0.5 equiv. of Cs_2CO_3 and 1.3 equiv. of **2a**. *d*- 2.0 equiv. of the Cs_2CO_3. *e*- 2.0 equiv. of **2a**. *f*- 1.0 equiv. of **2a**. *g*- 1.0 equiv. of Cs_2CO_3. *h*- 2.0 equiv. of **1a** and 1.0 equiv. of **2a**. *i*- Reaction performed without inert atmosphere.

Frequently asked questions (FAQ's)

Q: Can the reaction be performed without argonium atmosphere?

A: Yes, without an inert atmosphere the product is formed, however with lower yield (table S1, entry 41).

Q: Why do aromatic keto or iminosulfoxonium ylides doesn't work for this transformation?

A: Ketosulfoxonium (iminosulfoxonium included) ylides are less nucleophilic than the ester ones, we believe that the optimized reaction conditions are not suitable for these ylides. Please see the computational studies.

Q: What is the appearance of the reaction when the reaction is finished?

A: The reaction mixture appears heterogenous with a pale-yellow solution with a white precipitate (Figure S1).

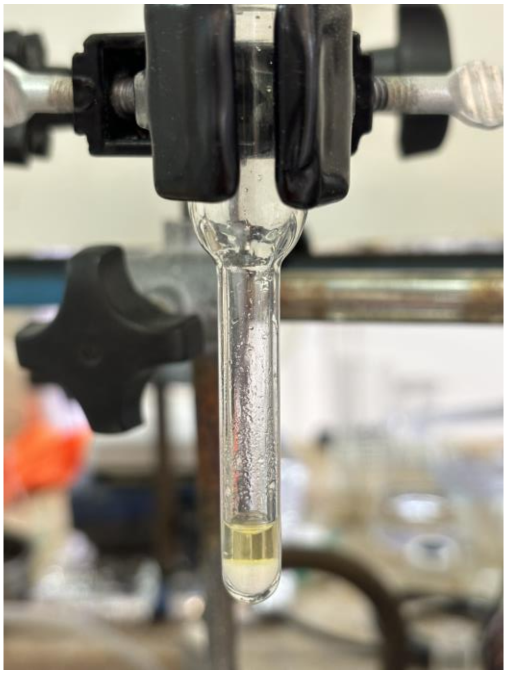

Figure S1. Reaction appearance once is finished.

General procedure C: 2,2,2-trifluoroethylation of sulfoxonium ylides

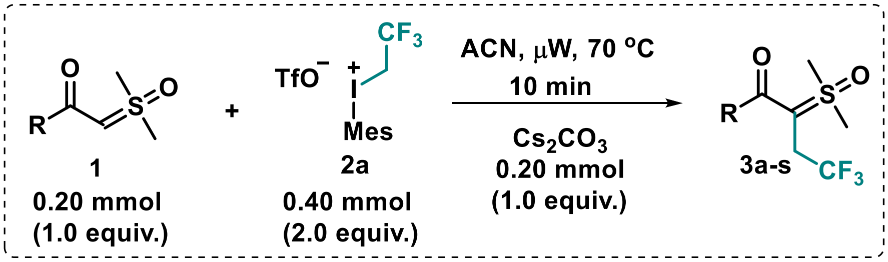

An oven dried 5 mL microwave flask containing a magnetic stirrer was charged with Cs_2CO_3 (70.5 mg, 0.2 mmol, 1.0 equiv), sulfoxonium ylide (0.2 mmol, 1.0 equiv) and the corresponding fluoroethyl iodonium salt (0.40 mmol, 2.0 equiv). The reaction vessel was capped with a rubber septum and filled with nitrogen. Then ACN (0.2 mL) was added. The rubber septum was removed and the microwave vial was quickly capped with a Teflon microwave cap. The reaction was heated to 70 ºC for 10 min. The crude mixture was dissolved with DCM (3 mL), the solvent was removed under reduced pressure to furnish a crude product that was purified by flash column chromatography, using silica gel 60 (200–400 mesh) as a stationary phase (eluent *n*-hex/AcOEt 5:95%).

1 mmol reaction scale procedure: To a 20 mL microwave glass vial equipped with a magnetic stir bar was added Cs_2CO_3 (325 mg, 1.0 mmol, 1.0 equiv), sulfoxonium ylide (1.0 mmol, 1.0 equiv) and the corresponding fluoroethyl iodonium salt (2.0 mmol, 2.0 equiv). The reaction vessel was capped with a rubber septum and filled with nitrogen. Then ACN (1.0 mL) was added. The rubber septum was removed and the microwave vial was quickly capped with a Teflon microwave cap. The reaction was heated to 70 ºC for 10 min. The crude mixture was dissolved with DCM (10 mL), the solvent was removed under reduced pressure to furnish a crude product that was purified by flash column chromatography, using silica gel 60 (200–400 mesh) as a stationary phase (eluent *n*-hex/AcOEt 5:95%).

Methyl 2-(dimethyl(oxo)-λ^6-sulfaneylidene)-4,4,4-trifluorobutanoate (3a)

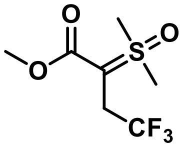

Prepared according to the general procedure C from sulfoxonium ylide **1a** (30.0 mg, 0.2 mmol, 1.0 equiv) and mesitylene iodoarene **2j** (191.3 mg, 0.40 mmol, 2.0 equiv). Purification by flash column chromatography in AcOEt:*n*-Hex as eluent (80:20). Isolated as a white solid (36.7 mg, 79%). *Rf*= 0.40 (5%MeOH: DCM). mp=74±2 ºC. **^{1}H NMR** (300 MHz, $CDCl_3$) δ 3.63 (s, 3H), 3.38 (s, 6H), 3.07 (q, *J* = 10.2 Hz, 2H) ppm; **^{13}C{^{1}H, ^{19}F} NMR** (151 MHz, $CDCl_3$) δ 167.2, 126.6, 50.2, 49.8, 43.3, 27.8 ppm; **^{19}F NMR** (282 MHz, $CDCl_3$) δ -68.6 ppm. **IR** (neat): ν (cm^{-1}) = 3018, 2931, 2927, 1739, 1613, 1444, 1351, 1113, 1026, 759; **HRMS (ESI):** calcd for $C_7H_{13}F_3O_3S$ [M+H$^+$]: 233.0459; found: 233.0452.

Ethyl 2-(dimethyl(oxo)-λ^6-sulfaneylidene)-4,4,4-trifluorobutanoate (3b)

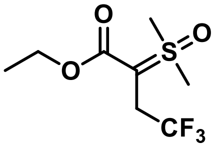

Prepared according to the general procedure C from sulfoxonium ylide **1b** (32.8 mg, 0.2 mmol, 1.0 equiv) and mesitylene iodoarene **2j** (191.3 mg, 0.40 mmol, 2.0 equiv). Purification by flash column chromatography in AcOEt:*n*-Hex as eluent (80:20). Isolated as a yellow solid (36.9 mg, 75%). *Rf*= 0.41 (5%MeOH: DCM). mp=70±2 ºC. **^{1}H NMR** (300 MHz, $CDCl_3$) δ 4.11 – 4.03 (m, 1H), 3.37 (s, 3H), 3.07 (q, *J* = 10.2 Hz, 2H), 1.23 (t, *J* = 7.1 Hz, 2H) ppm; **^{13}C{^{1}H, ^{19}F} NMR** (76 MHz, $CDCl_3$) δ 167.0, 126.7, 58.9, 50.3, 43.5, 27.9, 14.8 ppm; **^{19}F NMR** (282 MHz, $CDCl_3$) δ -68.6 ppm. **IR** (neat): ν (cm^{-1}) = 3016, 2946, 2929, 1737, 1609, 1374,1334, 1198, 1118, 1023, 731; **HRMS (ESI):** calcd for $C_8H_{14}F_3O_3S$ [M+H$^+$]: 247.0615; found: 247.0609.

Isobutyl 2-(dimethyl(oxo)-λ^6-sulfaneylidene)-4,4,4-trifluorobutanoate (3c)

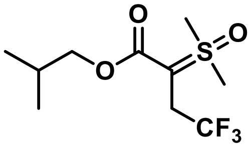

Prepared according to the general procedure C from sulfoxonium ylide **1c** (38.4 mg, 0.2 mmol, 1.0 equiv) and mesitylene iodoarene **2j** (191.3 mg, 0.40 mmol, 2.0 equiv). Purification by flash column chromatography in AcOEt:*n*-Hex as eluent (80:20). Isolated as a pale-yellow solid (46.1 mg, 84%). *Rf*= 0.40 (5%MeOH: DCM). mp=70±2 ºC. **^{1}H NMR** (300 MHz, $CDCl_3$) δ 3.78 (d, *J* = 6.5 Hz, 2H), 3.37 (s, 6H), 3.07 (q, *J* = 10.2 Hz, 2H), 1.89 (dt, *J* = 13.3, 6.7 Hz, 1H), 0.92 (d, *J* = 6.7 Hz, 6H).ppm; **^{13}C {^{1}H, ^{19}F} NMR** (76 MHz, $CDCl_3$) δ 167.0, 126.7, 69.3, 50.2, 43.5, 28.2, 27.9, 19.2 ppm; **^{19}F NMR** (282 MHz, $CDCl_3$) δ -68.5 ppm. **IR** (neat): ν (cm^{-1}) = 3015, 2961, 2930, 2893, 2877, 1738, 1608, 1375, 1330, 1120, 1016, 987; **HRMS (ESI):** calcd for $C_{10}H_{18}F_3O_3S_2$ [M+H$^+$]: 275.0928; found: 275.0920.

Hexyl 2-(dimethyl(oxo)-λ^6-sulfaneylidene)-4,4,4-trifluorobutanoate (3d)

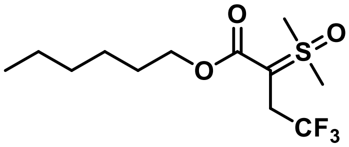

Prepared according to the general procedure C from sulfoxonium ylide **1d** (44.1 mg, 0.2 mmol, 1.0 equiv) and mesitylene iodoarene **2j** (191.3 mg, 0.40 mmol, 2.0 equiv). Purification by flash column chromatography in AcOEt:*n*-Hex as eluent (80:20). Isolated as a pale-yellow solid (41.1 mg, 68%). *Rf*= 0.42 (5%MeOH: DCM). mp=59±2 ºC. **^{1}H NMR** (300 MHz, $CDCl_3$) δ 4.00 (t, *J* = 6.6 Hz, 2H), 3.37 (s, 6H), 3.06 (q, *J* = 10.2 Hz, 2H), 1.58 (q, *J* = 6.8 Hz, 2H), 1.30 (ddq, *J* = 9.6, 7.3, 3.6 Hz, 6H), 0.87 (m, *J* = 7.1 Hz, 3H) ppm; **^{13}C {^{1}H, ^{19}F} NMR** (76 MHz, $CDCl_3$) δ 167.1, 126.7, 63.2, 50.2, 43.5, 31.6, 29.2, 27.9, 25.7, 22.7, 14.1 ppm; **^{19}F NMR** (282 MHz, $CDCl_3$) δ -68.6 ppm. **IR** (neat): ν (cm^{-1}) = 3014, 2928, 2859, 1611, 1332, 1120, 1017; **HRMS (ESI):** calcd for $C_{12}H_{22}F_3O_3S$ [M+H$^+$]: 303.1241; found: 303.1236.

Dodecyl 2-(dimethyl(oxo)-λ^6-sulfaneylidene)-4,4,4-trifluorobutanoate (3e)

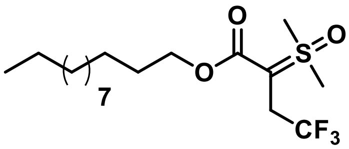

Prepared according to the general procedure C from sulfoxonium ylide **1e** (60.9 mg, 0.2 mmol, 1.0 equiv) and mesitylene iodoarene **2j** (191.3 mg, 0.40 mmol, 2.0 equiv). Purification by flash column chromatography in AcOEt:*n*-Hex as eluent (80:20). Isolated as a white solid (62.6 mg, 81%). *Rf*= 0.44 (5%MeOH: DCM). mp=61±2 ºC. **^{1}H NMR** (300 MHz, $CDCl_3$) δ 3.99 (t, *J* = 6.6 Hz, 2H), 3.37 (s, 6H), 3.06 (q, *J* = 10.2 Hz, 2H), 1.65 – 1.52 (m, 2H), 1.38 – 1.21 (m, 18H), 0.86 (t, *J* = 6.9 Hz, 3H) ppm; **^{13}C {^{1}H, ^{19}F} NMR** (76 MHz, $CDCl_3$) δ 167.1, 126.7, 63.2, 50.2, 43.5, 32.0, 29.8, 29.7, 29.5, 29.4, 29.2, 27.9, 26.1, 22.8, 14.2 ppm; **^{19}F NMR** (282 MHz, $CDCl_3$) δ -68.6 ppm. **IR** (neat): ν (cm^{-1}) 3016, 2943, 2922, 2853, 1738, 1611, 1334, 1124; **HRMS (ESI):** calcd for $C_{18}H_{34}F_3O_3S$ [M+H$^+$]: 387.2180; found: 387.2175.

***tert*-butyl 2-(dimethyl(oxo)-λ^6-sulfaneylidene)-4,4,4-trifluorobutanoate (3f)**

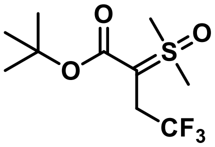

Prepared according to the general procedure C from sulfoxonium ylide **1f** (38.4 mg, 0.2 mmol, 1.0 equiv) and mesitylene iodoarene **2j** (191.3 mg, 0.40 mmol, 2.0 equiv). Purification by flash column chromatography in AcOEt:*n*-Hex as eluent (80:20). Isolated as a white solid (44.9 mg, 82%). *Rf*= 0.38 (5%MeOH: DCM). mp=112±2 ºC. **^{1}H NMR** (300 MHz, $CDCl_3$) δ 3.35 (s, 6H), 3.02 (q, *J* = 10.2 Hz, 2H), 1.43 (s, 9H) ppm; **^{13}C {^{1}H, ^{19}F} NMR** (76 MHz, $CDCl_3$) δ 167.1, 126.9, 78.9, 50.8, 43.7, 28.8, 28.4 ppm; **^{19}F NMR** (282 MHz, $CDCl_3$) δ -68.6 ppm. **IR** (neat): ν (cm^{-1}) = 3007 2924 1738 1602 1356 1120 1021 980; **HRMS (ESI):** calcd for $C_{10}H_{18}F_3O_3S$ [M+H$^+$]: 275.0920; found: 275.0928.

Cyclopentyl 2-(dimethyl(oxo)-λ^6-sulfaneylidene)-4,4,4-trifluorobutanoate (3g)

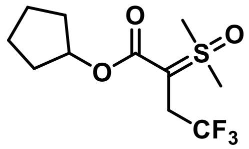

Prepared according to the general procedure C from sulfoxonium ylide **1g** (40.8 mg, 0.2 mmol, 1.0 equiv) and mesitylene iodoarene **2j** (191.3 mg, 0.40 mmol, 2.0 equiv). Purification by flash column chromatography in AcOEt:*n*-Hex as eluent (80:20). Isolated as a white solid (34.3 mg, 60%). *Rf*= 0.39 (5%MeOH: DCM). mp=110±2 ºC. **^{1}H NMR** (300 MHz, $CDCl_3$) δ 5.19 – 5.07 (m, 1H), 3.37 (s, 6H), 3.04 (q, *J* = 10.2 Hz, 2H), 1.84 – 1.64 (m, 6H), 1.61 – 1.48 (m, 2H) ppm; **^{13}C {^{1}H, ^{19}F} NMR** (76 MHz, $CDCl_3$) δ 166.9, 126.7, 75.4, 50.4, 43.6, 33.1, 27.9, 23.9 ppm; **^{19}F NMR** (282 MHz, $CDCl_3$) δ -68.6 ppm. **IR** (neat): ν (cm^{-1}) = 3020, 2964, 2934, 2874, 1623, 1377, 1334, 1313, 1129, 1025; **HRMS (ESI):** calcd for $C_9H_{17}O_3S$ [M+H$^+$]: 205.0892; found: 205.0892.

Allyl 2-(dimethyl(oxo)-λ^6-sulfaneylidene)-4,4,4-trifluorobutanoate (3h)

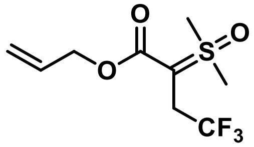

Prepared according to the general procedure C from sulfoxonium ylide **1h** (35.2 mg, 0.2 mmol, 1.0 equiv) and mesitylene iodoarene **2j** (191.3 mg, 0.40 mmol, 2.0 equiv). Purification by flash column chromatography in AcOEt:*n*-Hex as eluent (80:20). Isolated as a yellow oil (47.5 mg, 92%). *Rf*= 0.40 (5%MeOH: DCM. **^{1}H NMR** (400 MHz, $CDCl_3$) δ 5.91 (ddt, *J* = 15.8, 10.5, 5.2 Hz, 1H), 5.32 – 5.09 (m, 2H), 4.53 (d, *J* = 5.2 Hz, 2H), 3.37 (s, 6H), 3.09 (q, *J* = 10.2 Hz, 2H) ppm; **RMN ^{13}C** (126 MHz, $CDCl_3$) δ 166.5, 133.7, 126.7 (q, *J* = 278.4 Hz), 116.6, 63.6, 50.6, 43.5, 27.8 (q, *J* = 32.1 Hz).ppm; **^{19}F NMR** (282 MHz, $CDCl_3$) δ -68.5 ppm. **IR** (neat): ν (cm^{-1}) = 3010, 2975, 2923, 1735, 1620, 1343, 1200; **HRMS (ESI):** calcd for $C_9H_{13}F_3O_3S$ [M+H$^+$]: 259.0616; found: 259.0616.

Benzyl 2-(dimethyl(oxo)-λ⁶-sulfaneylidene)-4,4,4-trifluorobutanoate (3i)

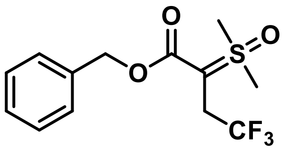

Prepared according to the general procedure C from sulfoxonium ylide **1i** (45.3 mg, 0.2 mmol, 1.0 equiv) and mesitylene iodoarene **2j** (191.3 mg, 0.40 mmol, 2.0 equiv). Purification by flash column chromatography in AcOEt:*n*-Hex as eluent (80:20). Isolated as a yellow solid (45.0 mg, 73%). *Rf*= 0.40 (5%MeOH: DCM). mp=78±2 ºC. ^{1}**H NMR** (300 MHz, $CDCl_3$) δ 7.36 (s, 1H), 7.34 (s, 1H), 7.33 – 7.25 (m, 2H), 5.10 (s, 1H), 3.39 (s, 3H), 3.13 (q, *J* = 10.2 Hz, 1H) ppm; ^{13}**C {^{1}H, ^{19}F} NMR** (76 MHz, $CDCl_3$) δ 166.6, 137.5, 128.5, 127.8, 127.6, 126.7, 64.8, 50.7, 43.5, 27.9 ppm; ^{19}**F NMR** (282 MHz, $CDCl_3$) δ -68.4 ppm. **IR** (neat): ν (cm^{-1}) = 3012, 2970, 2923, 1737, 1621, 1343, 1200, 1112; **HRMS (ESI):** calcd for $C_{13}H_{16}F_3O_3S$ [$M+H^+$]: 309.0772; found: 309.0766.

4-Nitrobenzyl 2-(dimethyl(oxo)-λ⁶-sulfaneylidene)-4,4,4-trifluorobutanoate (3j)

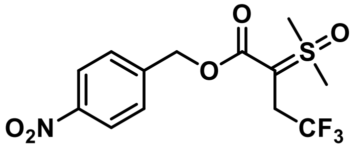

Prepared according to the general procedure C from sulfoxonium ylide **1j** (54.3 mg, 0.2 mmol, 1.0 equiv) and mesitylene iodoarene **2j** (191.3 mg, 0.40 mmol, 2.0 equiv). Purification by flash column chromatography in AcOEt:*n*-Hex as eluent (80:20). Isolated as a pale-yellow solid (43.1 mg, 61%). *Rf*= 0.39 (5%MeOH: DCM). mp=114±2 ºC. ^{1}**H NMR** (300 MHz, $CDCl_3$) δ 8.19 (d, *J* = 8.8 Hz, 2H), 7.48 (d, *J* = 8.8 Hz, 2H), 5.17 (s, 2H), 3.39 (s, 6H), 3.13 (q, *J* = 10.2 Hz, 2H) ppm; ^{13}**C {^{1}H, ^{19}F} NMR** (76 MHz, $CDCl_3$) δ 165.9, 147.4, 145.3, 127.7, 126.6, 123.8, 63.4, 51.2, 43.3, 27.8 ppm; ^{19}**F NMR** (282 MHz, $CDCl_3$) δ -68.4 ppm. **IR** (neat): ν (cm^{-1}) = 3011, 2970, 1739, 1624, 1603, 1511, 1364, 1351, 1216, 1121; **HRMS (ESI):** calcd for $C_{13}H_{15}F_3NO_5S$ [$M+H^+$]: 354.0623; found: 354.0617.

Phenyl 2-(dimethyl(oxo)-λ⁶-sulfaneylidene)-4,4,4-trifluorobutanoate (3k)

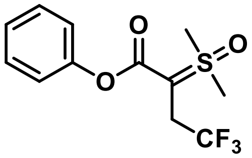

Prepared according to the general procedure C from sulfoxonium ylide **1k** (54.3 mg, 0.2 mmol, 1.0 equiv) and mesitylene iodoarene **2j** (191.3 mg, 0.40 mmol, 2.0 equiv). Purification by flash column chromatography in AcOEt:*n*-Hex as eluent (80:20). Isolated as a white solid (49.5 mg, 70%). *Rf*= 0.39 (5%MeOH: DCM). mp=108±2 ºC. **^{1}H NMR** (300 MHz, $CDCl_3$) δ 7.44 – 7.30 (m, 2H), 7.21 – 7.13 (m, 1H), 7.09 (dd, *J* = 8.6, 1.2 Hz, 2H), 3.42 (s, 6H), 3.26 (q, *J* = 10.1 Hz, 2H) ppm; **^{13}C {^{1}H, ^{19}F} NMR** (76 MHz, $CDCl_3$) δ 165.3, 151.3, 129.3, 126.7, 125.1, 122.3, 52.0, 43.1, 28.0 ppm; **^{19}F NMR** (282 MHz, $CDCl_3$) δ -68.5ppm. **IR** (neat): ν (cm^{-1}) = 3019, 2927, 1639, 1595, 1412, 1347, 1195, 1101, 729; **HRMS (ESI):** calcd for $C_{12}H_{14}F_3O_3S$ [M+H$^+$]: 295.0615; found: 295.0609.

4-Methoxyphenyl 2-(dimethyl(oxo)-λ⁶-sulfaneylidene)-4,4,4-trifluorobutanoate (3l)

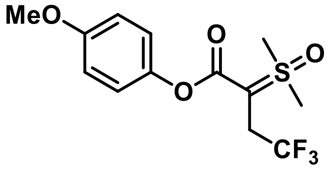

Prepared according to the general procedure C from sulfoxonium ylide **1l** (48.5 mg, 0.2 mmol, 1.0 equiv) and mesitylene iodoarene **2j** (191.3 mg, 0.40 mmol, 2.0 equiv). Purification by flash column chromatography in AcOEt:*n*-Hex as eluent (80:20). Isolated as a pale-yellow oil (51.9 mg, 80%). *Rf*= 0.37 (5%MeOH: DCM). **^{1}H NMR** (300 MHz, $CDCl_3$) δ 7.06 – 6.95 (m, 2H), 6.92 – 6.83 (m, 2H), 3.79 (s, 3H), 3.42 (s, 6H), 3.25 (q, *J* = 10.1 Hz, 2H) ppm; **^{13}C {^{1}H, ^{19}F} NMR** (76 MHz, $CDCl_3$) δ 165.6, 156.9, 144.7, 126.7, 123.1, 114.4, 55.7, 51.7, 45.9, 43.1, 28.0 ppm; **^{19}F NMR** (282 MHz, $CDCl_3$) δ -68.5ppm. **IR** (neat): ν (cm^{-1}) = 3291, 3007, 2970, 2912, 1738, 1574, 1511, 1467, 1354, 1232, 1193, 1122 ,1028; **HRMS (ESI):** calcd for $C_{13}H_{16}F_3O_4S$ [M+H$^+$]: 325.0721; found: 325.0716.

2-(Dimethyl(oxo)-λ^6-sulfaneylidene)-4,4,4-trifluoro-*N*-phenylbutanamide (3m)

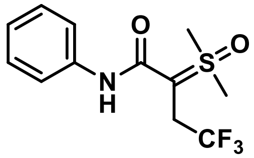

Prepared according to the general procedure C from sulfoxonium ylide **1m** (42.3 mg, 0.2 mmol, 1.0 equiv) and mesitylene iodoarene **2j** (191.3 mg, 0.40 mmol, 2.0 equiv). Purification by flash column chromatography in AcOEt:*n*-Hex as eluent (80:20). Isolated as a pale-yellow oil (29.3 mg, 50%). *Rf*= 0.35 (5%MeOH: DCM). **^{1}H NMR** (300 MHz, $CDCl_3$) δ 7.37 – 7.32 (m, 2H), 7.32 – 7.26 (m, 2H), 7.06 – 6.99 (m, 1H), 6.70 (s, 1H), 3.47 (s, 6H), 3.16 (q, *J* = 10.2 Hz, 2H) ppm; **^{13}C {^{1}H, ^{19}F} NMR** (76 MHz, $CDCl_3$) δ 165.5, 139.0, 129.0, 126.7, 123.1, 120.4, 51.9, 44.7, 28.4 ppm; **^{19}F NMR** (282 MHz, $CDCl_3$) δ -67.5 ppm. **IR** (neat): ν (cm^{-1}) = 3318, 3027, 2970, 2928, 1739, 1603, 1578, 1489, 1435, 1347, 1120, 1022; **HRMS (ESI):** calcd for $C_{12}H_{15}F_3NO_2S$ [$M+H^+$]: 294.0775; found: 294.0769.

2-(Dimethyl(oxo)-λ^6-sulfaneylidene)-4,4,4-trifluoro-*N*-(*p*-tolyl)butanamide (3n)

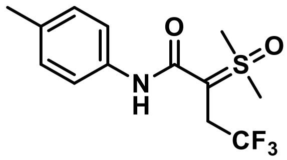

Prepared according to the general procedure c from sulfoxonium ylide **1n** (45.1 mg, 0.2 mmol, 1.0 equiv) and mesitylene iodoarene **2j** (191.3 mg, 0.40 mmol, 2.0 equiv). Purification by flash column chromatography in AcOEt:*n*-Hex as eluent (80:20). Isolated as a yellow oil (30.1 mg, 49%). *Rf*= 0.36 (5%MeOH: DCM). mp=110±2 ºC. **^{1}H NMR** (400 MHz, $CDCl_3$) δ 7.22 (d, *J* = 8.3 Hz, 2H), 7.09 (d, *J* = 8.2 Hz, 2H), 3.47 (s, 6H), 3.15 (q, *J* = 10.2 Hz, 2H), 2.29 (s, 3H) ppm; **^{13}C NMR** (126 MHz, $CDCl_3$) δ 165.4, 136.2, 132.9, 129.5, 120.7, 51.7, 28.5 (q, *J* = 31.7 Hz), 20.9 ppm; **^{19}F NMR** (282 MHz, $CDCl_3$) δ -67.5 ppm. **IR** (neat): ν (cm^{-1}) = 3321, 3027, 2975, 2920, 1739, 1738, 1600, 1578, 1491, 1344, 1183, 1116, 1089, 1023, 810; **HRMS (ESI):** calcd for $C_{13}H_{17}F_3NO_2S$ [$M+H^+$]: 308.0932; found: 308.0940.

N-(4-Chlorophenyl)-2-(dimethyl(oxo)-λ^6-sulfaneylidene)-4,4,4-trifluorobutanamide (3o)

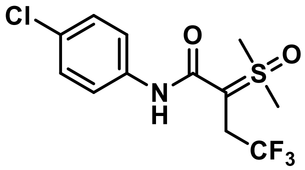

Prepared according to the general procedure c from sulfoxonium ylide **1o** (49.1 mg, 0.2 mmol, 1.0 equiv) and mesitylene iodoarene **2j** (191.3 mg, 0.40 mmol, 2.0 equiv). Purification by flash column chromatography in AcOEt:*n*-Hex as eluent (80:20). Isolated as a pale-yellow solid (31.4 mg, 48%). *Rf*= 0.36 (5%MeOH: DCM). mp=110±2 ºC. **^{1}H NMR** (300 MHz, $CDCl_3$) δ 7.31 – 7.26 (m, 2H), 7.25-7.22 (m, 2H), 6.66 (s, 1H), 3.46 (s, 6H) 3.14 (q, *J* = 10.2 Hz, 2H) ppm; **^{13}C {^{1}H, ^{19}F} NMR** (76 MHz, $CDCl_3$) δ 165.3, 137.7, 129.0, 128.0, 126.7, 121.5, 52.3, 44.7, 28.4 ppm; **^{19}F NMR** (282 MHz, $CDCl_3$) δ -67.5 ppm. **IR** (neat): ν (cm^{-1}) = 3450, 3404, 3030, 2970, 2925, 1738, 1599, 1578, 1491, 1344, 1183, 1116, 1089, 1023, 811, 745; **HRMS (ESI):** calcd for $C_{12}H_{14}ClF_3NO_2S$ [M+H$^+$]: 328.0385; found: 328.0380.

2-(Dimethyl(oxo)-λ^6-sulfaneylidene)-4,4,4-trifluoro-N-(4-methoxyphenyl)butanamide (3p)

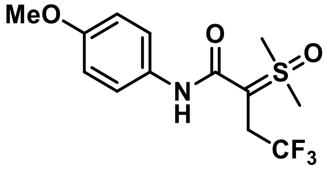

Prepared according to the general procedure C from sulfoxonium ylide **1p** (48.3 mg, 0.2 mmol, 1.0 equiv.) and mesitylene iodoarene **2j** (191.3 mg, 0.40 mmol, 2.0 equiv.). Purification by flash column chromatography in AcOEt:*n*-Hex as eluent (80:20). Isolated as a pale-yellow solid (39.4 mg, 61%). *Rf*= 0.41 (5%MeOH: DCM). mp=110±2 ºC. **^{1}H NMR** (300 MHz, $CDCl_3$) δ 7.25 – 7.20 (m, 2H), 6.88 – 6.81 (m, 2H), 6.54 (s, 1H), 3.78 (s, 3H), 3.46 (s, 6H), 3.15 (q, *J* = 10.2 Hz, 2H) ppm; **^{13}C {^{1}H, ^{19}F} NMR** (76 MHz, $CDCl_3$) δ 165.7, 156.0, 131.9, 126.8, 122.8, 114.3, 55.7, 51.2, 44.9, 28.4 ppm; **^{19}F NMR** (282 MHz, $CDCl_3$) δ -67.5 ppm. **IR** (neat): ν (cm^{-1}) = 3012, 2929, 2839, 1639, 1506, 1348, 1194, 1170, 1098, 1026, 732; **HRMS (ESI):** calcd for $C_{13}H_{17}F_3NO_3S$ [M+H$^+$]: 324.0881; found: 324.0877.

2-(Dimethyl(oxo)-λ^6-sulfaneylidene)-4,4,4-trifluoro-*N*-(4-(trifluoromethyl)phenyl) butanamide (3q)

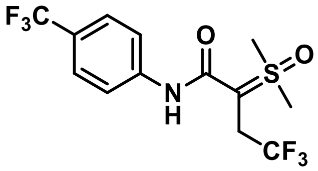

Prepared according to the general procedure C from sulfoxonium ylide **1q** (55.8 mg, 0.2 mmol, 1.0 equiv) and mesitylene iodoarene **2j** (191.3 mg, 0.40 mmol, 2.0 equiv). Purification by flash column chromatography in AcOEt:*n*-Hex as eluent (80:20). Isolated as a pale-yellow oil (32.5 mg, 45%). *Rf*= 0.41 (5%MeOH: DCM). **^{1}H NMR** (300 MHz, $CDCl_3$) δ 7.55 – 7.42 (m, 4H), 6.84 (s, 1H), 3.48 (s, 6H), 3.17 (q, *J* = 10.1 Hz, 2H) ppm; **^{13}C {^{1}H, ^{19}F} NMR** (76 MHz, $CDCl_3$) δ 165.2, 142.4, 126.6, 126.2, 124.5, 120.0, 119.4, 53.1, 44.5, 28.3 ppm; **^{19}F NMR** (282 MHz, $CDCl_3$) δ -61.9, -67.5 ppm. **IR** (neat): ν (cm^{-1}) = 3457, 3037, 1616, 1581, 1521, 1506, 1404, 1318, 1108, 1064, 1025, 1014, 730; **HRMS (ESI):** calcd for $C_{13}H_{14}F_6O_2NS$ [M+H$^+$]: 362.0649; found: 362.0642.

3-(Dimethyl(oxo)-λ^6-sulfaneylidene)-1,1,1-trifluoro-3-(methylsulfonyl)propane (3r)

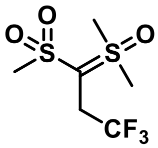

Prepared according to the general procedure C from sulfoxonium ylide **1r** (34.0 mg, 0.2 mmol, 1.0 equiv) and mesitylene iodoarene **2j** (191.3 mg, 0.40 mmol, 2.0 equiv). Purification by flash column chromatography in AcOEt:*n*-Hex as eluent (80:20). Isolated as a yellow solid (32.3 mg, 64%). *Rf*= 0.40 (5%MeOH: DCM). mp=118±2 ºC. **^{1}H NMR** (300 MHz, $CDCl_3$) δ 3.48 (s, 6H), 3.21 (q, *J* = 10.1 Hz, 2H), 3.04 (s, 3H) ppm; **^{13}C {^{1}H, ^{19}F} NMR** (76 MHz, MeOD) δ 127.7, 58.4, 47.6, 45.1, 32.2 ppm; **^{19}F NMR** (282 MHz, $CDCl_3$) δ -67.3 ppm. **IR** (neat): ν (cm^{-1}) = 3019, 2970, 2943, 1739, 1374, 1229, 1205, 1127, 1014,520; **HRMS (ESI):** calcd for $C_6H_{12}F_3O_3S_2$ [M+H$^+$]: 253.0180; found: 253.0173.

Methyl 2-(dimethyl(oxo)-λ⁶-sulfaneylidene)-4,4,5,5-tetrafluoropentanoate (3s)

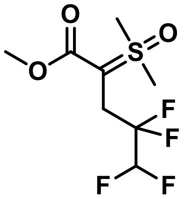

Prepared according to the general procedure C from sulfoxonium ylide **1a** (30.0 mg, 0.2 mmol, 1.0 equiv) and mesitylene iodoarene **2k** (204.1 mg, 0.40 mmol, 2.0 equiv). Purification by flash column chromatography in AcOEt:*n*-Hex as eluent (80:20). Isolated as a yellow oil (35.9 mg, 68%). *Rf*= 0.42 (5%MeOH: DCM). **^{1}H NMR** (300 MHz, $CDCl_3$) δ 5.75 (tt, *J* = 53.5, 3.9 Hz, 1H), 3.62 (s, 2H), 3.38 (s, 5H), 2.99 (t, *J* = 16.8 Hz, 2H) ppm; **^{13}C{^{1}H, ^{19}F} NMR** (151 MHz, $CDCl_3$) δ 167.3, 116.9, 110.3, 50.4, 43.4, 29.8, 25.1 ppm; **^{19}F NMR** (282 MHz, $CDCl_3$) δ -117.1, -136.5 ppm. **IR** (neat): ν (cm^{-1}) = 3025, 3001, 2943, 1748, 1613, 1445, 1341, 1185, 1112, 1091, 1017, 780; **HRMS (ESI):** calcd for $C_8H_{13}F_4O_3S$ [M+H$^+$]: 265.0515; found: 265.0514.

Synthesis of methyl 4,4,4-trifluoro-2-((4-methyl-2-oxo-2H-chromen-7-yl)thio)butanoate (4)

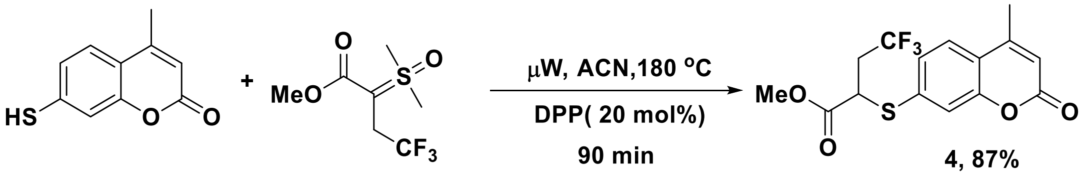

To an oven dried 5 mL microwave flask containing a magnetic stirrer was added methyl 2-(dimethyl(oxo)-λ6-sulfaneylidene)-4,4,4-trifluorobutanoate (**3a**) (34.8 mg, 0.15 mmol,1.0 equiv), 7-mercapto-4-methylcoumarin (28.8 mg, 0.15 mmol, 1.0 equiv), and diphenyl phosphate (7.5 mg, 0.03 mmol, 0.2 equiv). The reaction vessel was capped with a rubber septum and filled with nitrogen. Then ACN (0.5 mL) was added. The rubber septum was removed and the microwave vial was quickly capped with a Teflon microwave cap. The reaction was heated to 180 ºC for 90 min. The crude mixture was dissolved with DCM (3 mL), the solvent was removed under reduced pressure to furnish a crude product that was purified using Biotage IsoleraTM Prime (Snap Ultra 10g). The product was isolated as a yellow oil (45.1 mg, 87% yield), *Rf* = 0.39 (*n*-hex:AcOEt 10:1). **^{1}H NMR** (400 MHz, $CDCl_3$) δ 7.56 (d, *J* = 8.3 Hz, 1H), 7.42 (d, *J* = 1.8 Hz, 1H), 7.35 (dd, *J* = 8.3, 1.8 Hz, 1H), 6.31 (s, 1H), 3.96 (dd, *J* = 9.5, 4.5 Hz, 1H), 3.76 (s, 3H), 3.01 – 2.84 (m, 1H), 2.67 – 2.48 (m, 1H), 2.43 (d, *J* = 1.3 Hz, 3H) ppm; **^{13}C NMR** (101 MHz, $CDCl_3$) δ 170.2, 160.2, 153.6, 151.8, 136.6, 127.5, 126.6 (q, *J* = 278.2 Hz), 125.3, 120.3, 115.8, 53.2, 43.33 (q, *J* = 2.7

Hz), 36.55 (q, J = 29.7 Hz), 18.7 ppm; **^{19}F NMR** (376 MHz, $CDCl_3$) δ -65.1ppm; **IR** (neat): ν (cm^{-1}) = 3025, 3010, 2940, 1752, 1613, 1440, 1341, 1180, 1112, 1091, 780; **HRMS (ESI):** calcd for $C_{15}H_{13}F_3O_4S$ $[M+H]^+$ 347.0565, found 347.0565.

3. NMR spectra.

^{1}H NMR (300 MHz, CDCl$_3$) of compound isobutyl 2-(dimethyl(oxo)-λ^6-sulfaneylidene)acetate (**1c**).

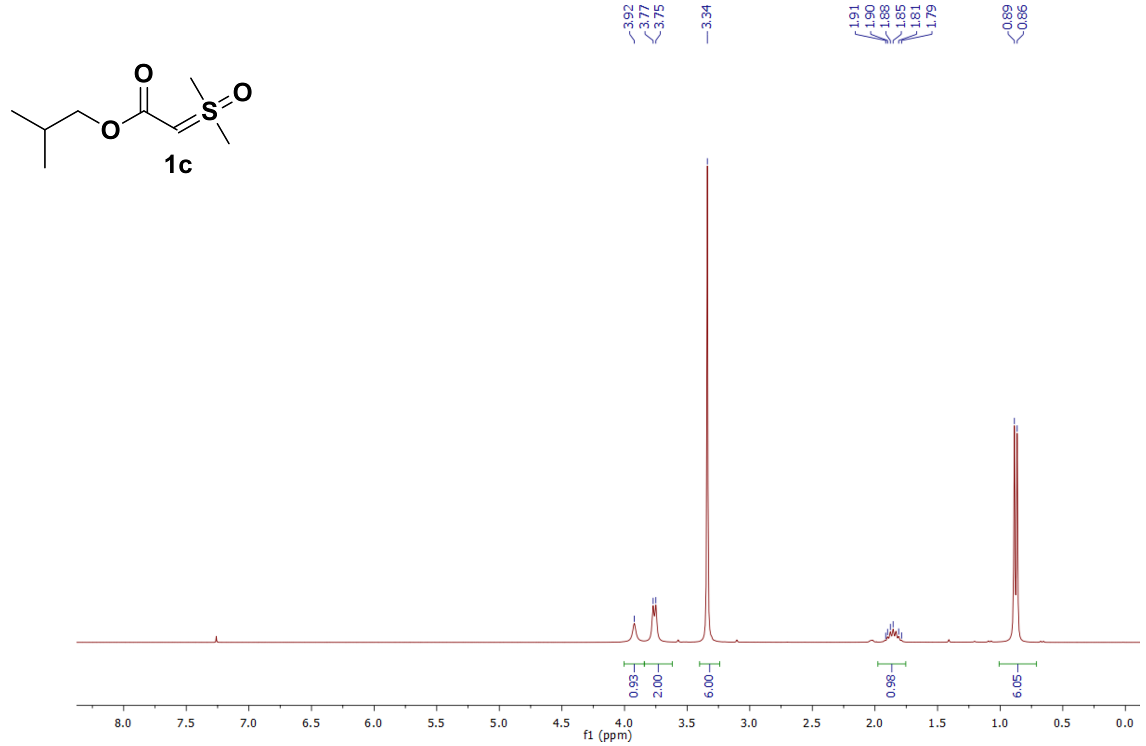

^{1}H NMR (300 MHz, CDCl3) of compound hexyl 2-(dimethyl(oxo)-λ^6-sulfaneylidene)acetate (1d).

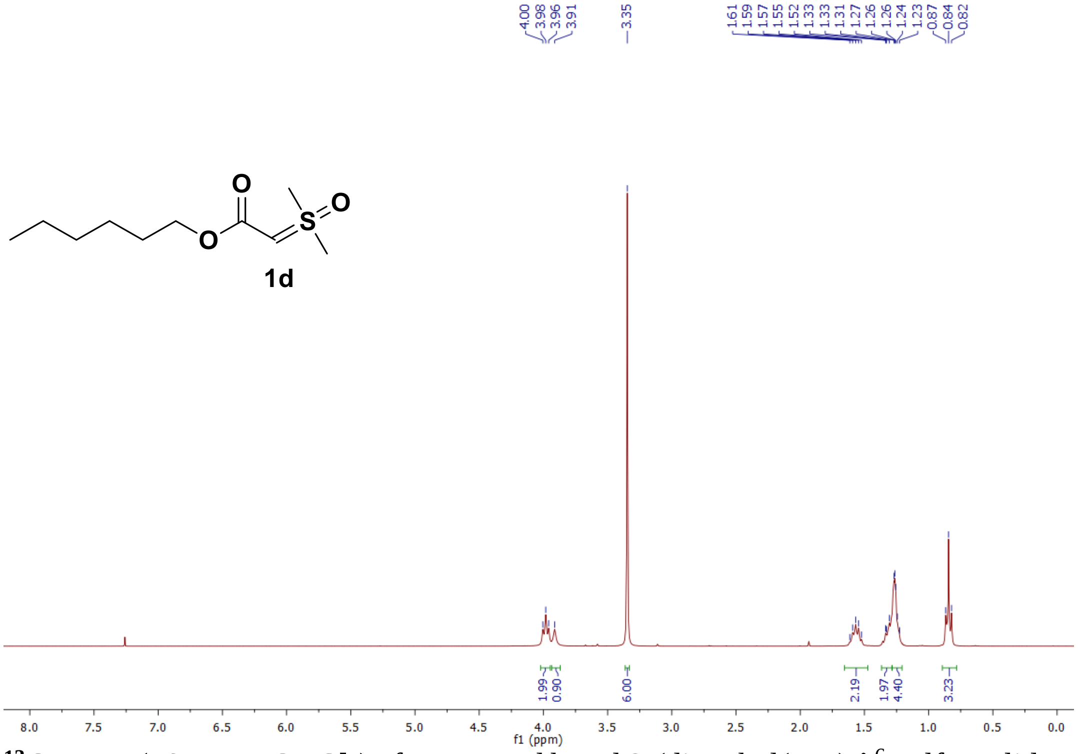

^{13}C NMR (76 MHz, CDCl3) of compound hexyl 2-(dimethyl(oxo)-λ^6-sulfaneylidene)acetate (1d).

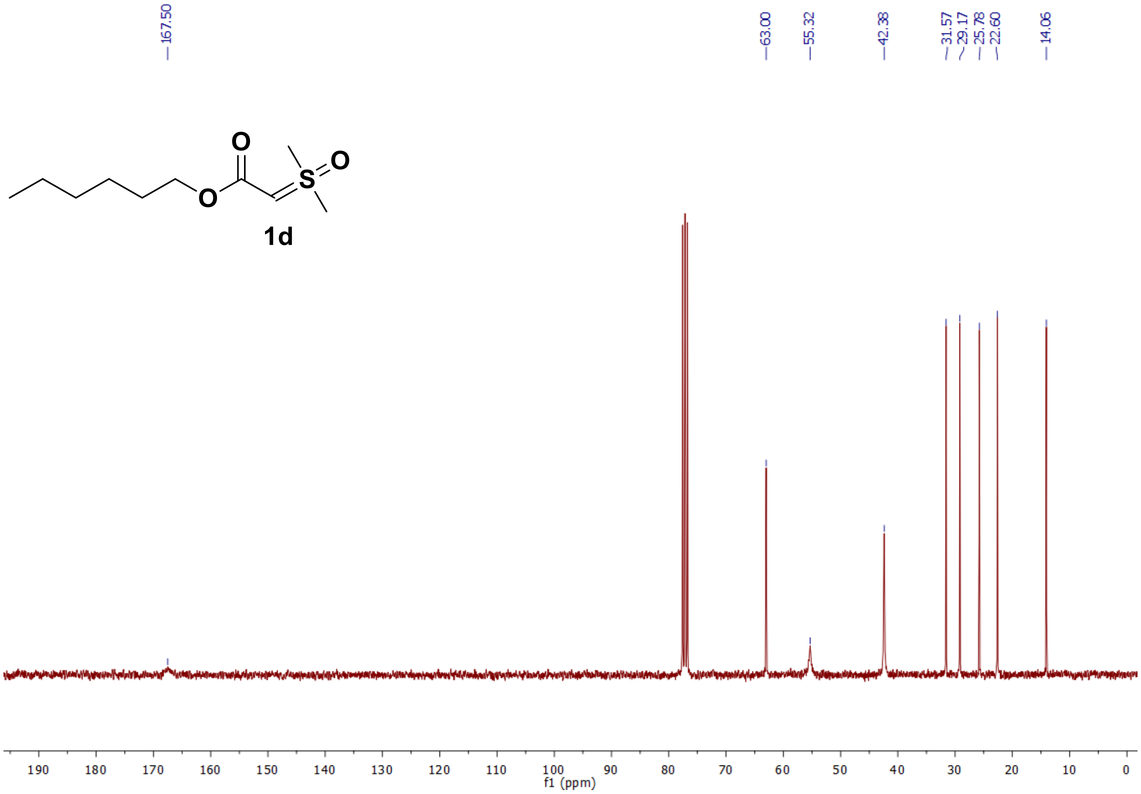

^{1}H **NMR (300 MHz, $CDCl_3$)** of compound cyclopentyl 2-(dimethyl(oxo)-λ^6-sulfaneylidene)acetate (**1g**).

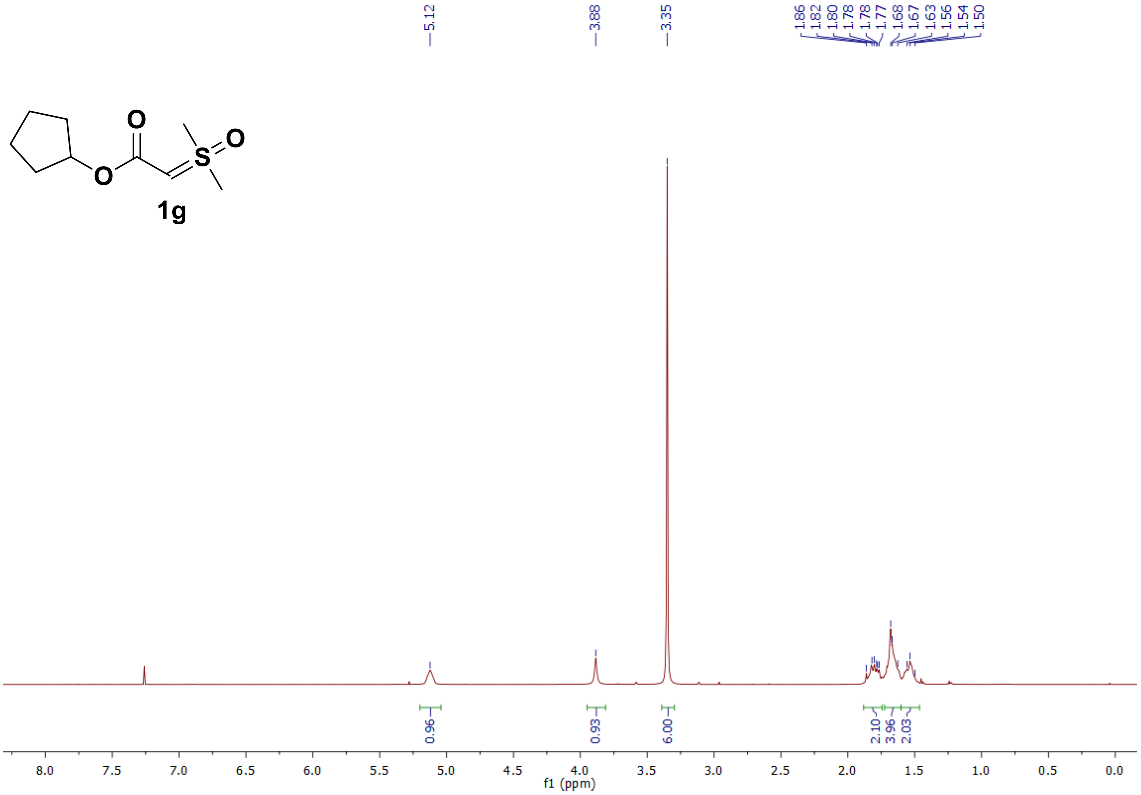

^{13}C **NMR (76 MHz, $CDCl_3$)** of compound cyclopentyl 2-(dimethyl(oxo)-λ^6-sulfaneylidene)acetate (**1g**).

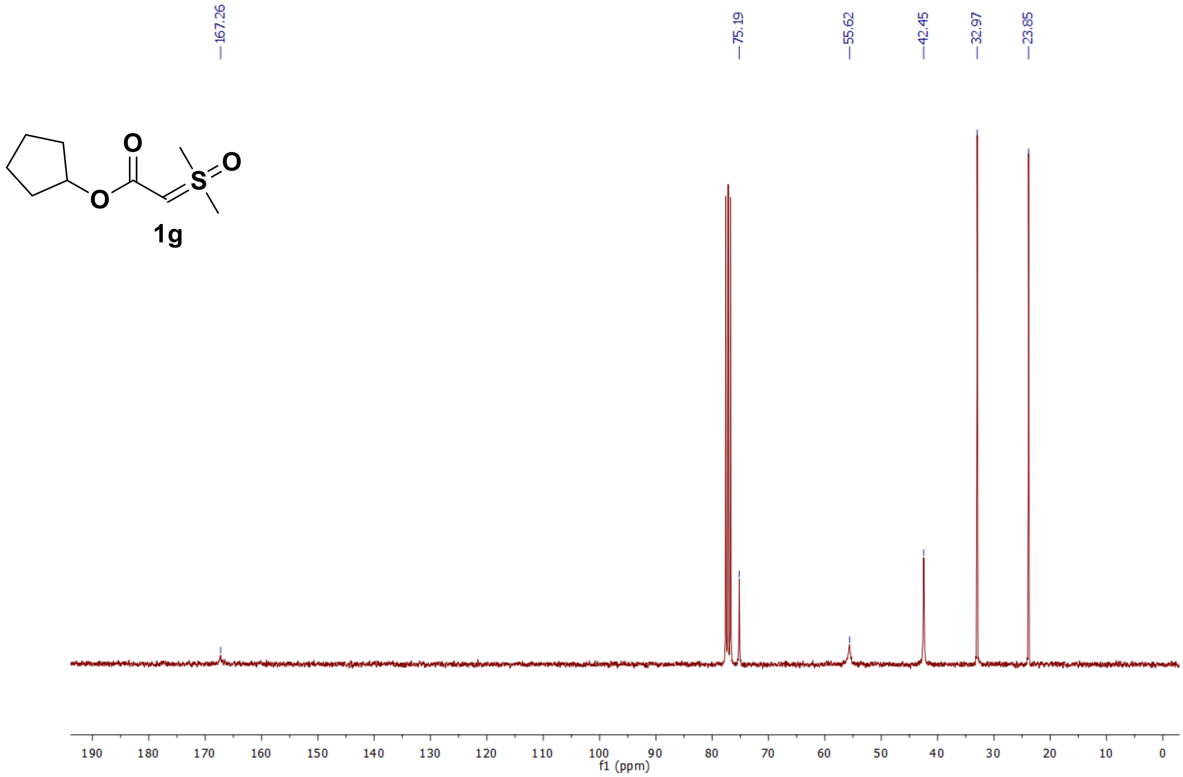

^{1}H NMR (300 MHz, $CDCl_3$) of compound allyl 2-(dimethyl(oxo)-λ^6-sulfaneylidene)acetate (**1h**).

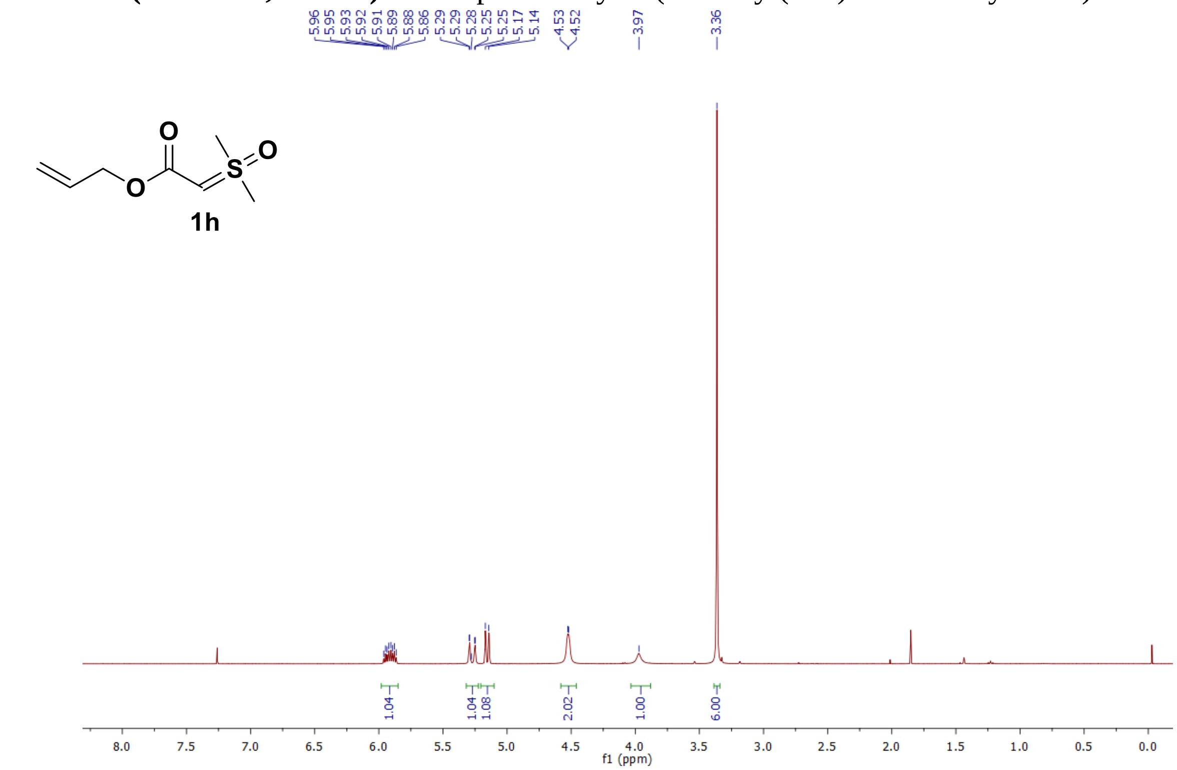

^{1}H NMR (300 MHz, $CDCl_3$) of compound 4-nitrobenzyl 2-(dimethyl(oxo)-λ^6-sulfaneylidene)acetate (**1j**).

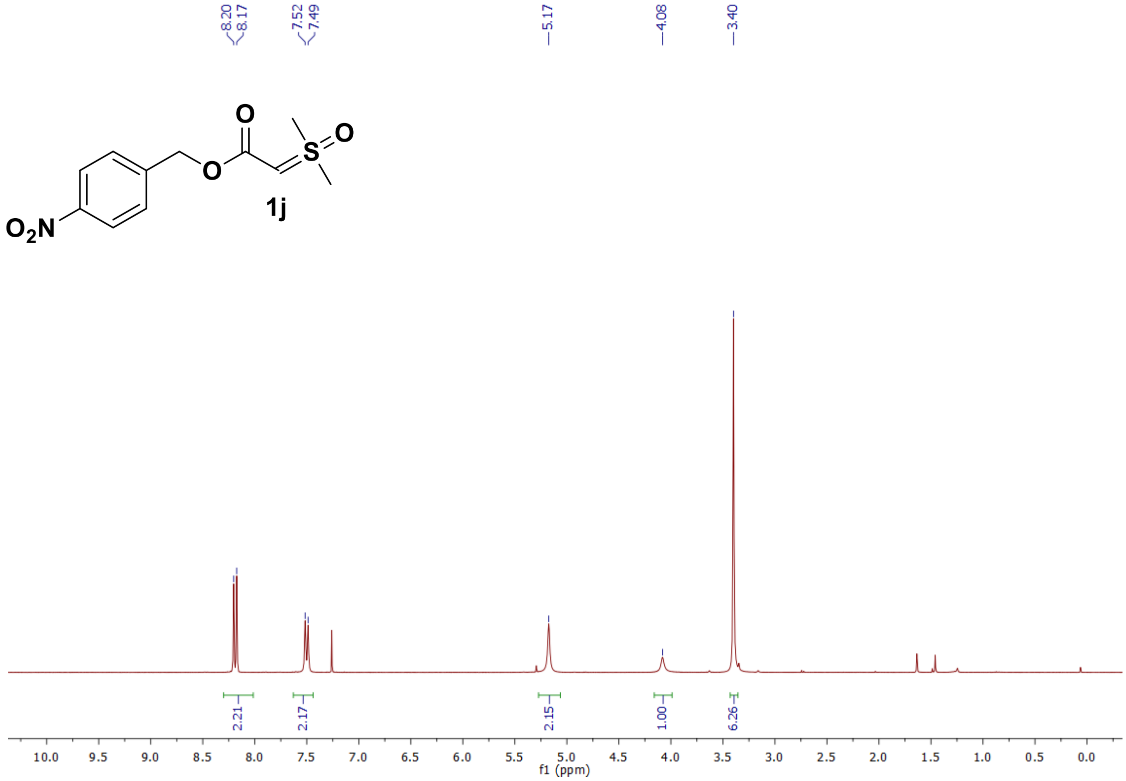

^{13}C NMR (76 MHz, CDCl$_3$) of compound 4-nitrobenzyl 2-(dimethyl(oxo)-λ^6-sulfaneylidene)acetate (**1j**).

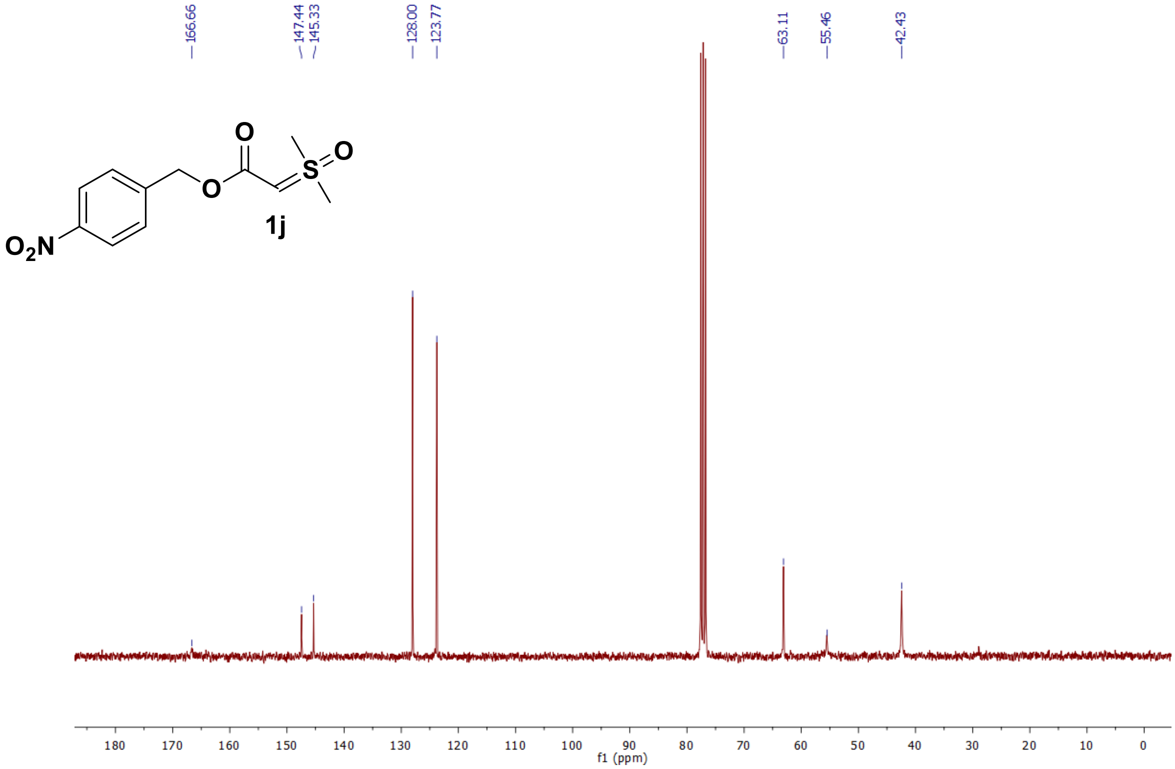

^{1}H NMR (300 MHz, CDCl$_3$) of compound 4-methoxyphenyl 2-(dimethyl(oxo)-λ^6-sulfaneylidene)acetate (**1l**).

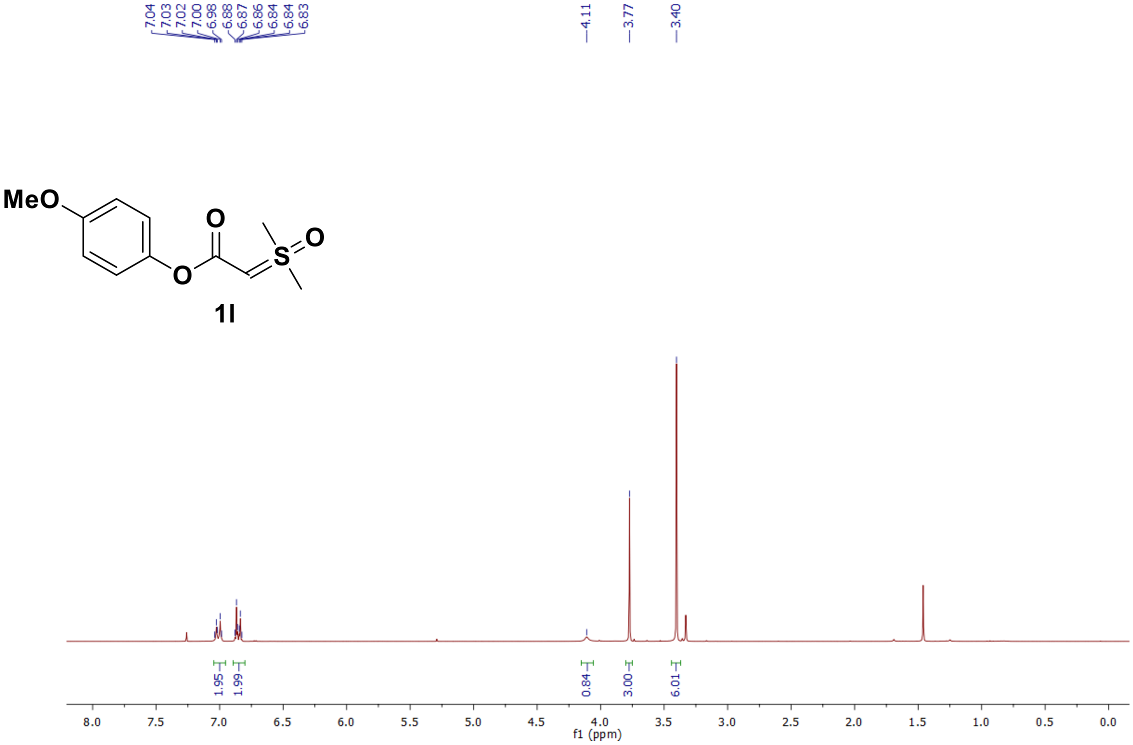

^{1}H NMR (300 MHz, DMSO-*d*$_6$) of compound 2-(dimethyl(oxo)-λ^6-sulfaneylidene)-N-phenylacetamide (**1m**).

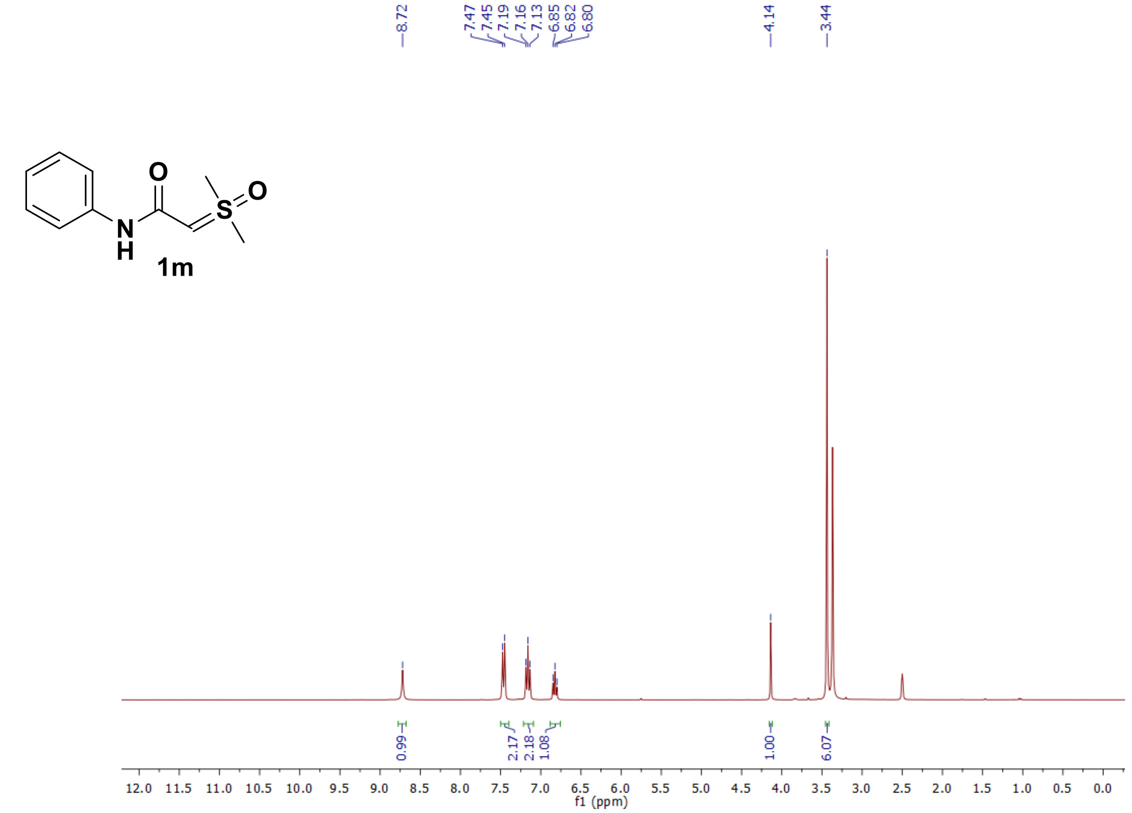

^{1}H NMR (300 MHz, DMSO-*d*$_6$) of compound 2-(dimethyl(oxo)-λ^6-sulfaneylidene)-*N*-(p-tolyl)acetamide (**1n**).

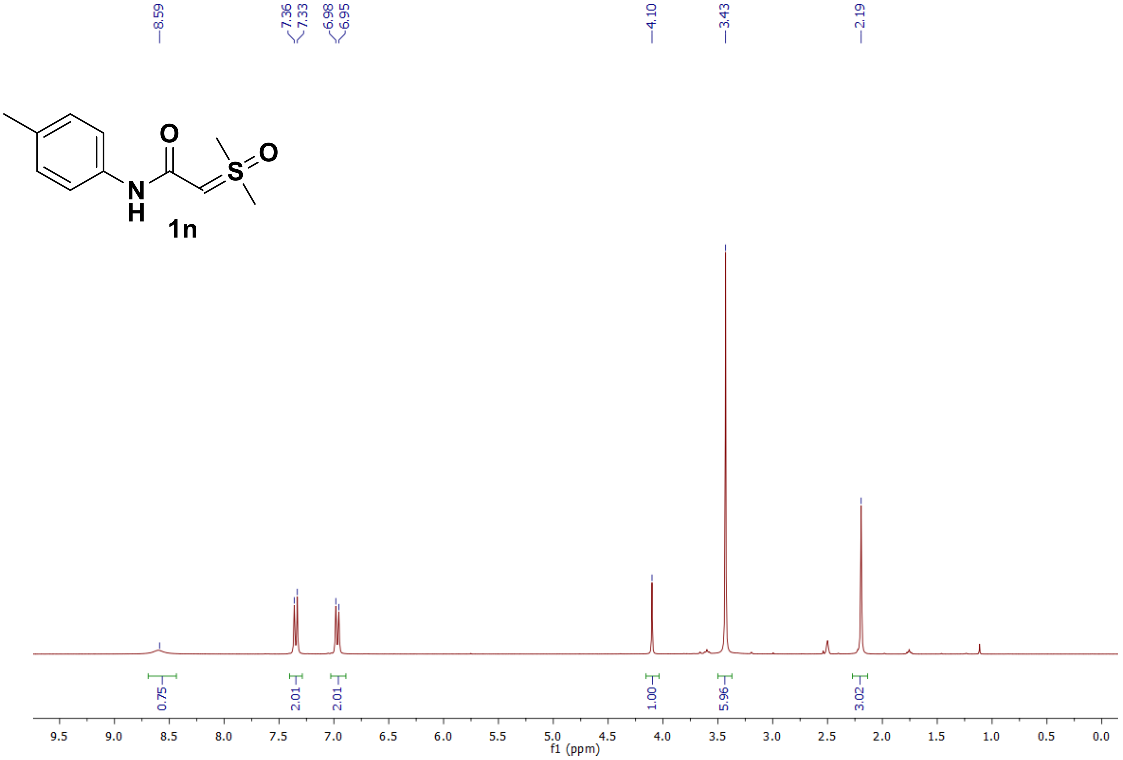

^{13}C NMR (76 MHz, DMSO-*d*$_6$) of compound 2-(dimethyl(oxo)-λ^6-sulfaneylidene)-*N*-(*p*-tolyl)acetamide (**1n**).

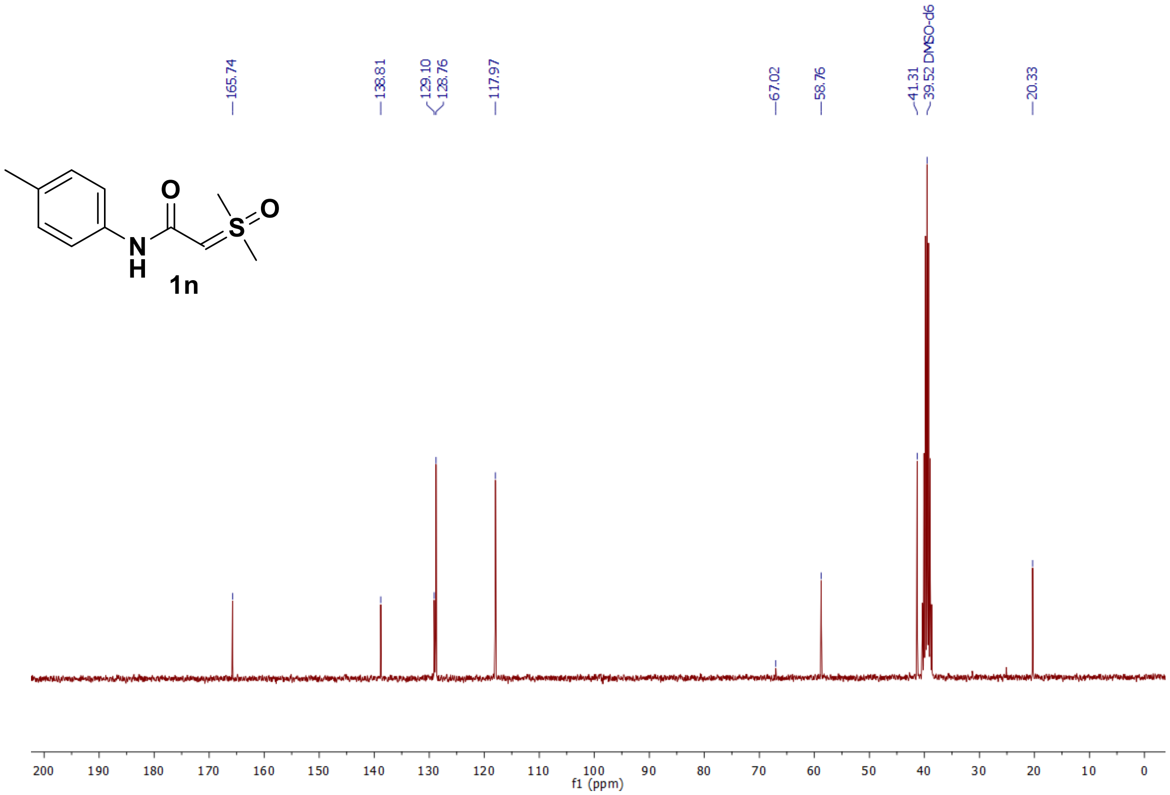

^{1}H NMR (300 MHz, DMSO-*d*$_6$) of compound *N*-(4-chlorophenyl)-2-(dimethyl(oxo)-λ^6-sulfaneylidene)acetamide (**1o**).

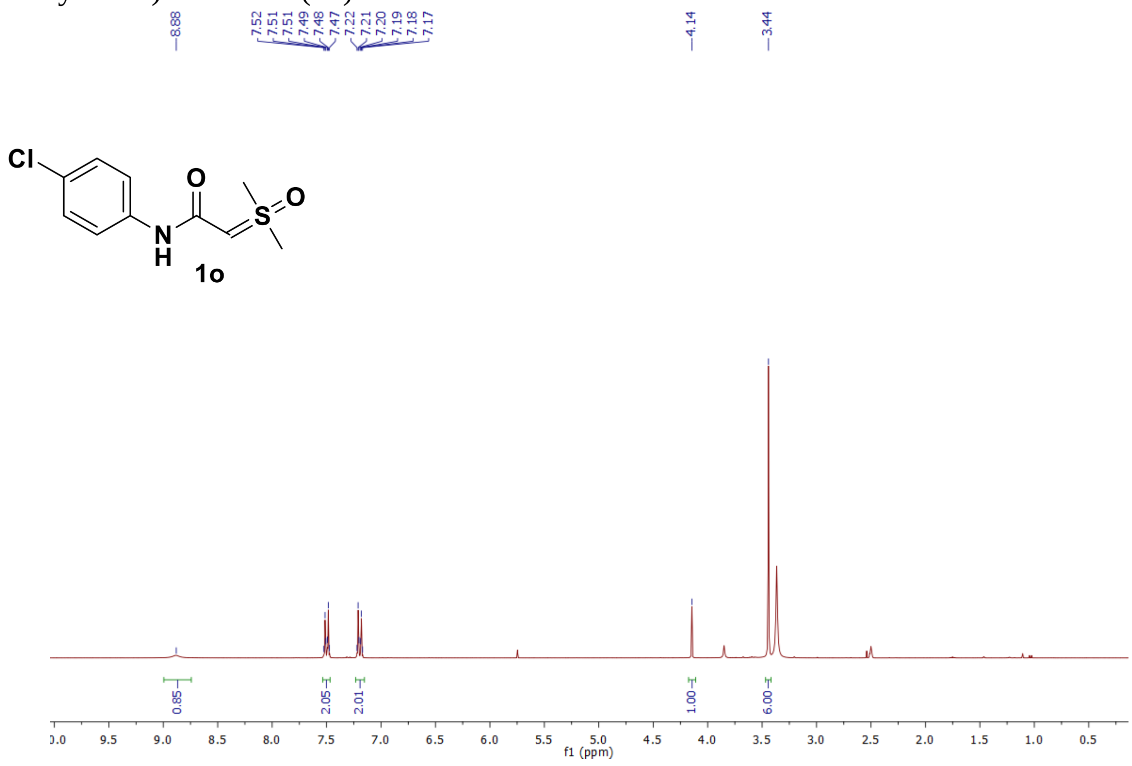

^{13}C NMR (76 MHz, DMSO-*d*$_6$) of compound *N*-(4-chlorophenyl)-2-(dimethyl(oxo)-λ^6-sulfaneylidene)acetamide (**1o**).

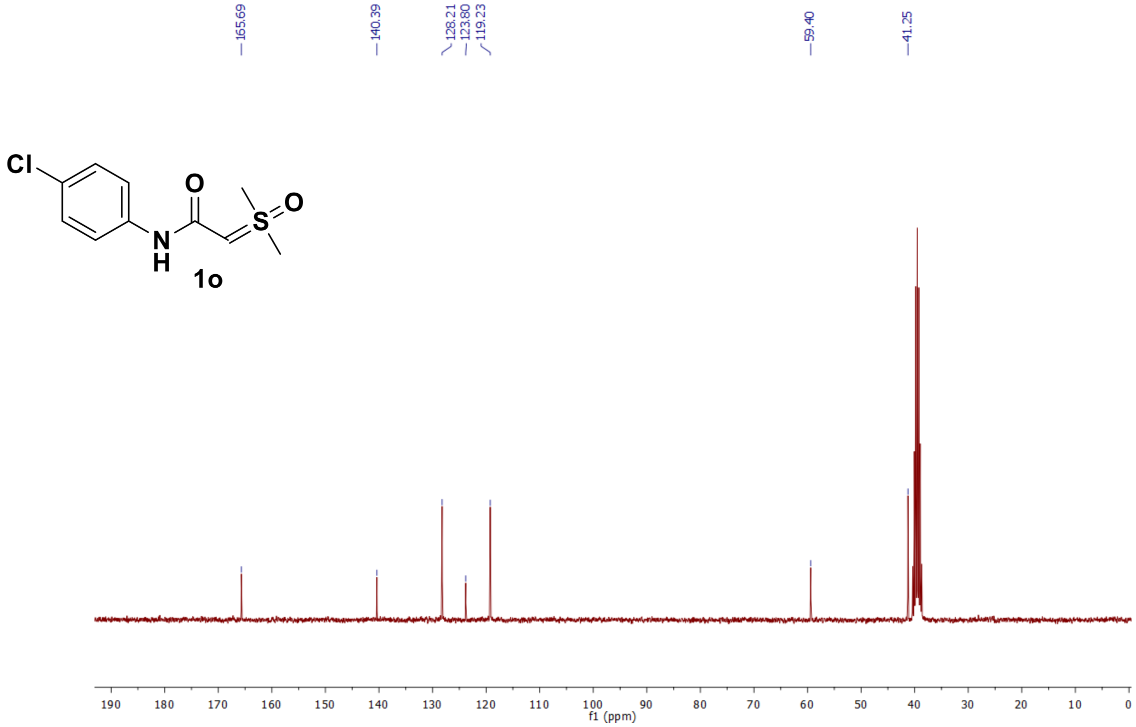

^{1}H NMR (300 MHz, DMSO-*d*$_6$) of compound 2-(dimethyl(oxo)-λ^6-sulfaneylidene)-*N*-(4-methoxyphenyl)acetamide (**1p**).

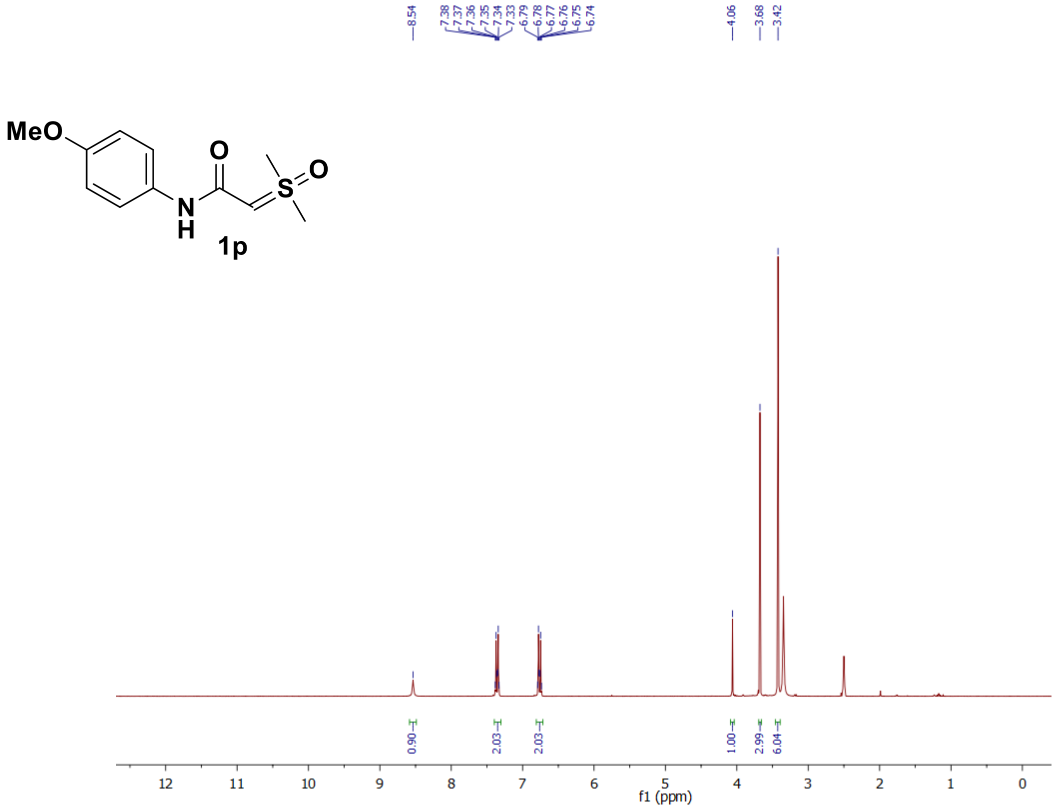

^{13}C NMR (76 MHz, DMSO-*d*$_6$) of compound *N*-(4-chlorophenyl)-2-(dimethyl(oxo)-λ^6-sulfaneylidene)acetamide (**1p**).

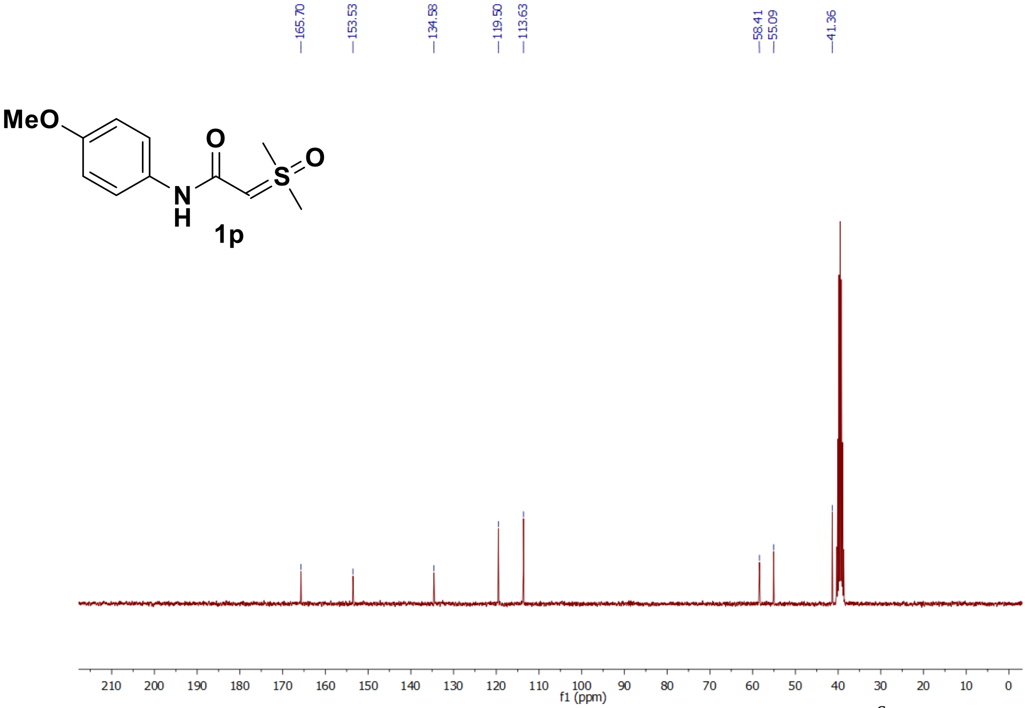

^{1}H NMR (300 MHz, DMSO-*d*$_6$) of compound 2-(dimethyl(oxo)-λ^6-sulfaneylidene)-*N*-(4-(trifluoromethyl)phenyl)acetamide (**1q**).

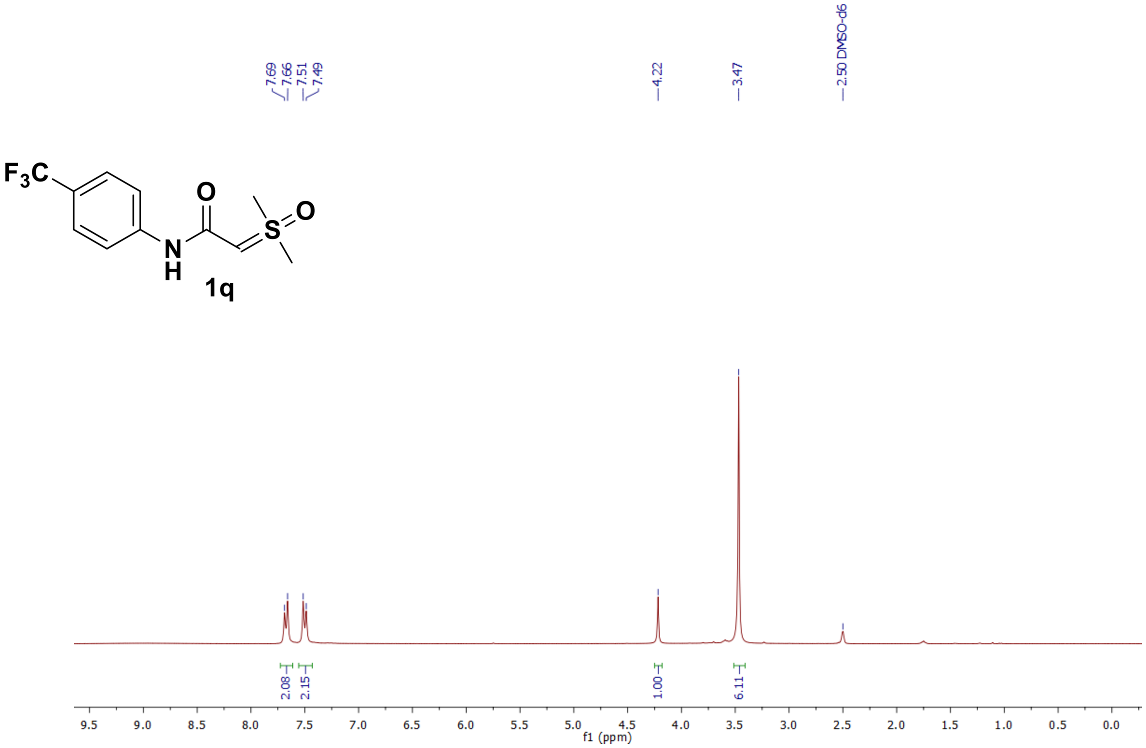

^{13}C NMR (76 MHz, DMSO-*d*6) of compound 2-(dimethyl(oxo)-λ6-sulfaneylidene)-*N*-(4-(trifluoromethyl)phenyl)acetamide (**1q**).

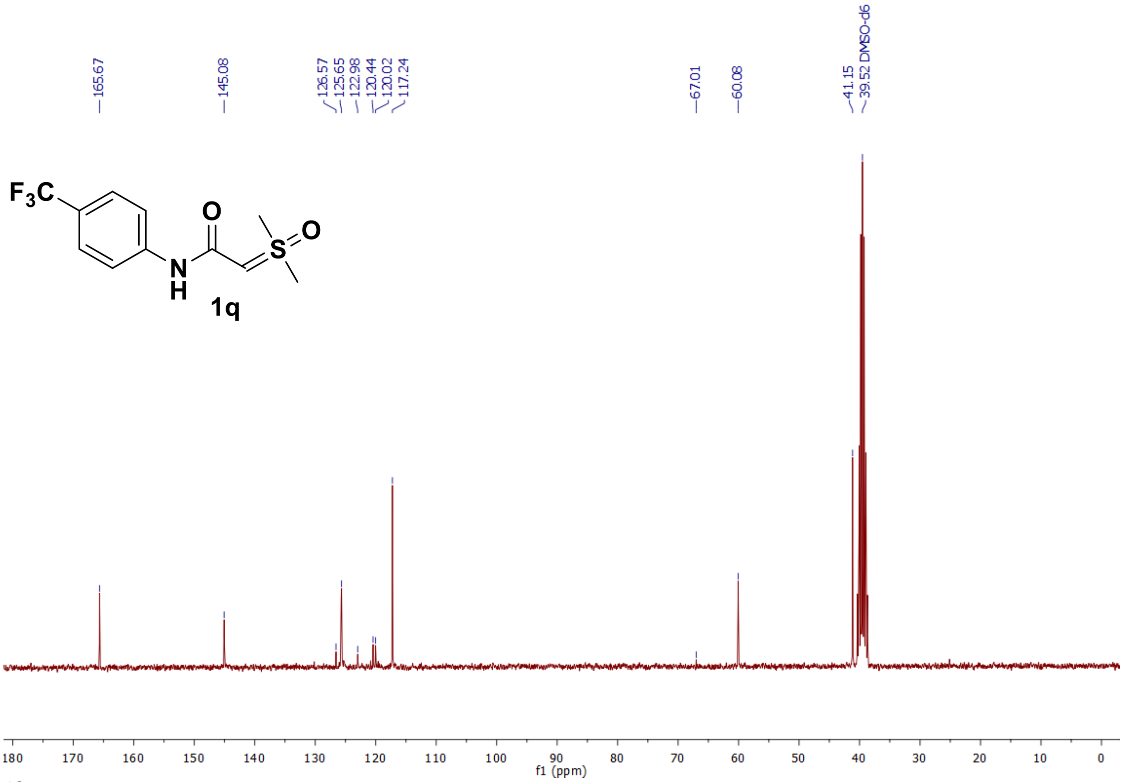

^{19}F NMR (282 MHz, CDCl3) of compound 2-(dimethyl(oxo)-λ6-sulfaneylidene)-*N*-(4-(trifluoromethyl)phenyl)acetamide (**1q**).

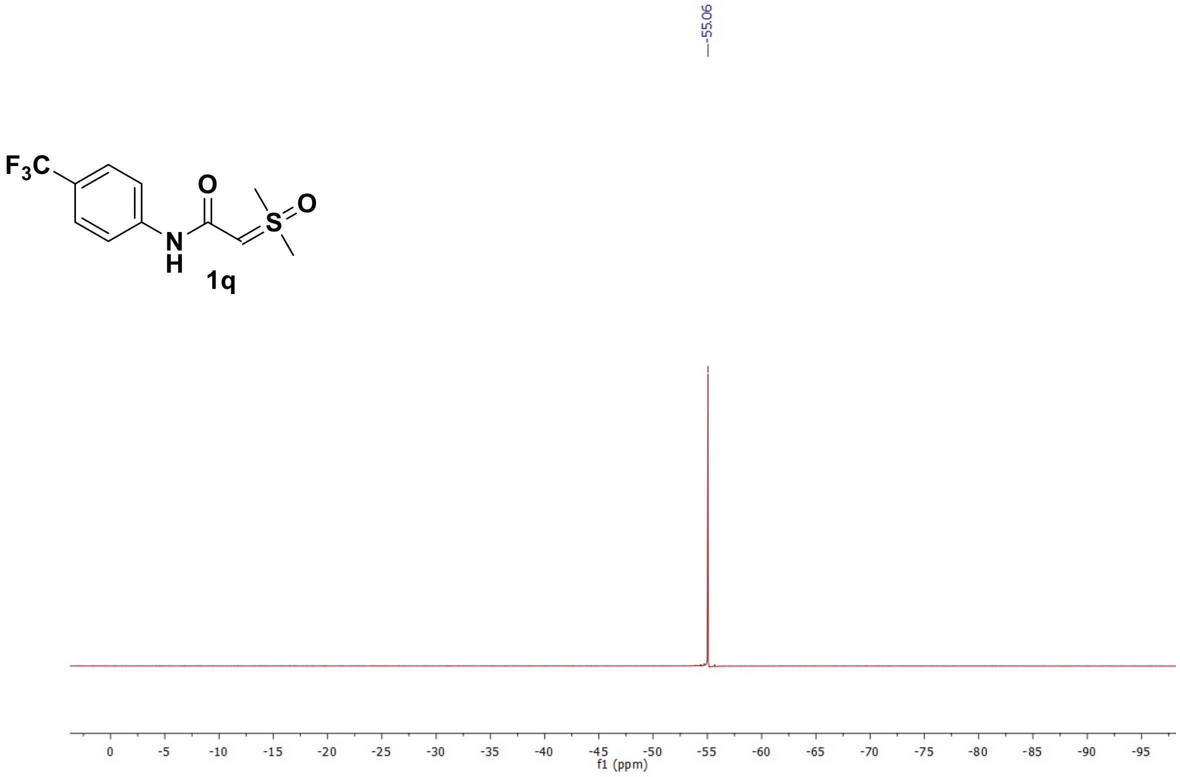

^{1}H NMR (300 MHz, $CDCl_3$) of compound **3a**.

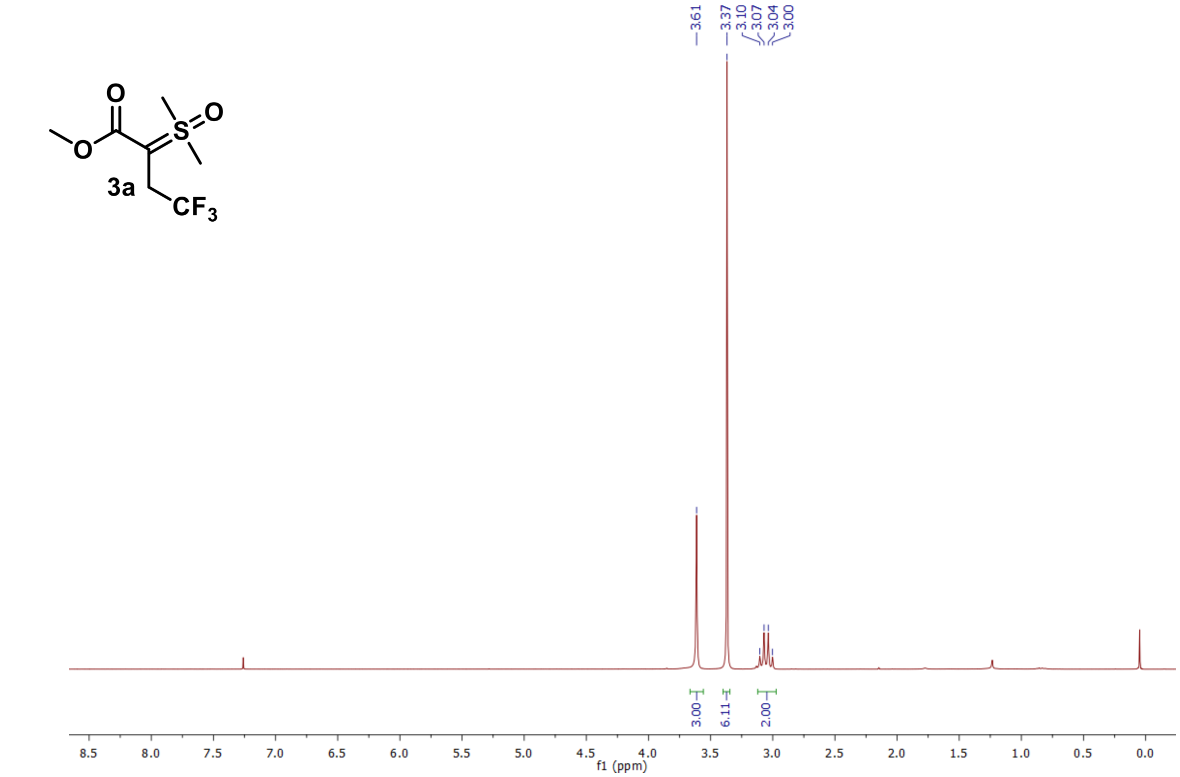

^{13}C NMR (126 MHz, $CDCl_3$) of compound **3a.**

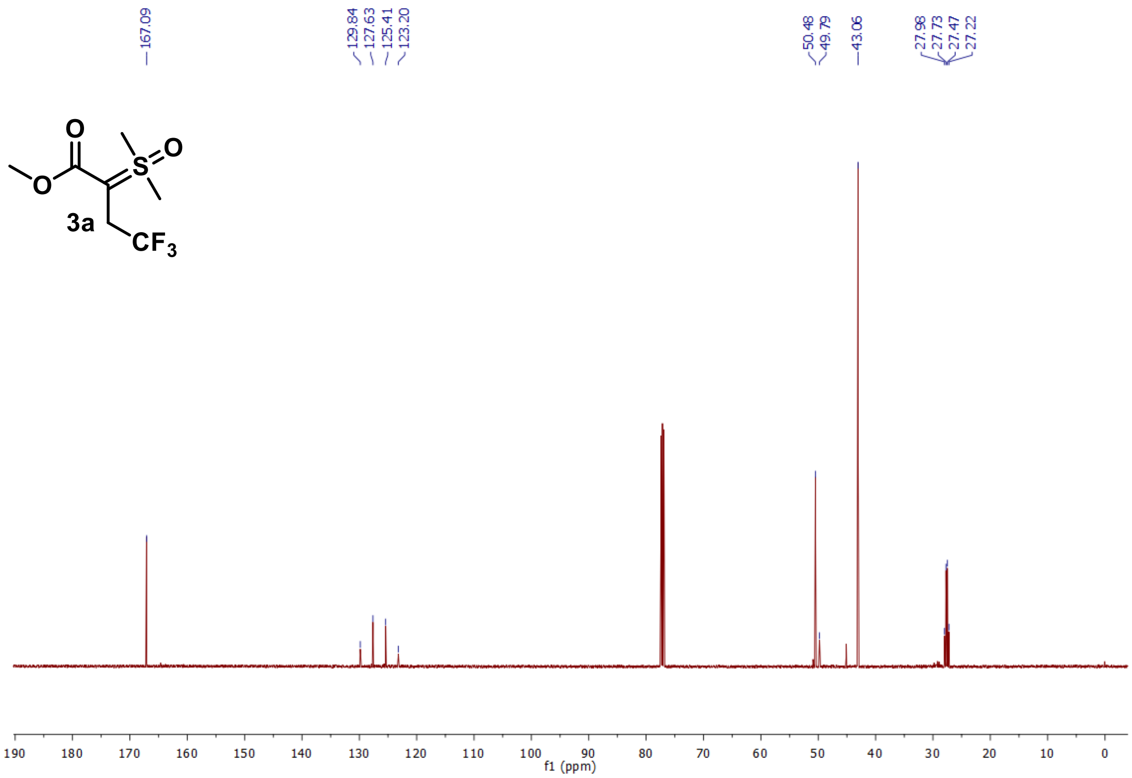

HMBC spectra in $CDCl_3$ of compound **3a.**

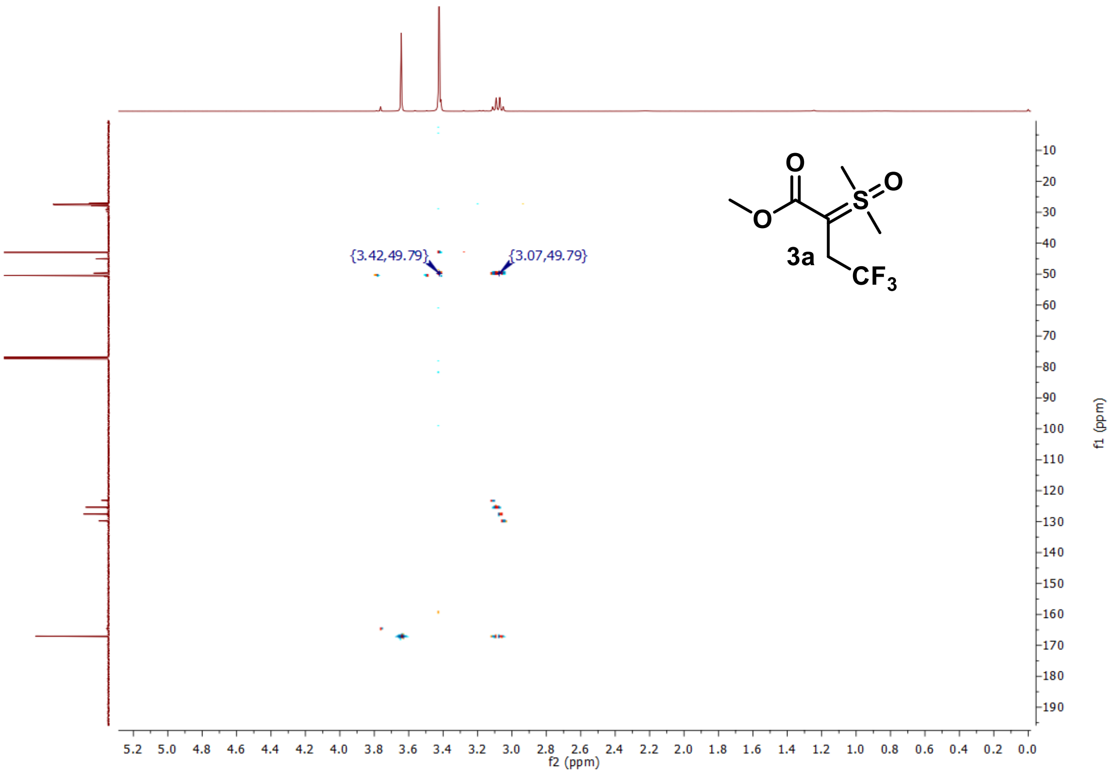

^{19}F NMR (282 MHz, $CDCl_3$) of compound **3a.**

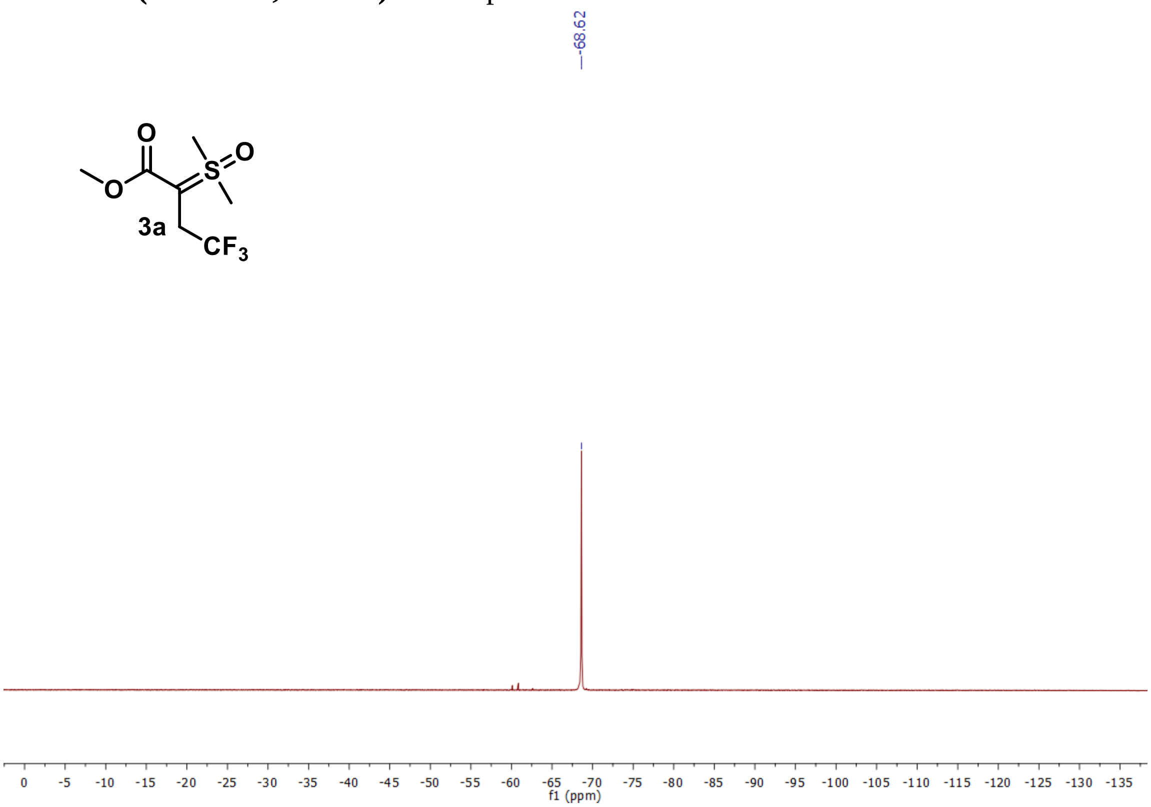

^{1}H NMR (300 MHz, CDCl$_3$) of compound **3b**.

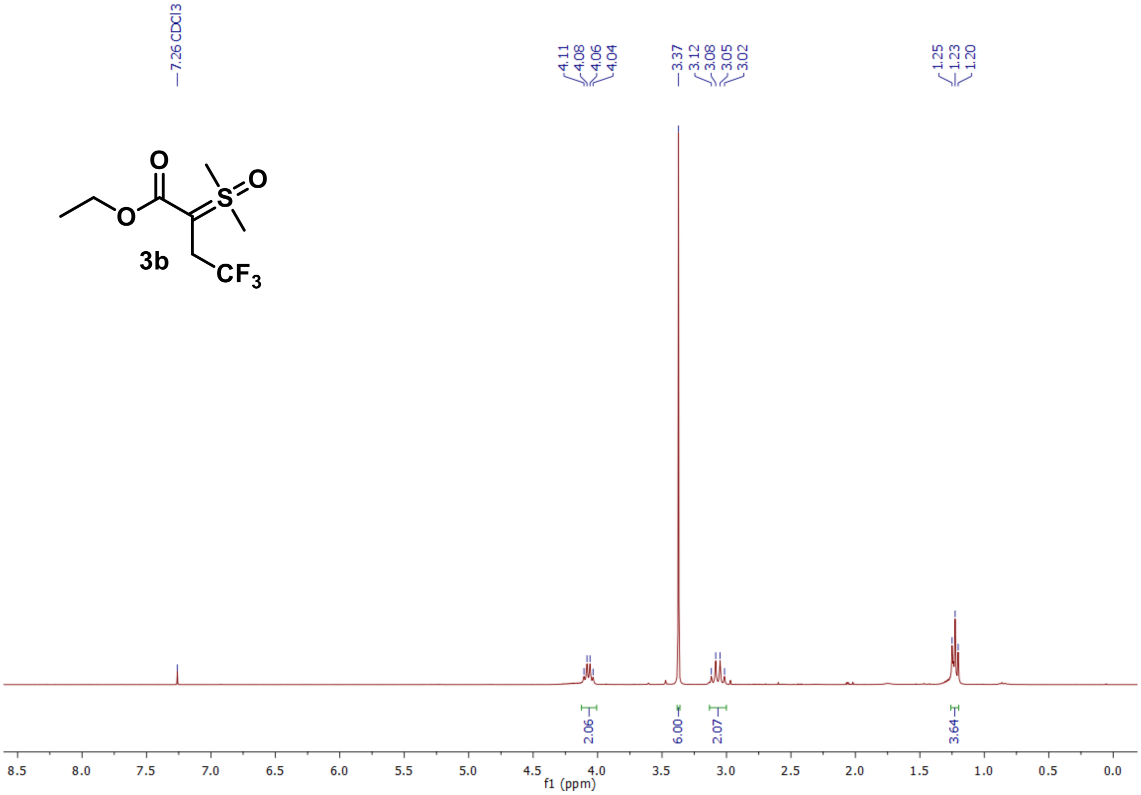

^{13}C{^{19}F}(151 MHz, CDCl$_3$) of compound **3b.**

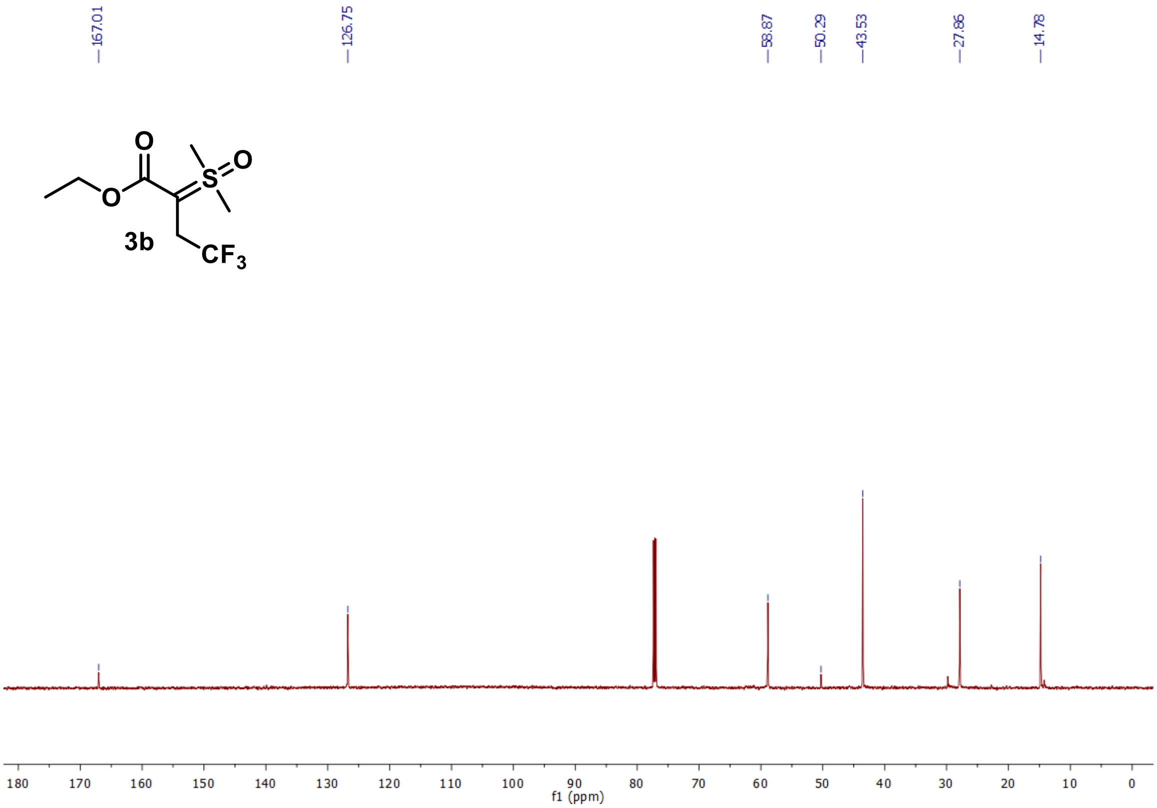

^{19}F NMR (282 MHz, CDCl3) of compound **3b.**

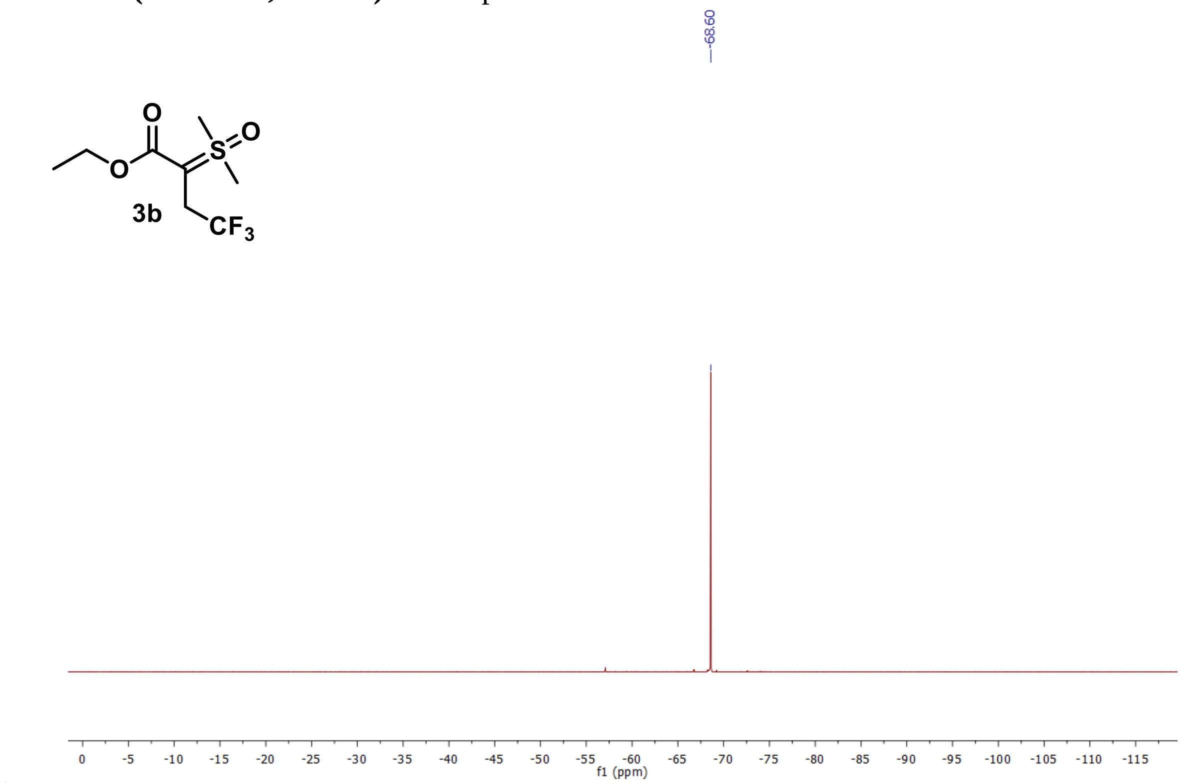

^{1}H NMR (300 MHz, CDCl3) of compound **3c**.

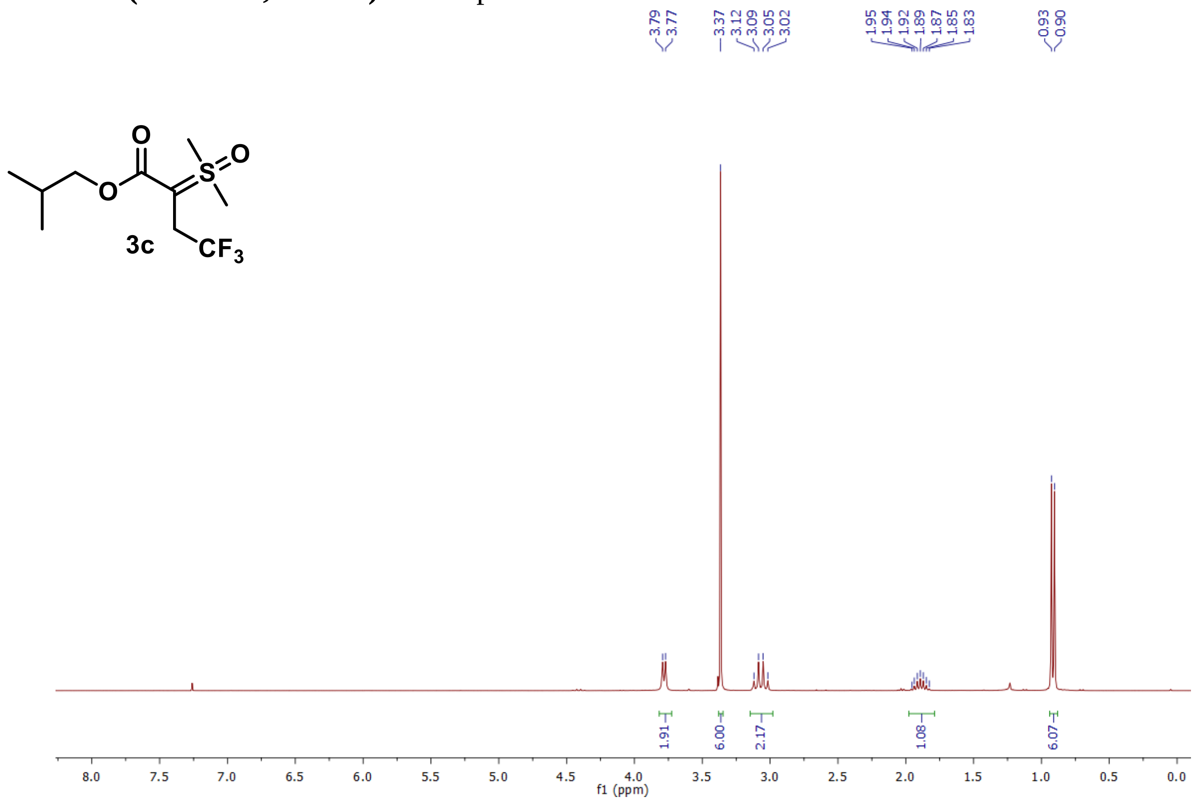

$^{13}C\{^{19}F\}$(151 MHz, $CDCl_3$) of compound **3c.**

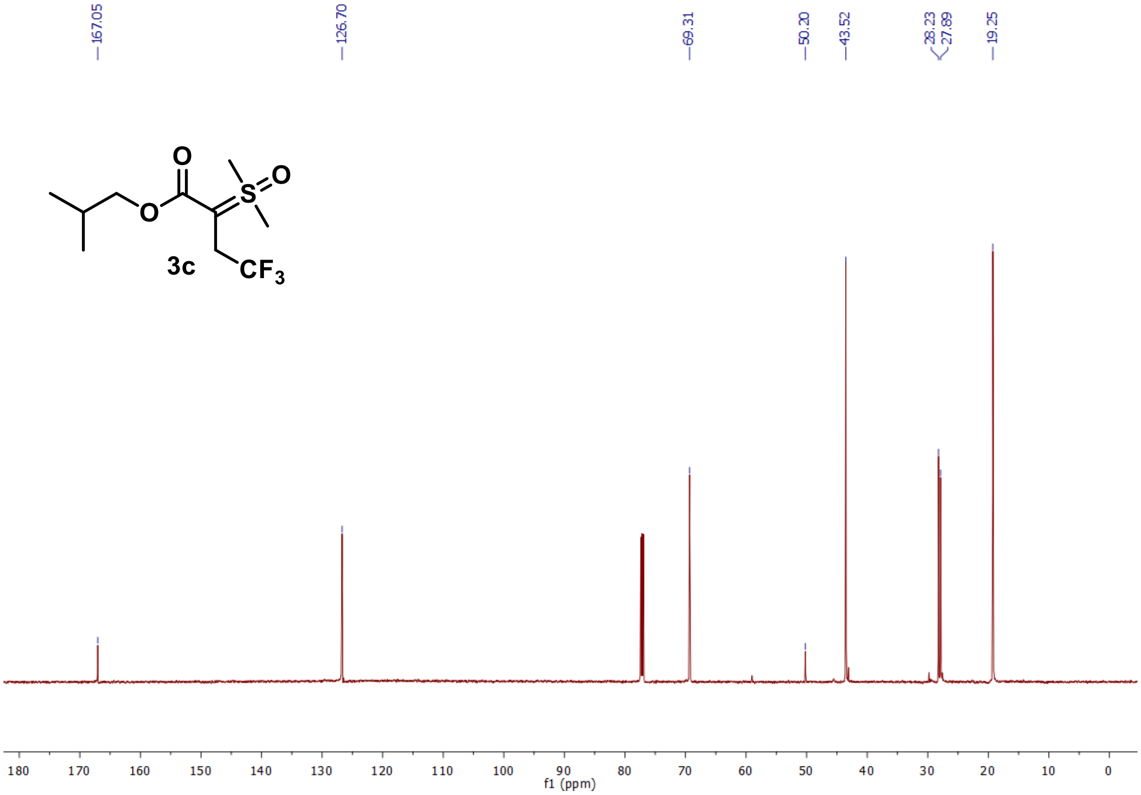

^{19}F NMR (282 MHz, $CDCl_3$) of compound **3c.**

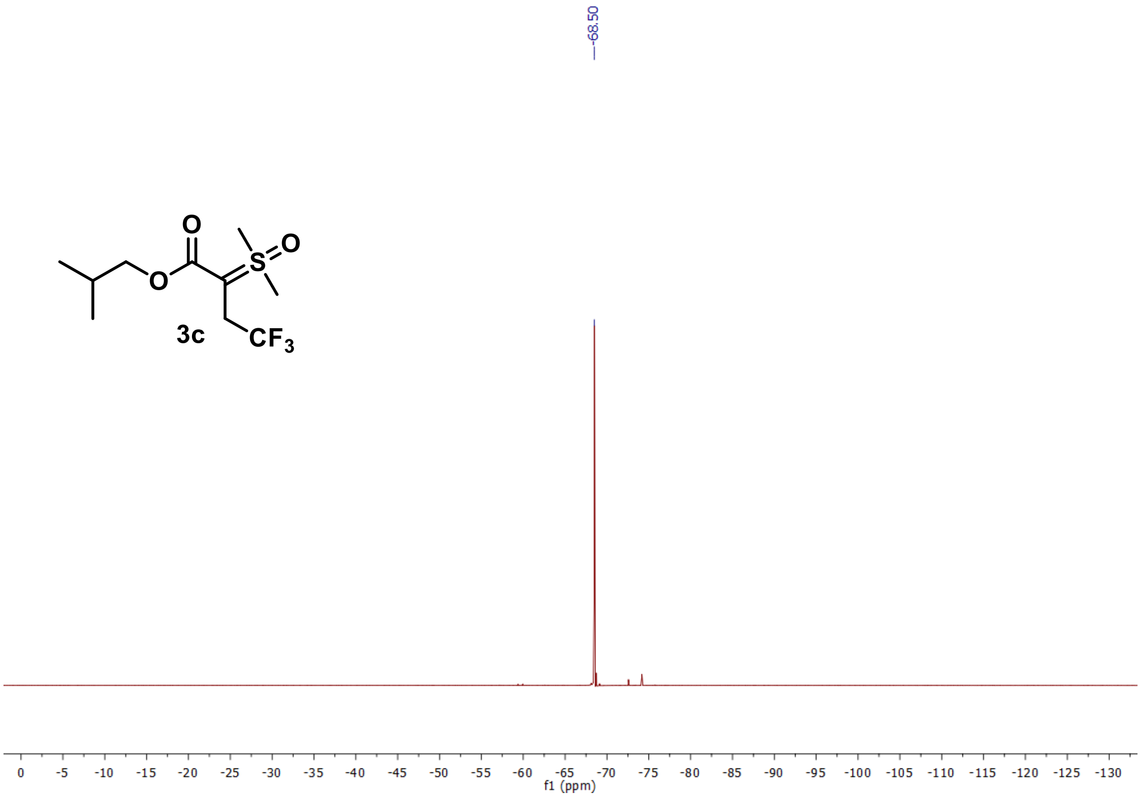

^{1}H NMR (300 MHz, CDCl$_3$) of compound **3d**.

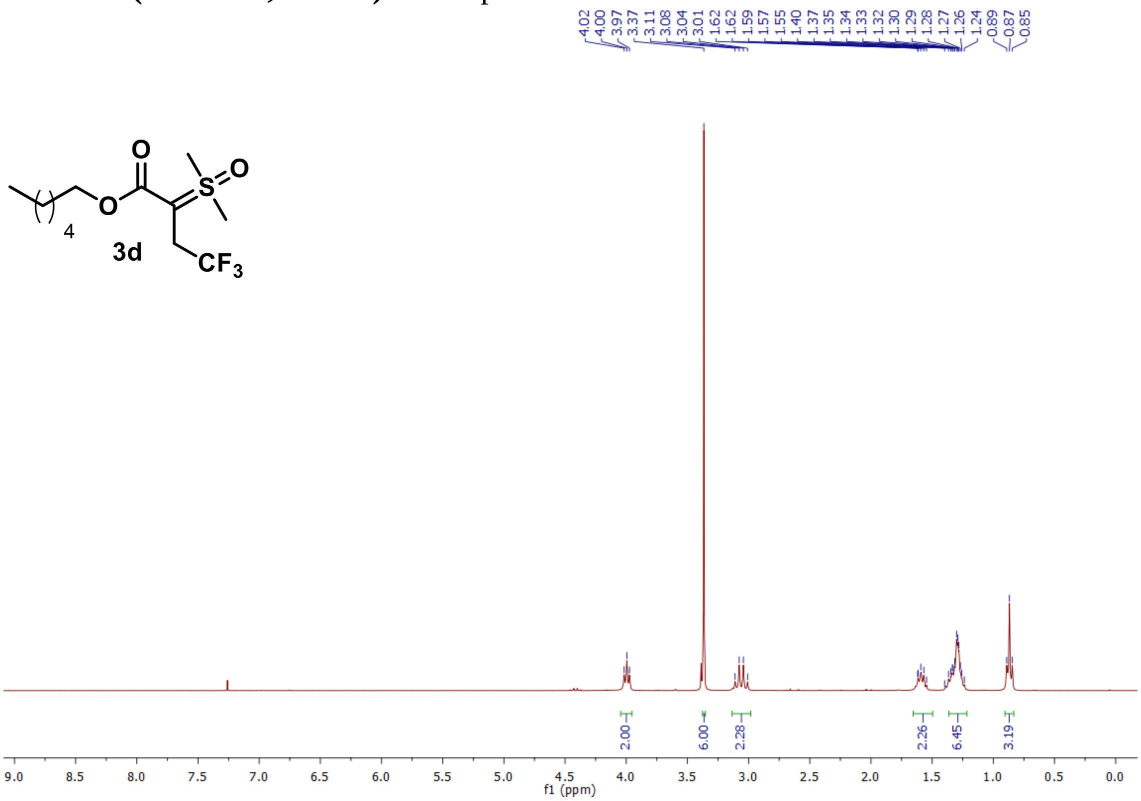

^{13}C{^{19}F}(151 MHz, CDCl$_3$) of compound **3d.**

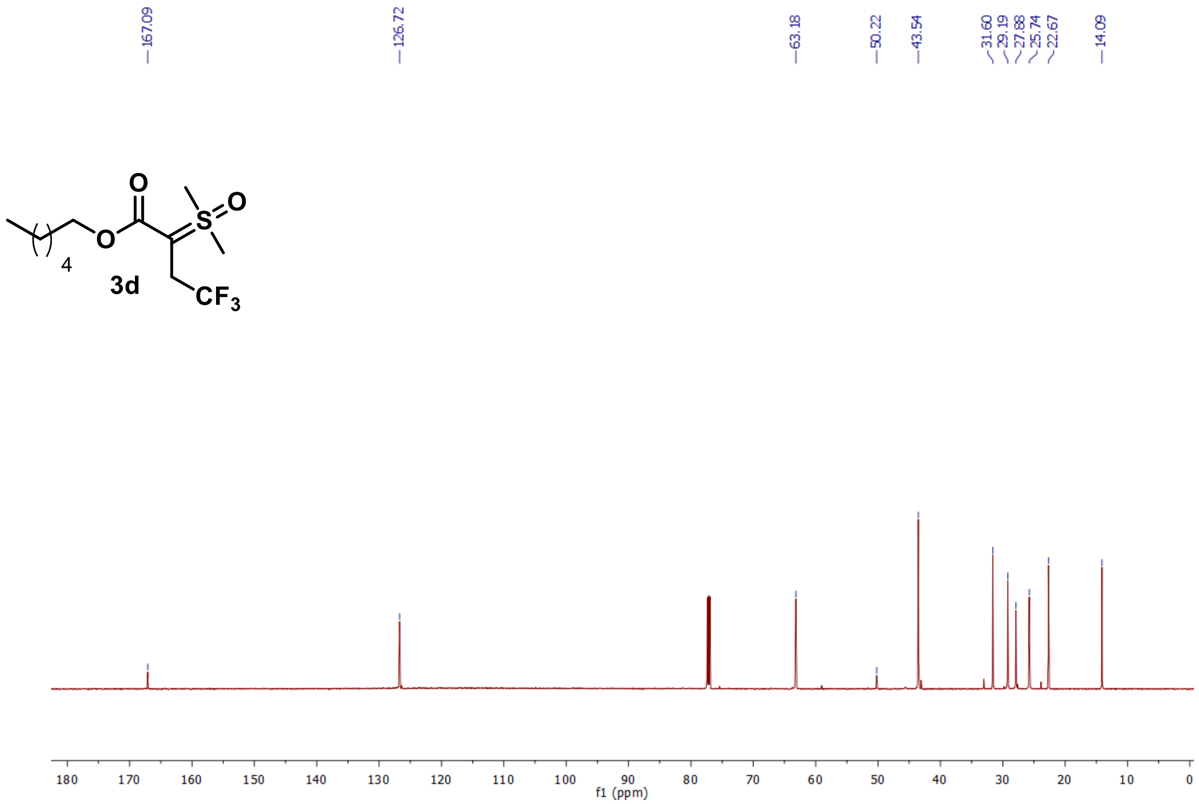

^{19}F NMR (282 MHz, CDCl$_3$) of compound **3d.**

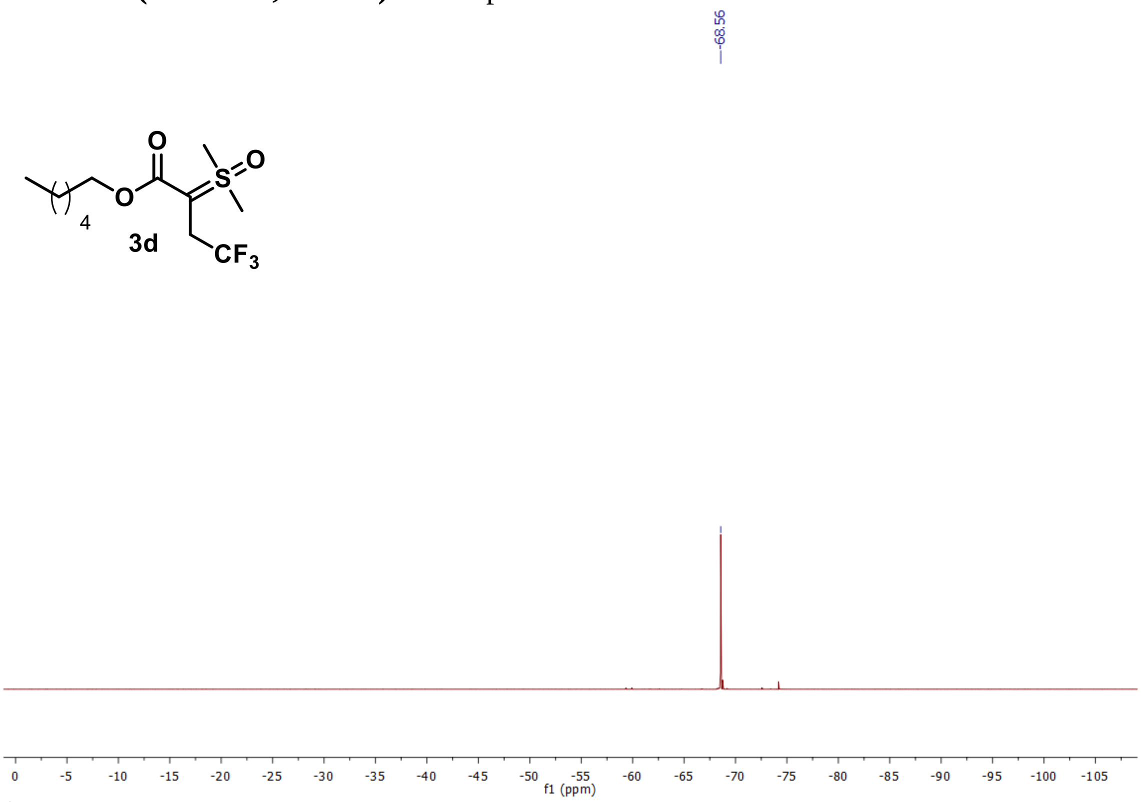

^{1}H NMR (300 MHz, CDCl$_3$) of compound **3e**.

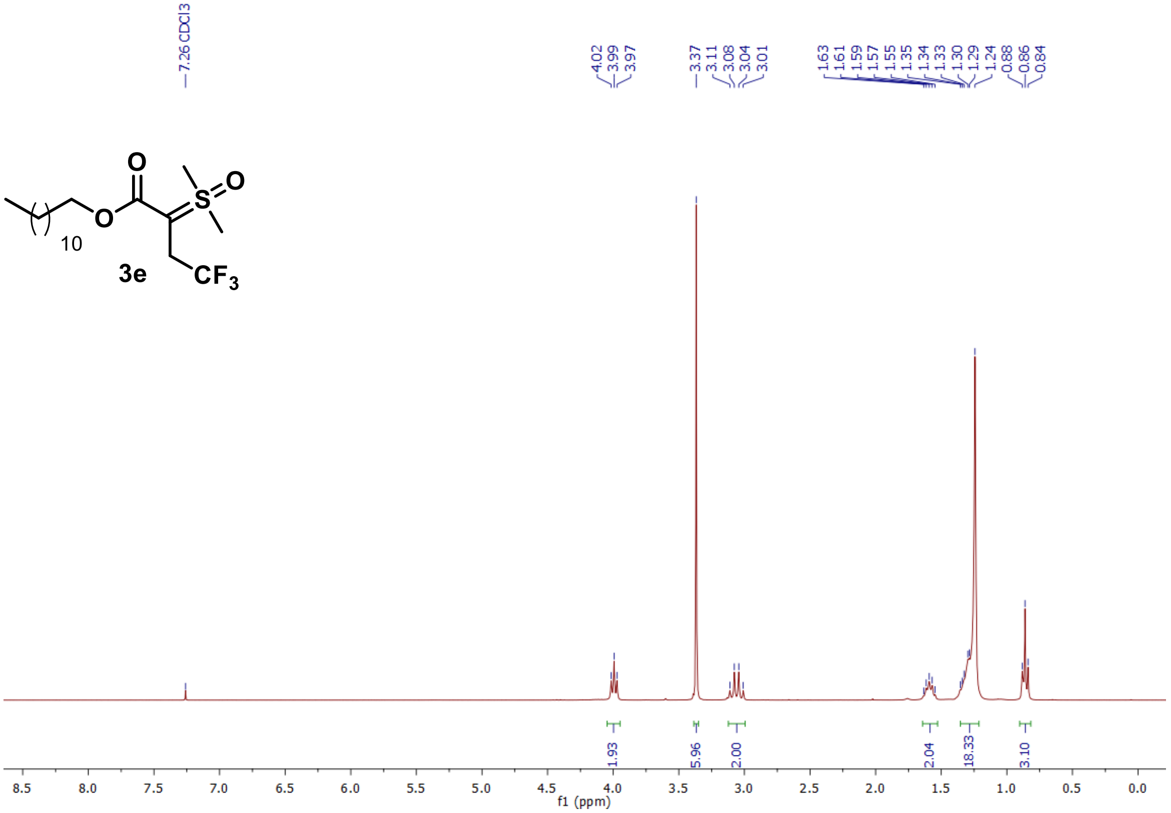

^{13}C{^{19}F}(151 MHz, CDCl$_3$) of compound **3e.**

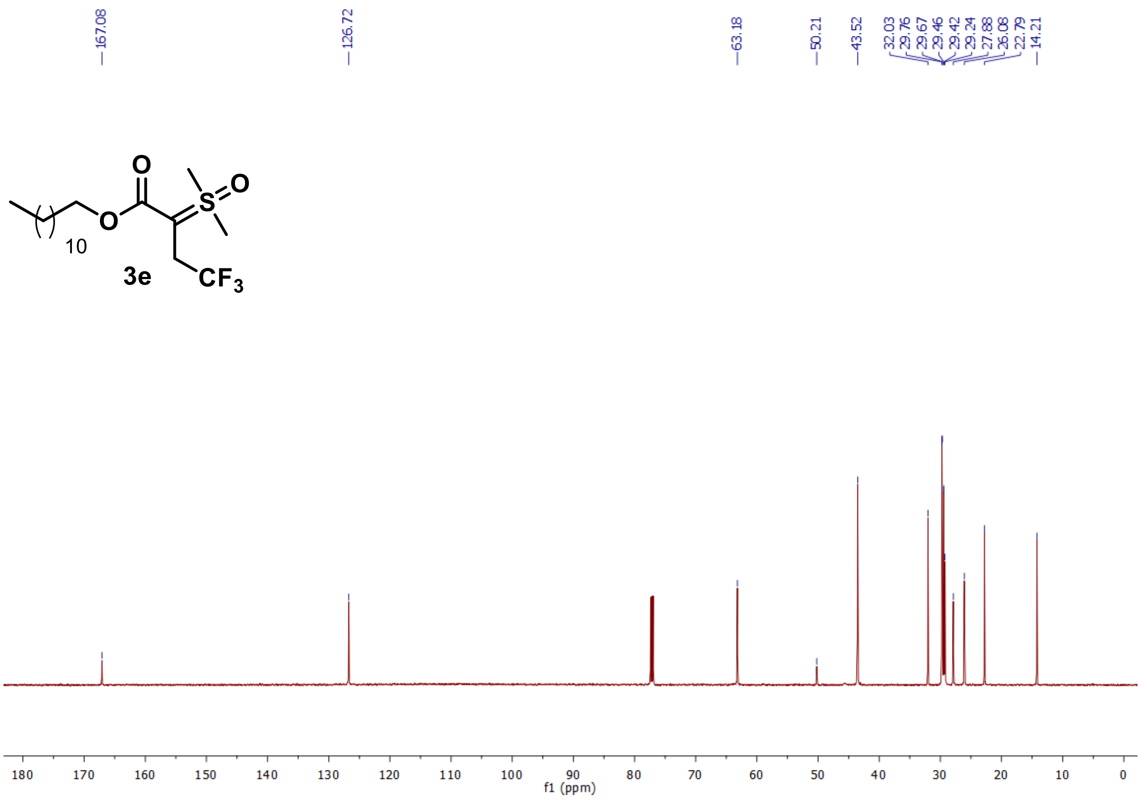

^{19}F NMR (282 MHz, CDCl$_3$) of compound **3e.**

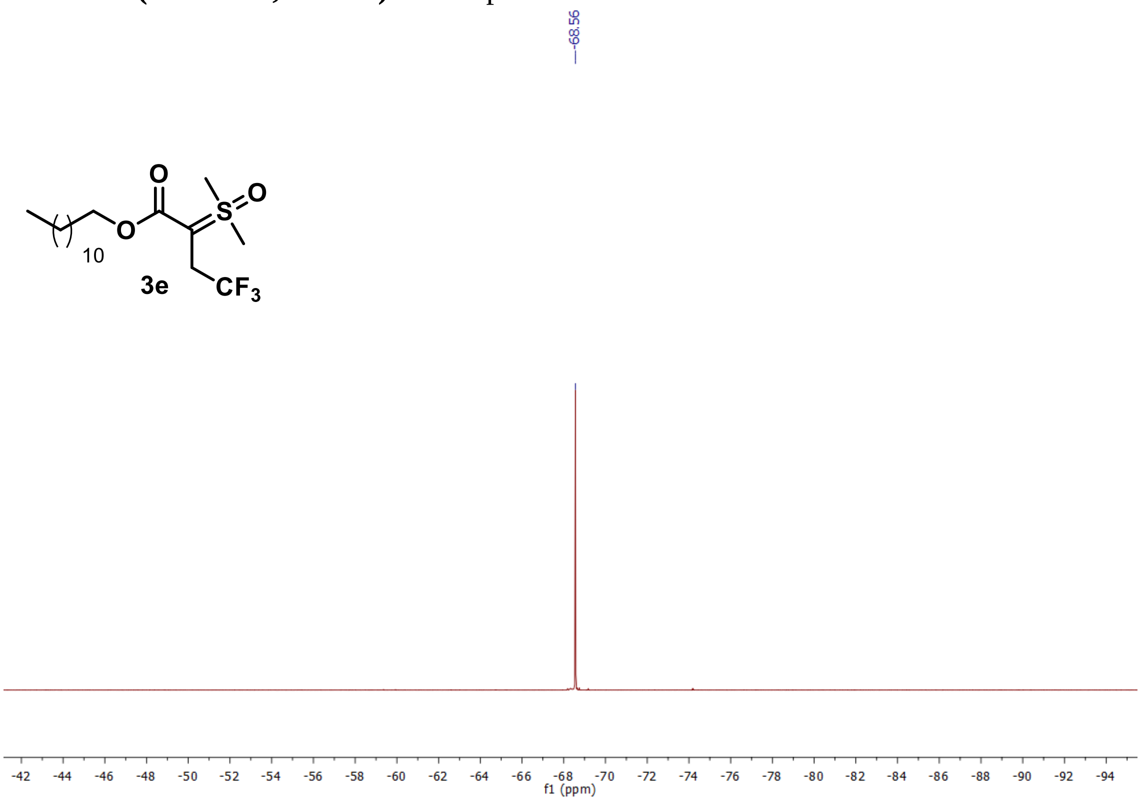

^{1}H NMR (300 MHz, $CDCl_3$) of compound **3f**.

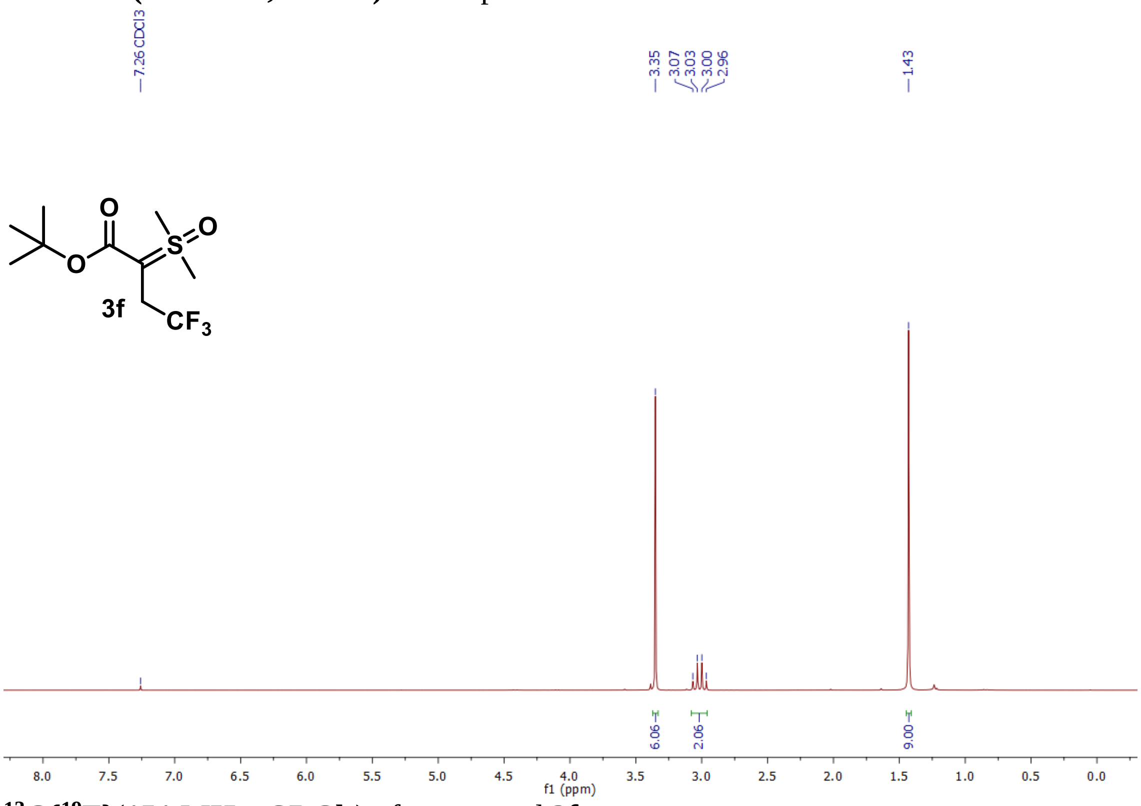

^{13}C{^{19}F}(151 MHz, $CDCl_3$) of compound **3f.**

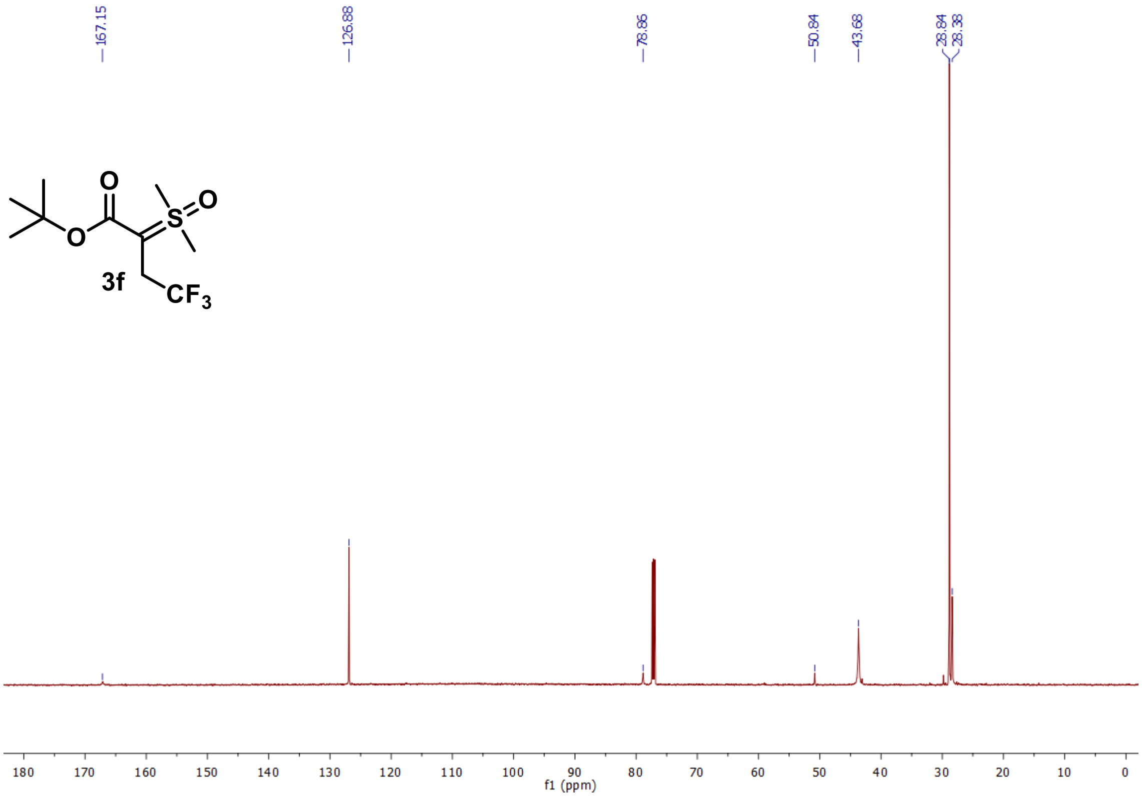

^{19}F NMR (282 MHz, $CDCl_3$) of compound **3f.**

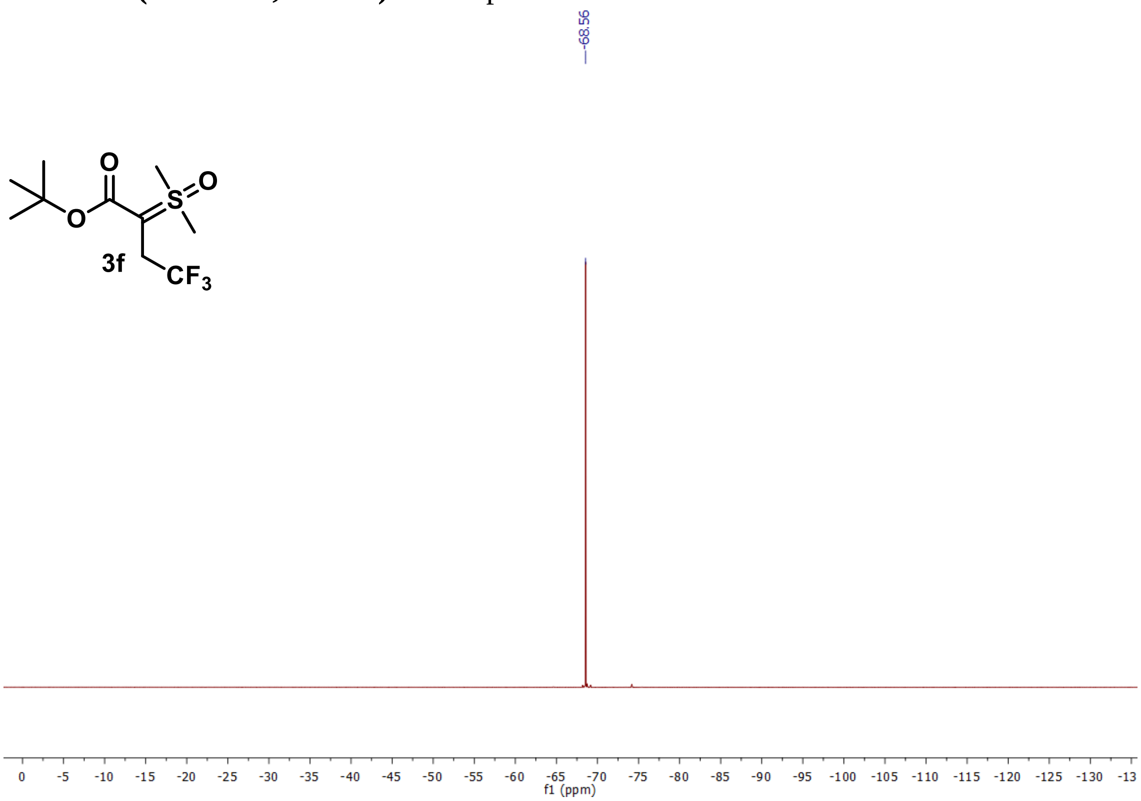

^{1}H NMR (300 MHz, $CDCl_3$) of compound **3g.**

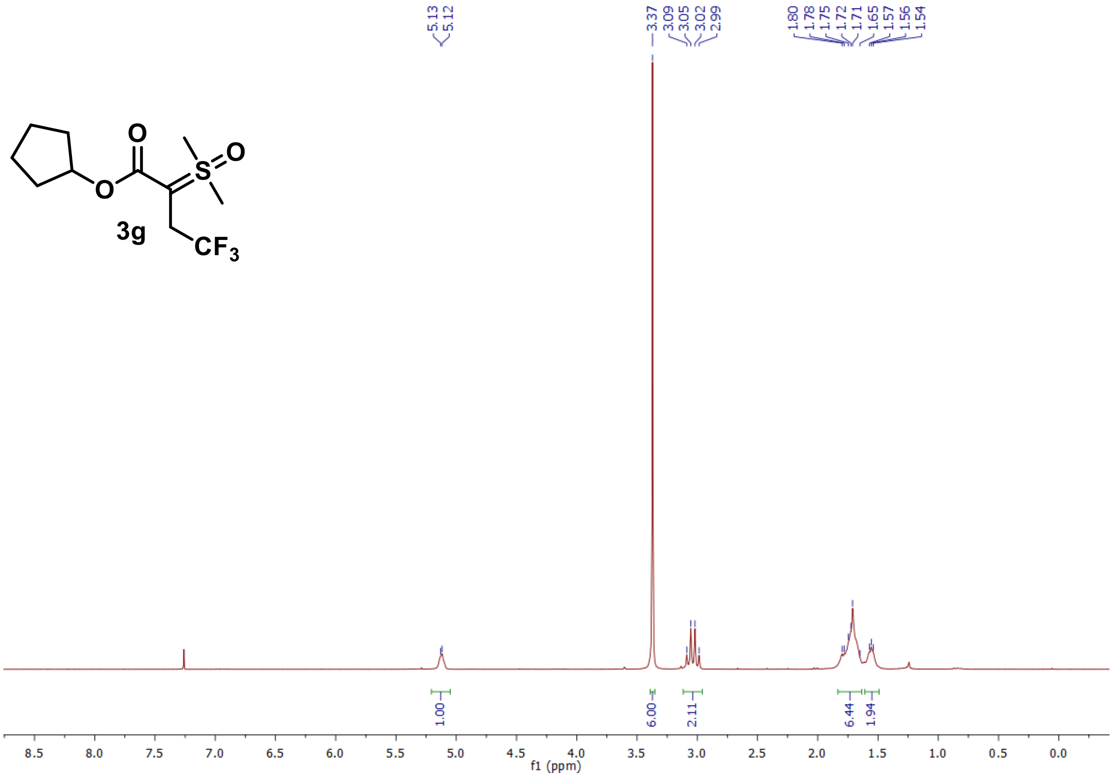

$^{13}C\{^{19}F\}$(151 MHz, $CDCl_3$) of compound **3g.**

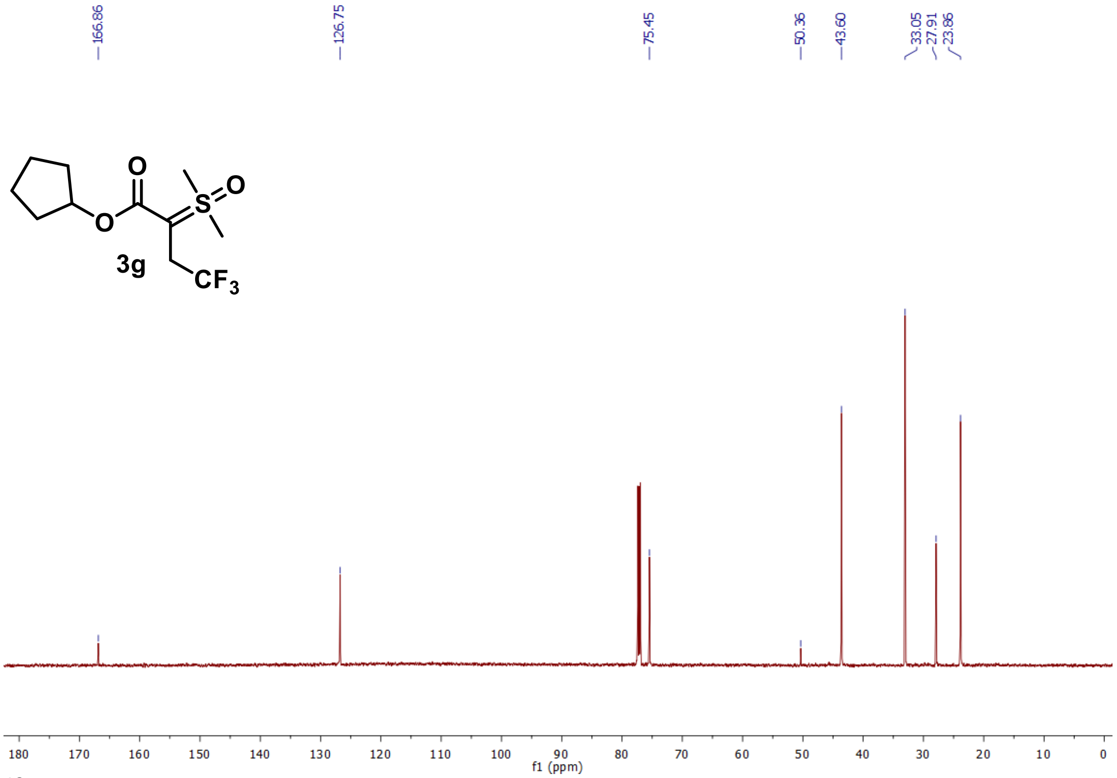

^{19}F NMR (282 MHz, $CDCl_3$) of compound **3g.**

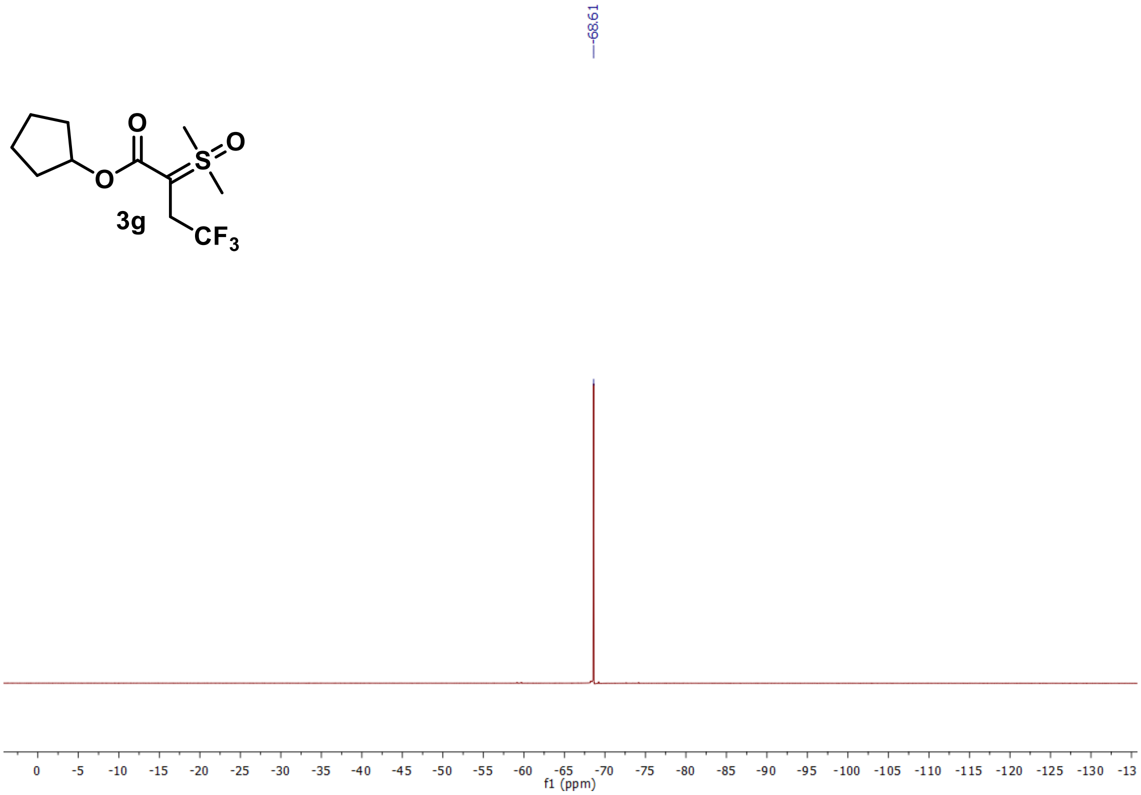

^{1}H NMR (300 MHz, CDCl$_3$) of compound **3h.**

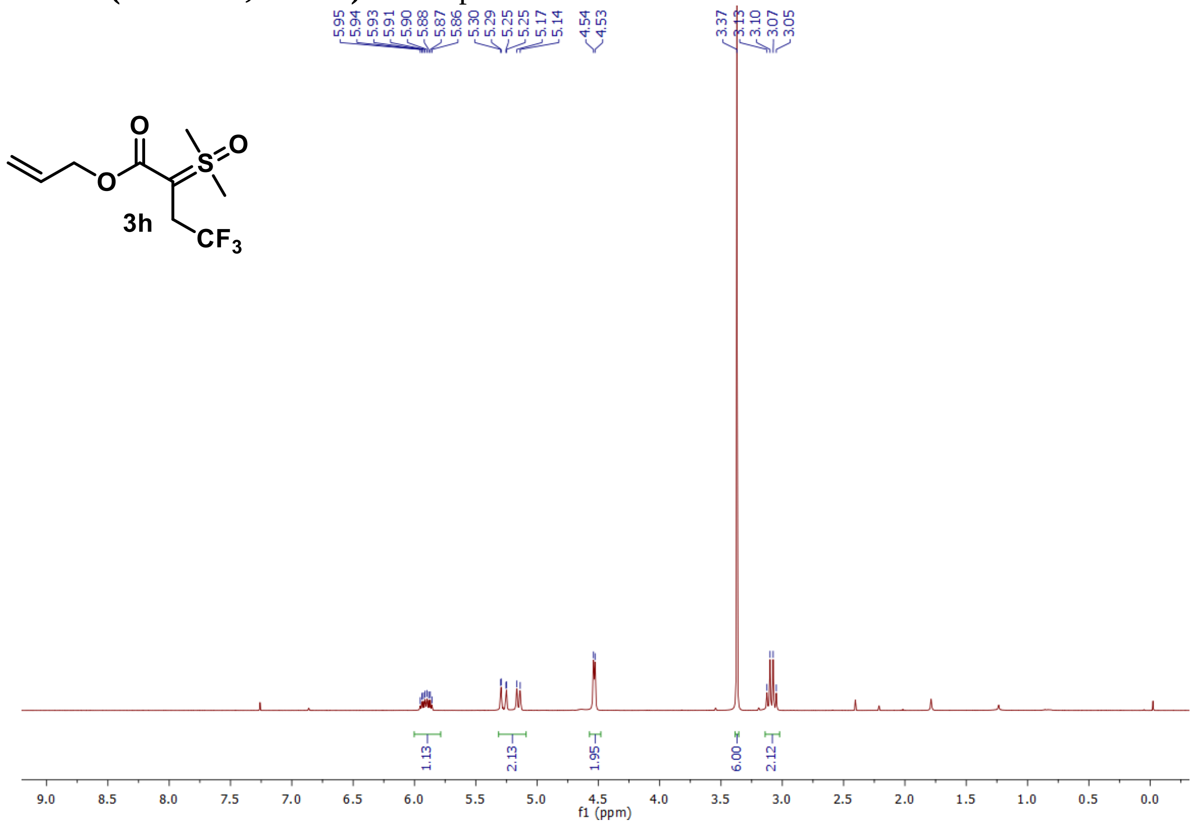

^{13}C (126 MHz, CDCl$_3$) of compound **3h.**

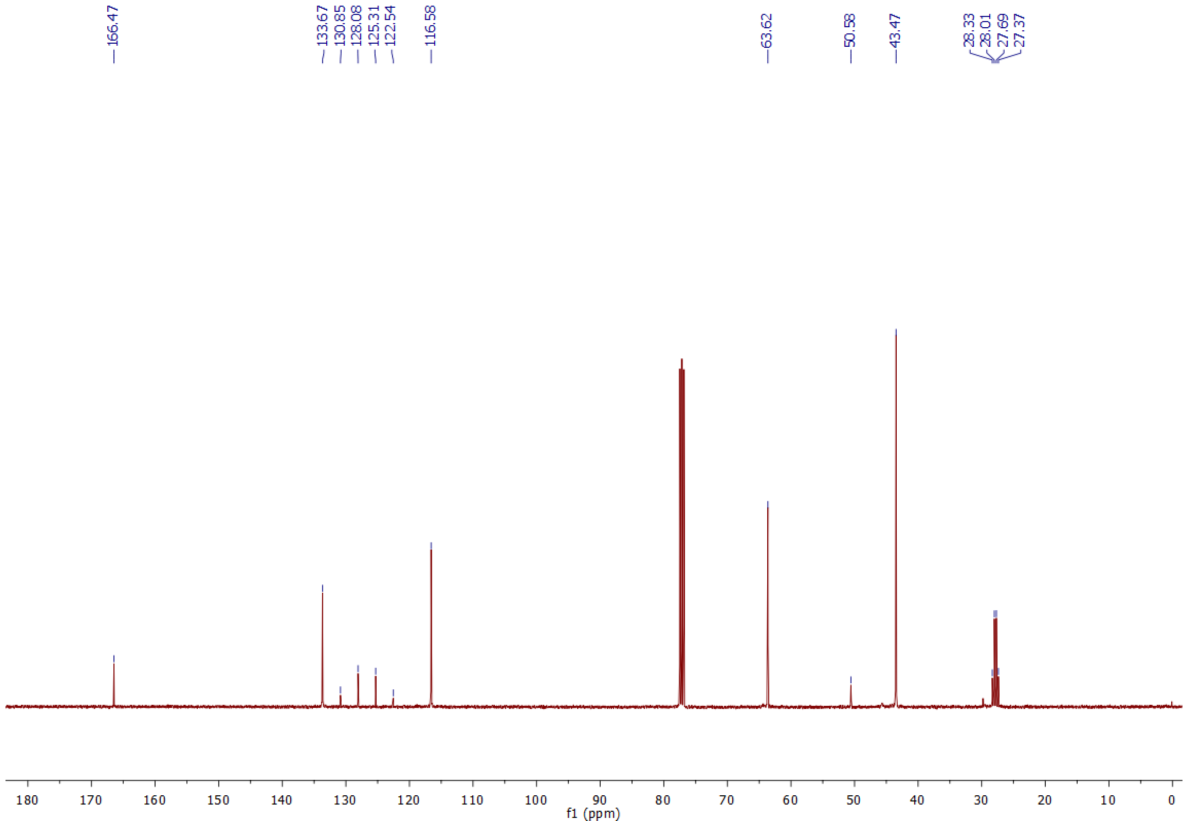

^{19}F NMR (282 MHz, $CDCl_3$) of compound **3h.**

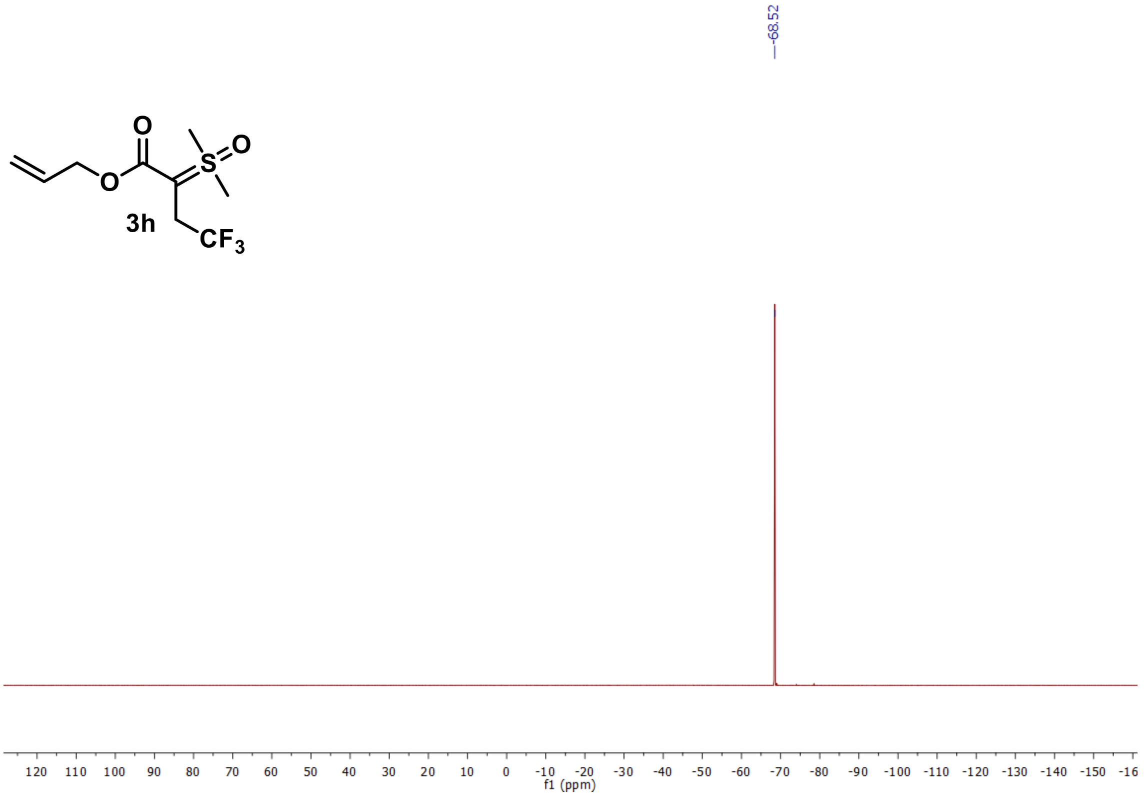

^{1}H NMR (300 MHz, $CDCl_3$) of compound **3i.**

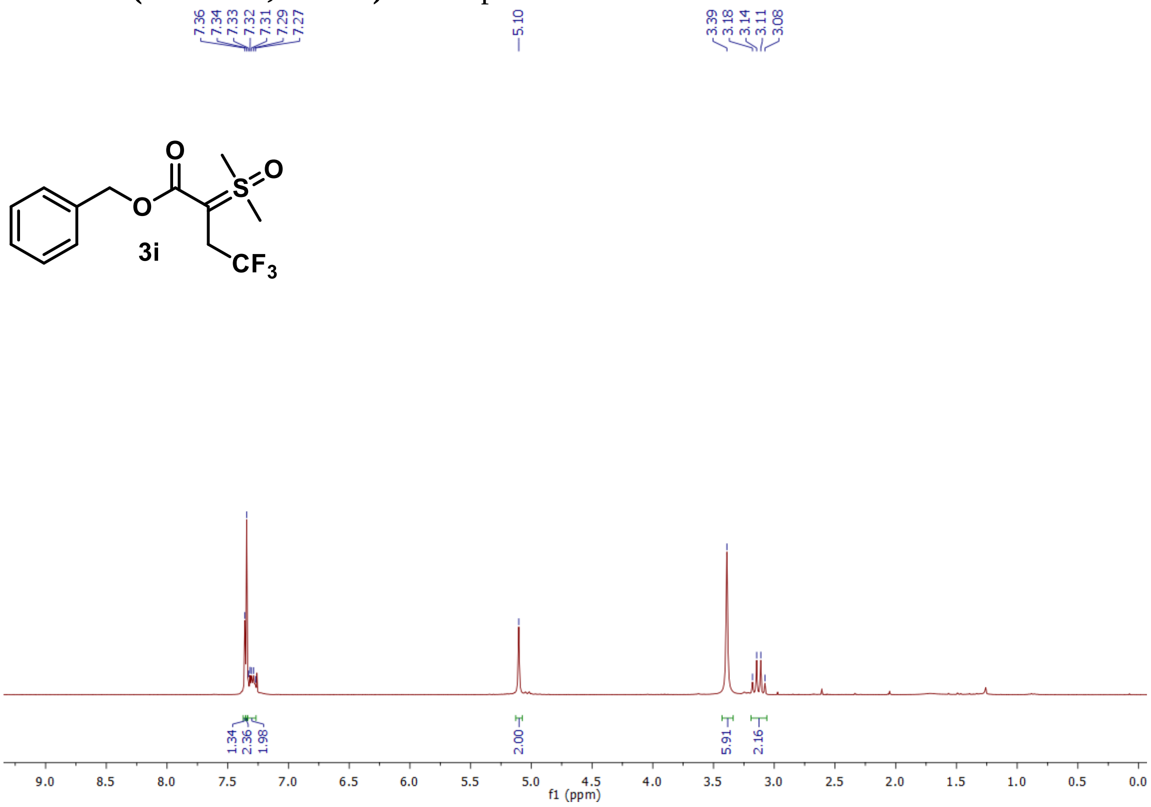

$^{13}C\{^{19}F\}$(151 MHz, $CDCl_3$) of compound **3i.**

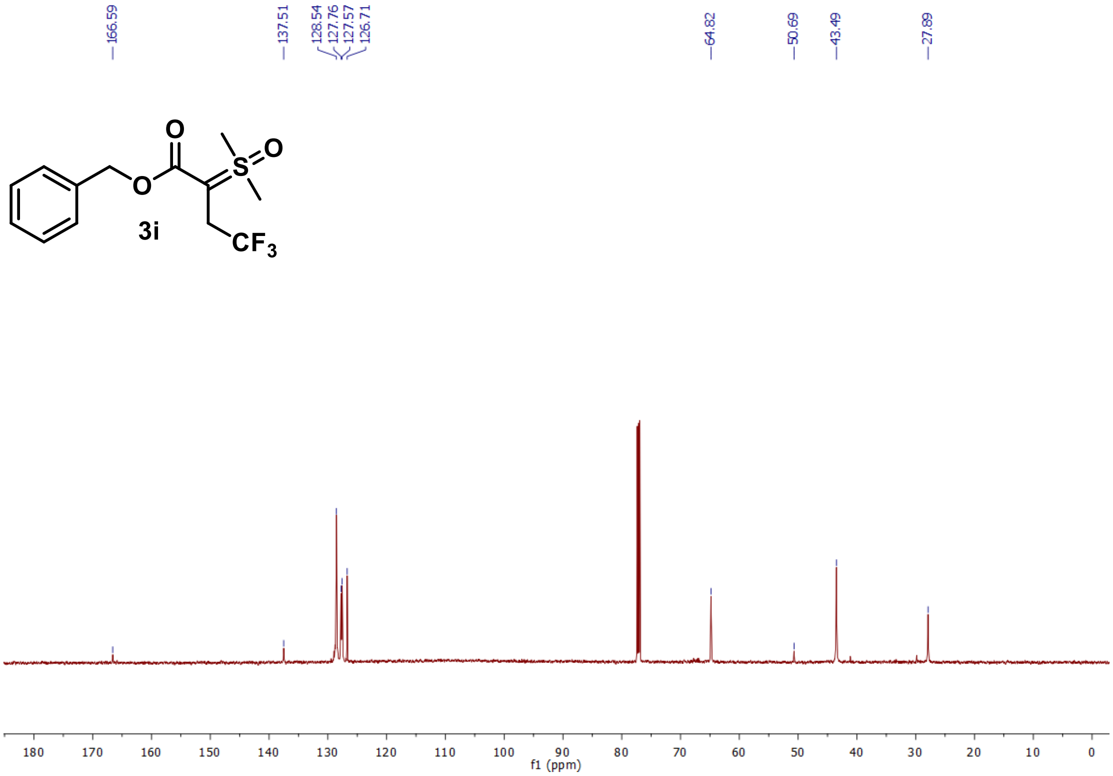

^{19}F NMR (282 MHz, $CDCl_3$) of compound **3i.**

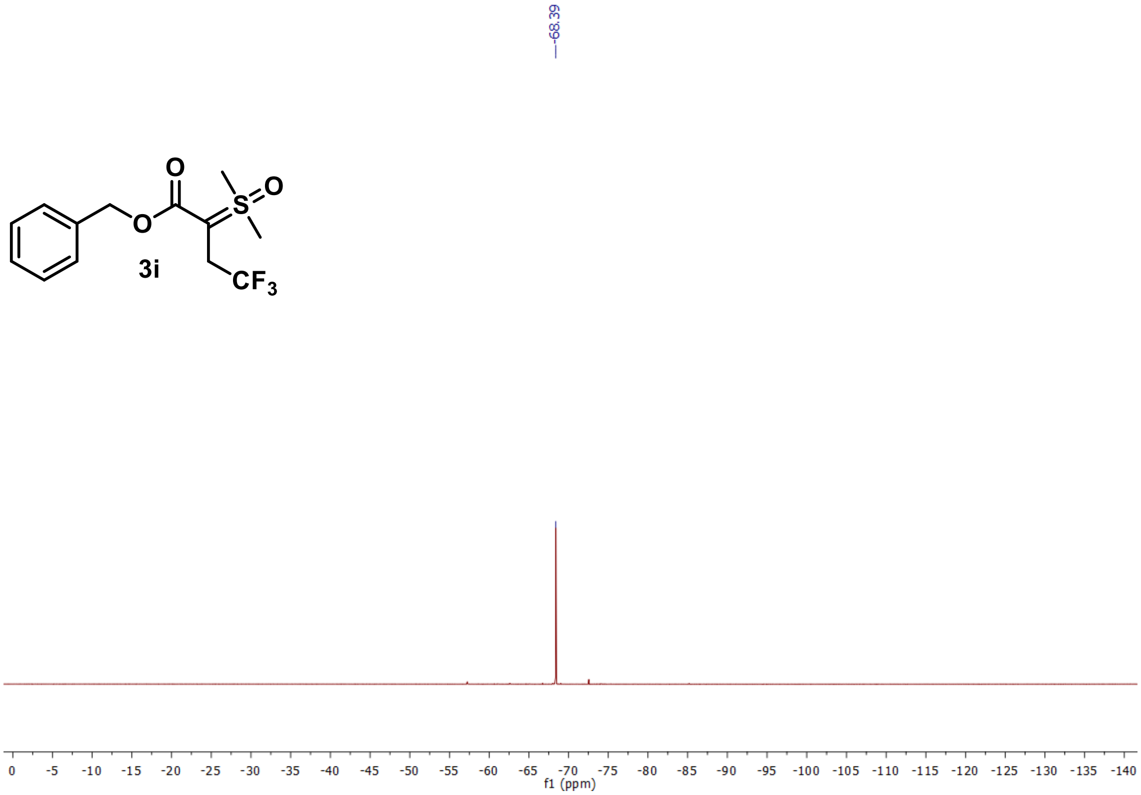

^{1}H **NMR (300 MHz,** $CDCl_3$**)** of compound **3j.**

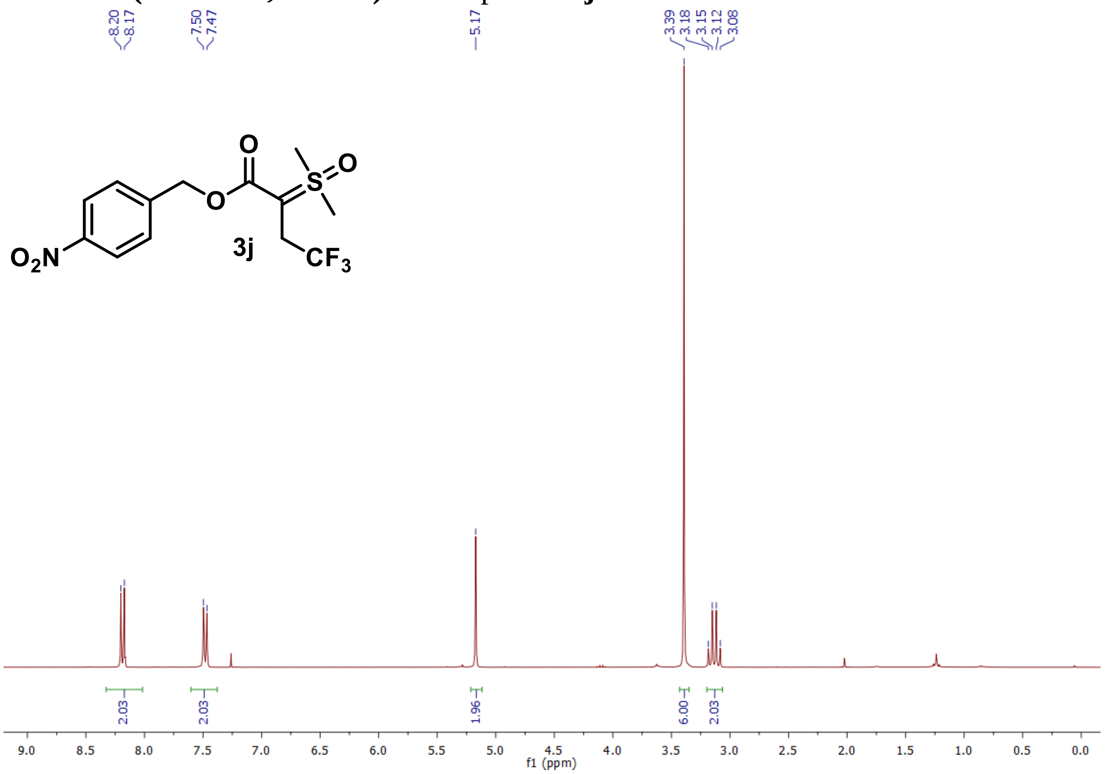

$^{13}C\{^{19}F\}$**(151 MHz,** $CDCl_3$**)** of compound **3j.**

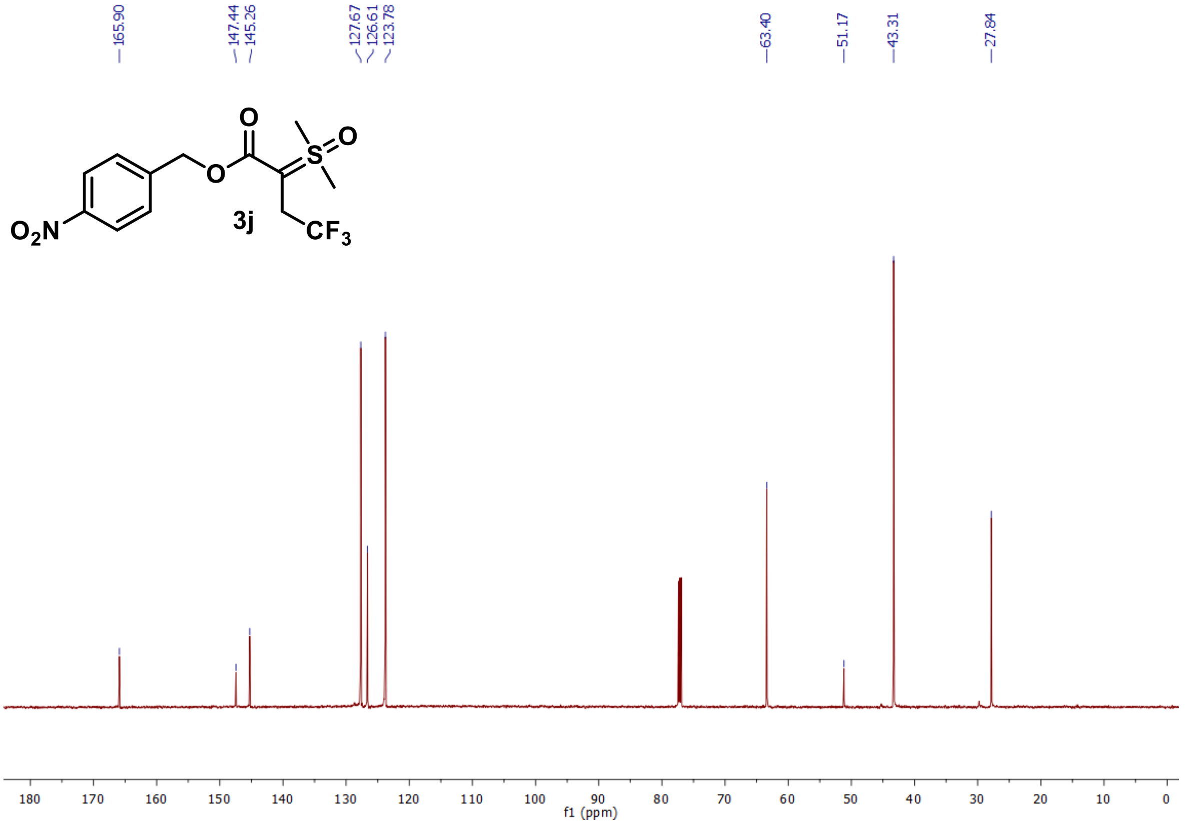

^{19}F NMR (282 MHz, CDCl$_3$) of compound **3j.**

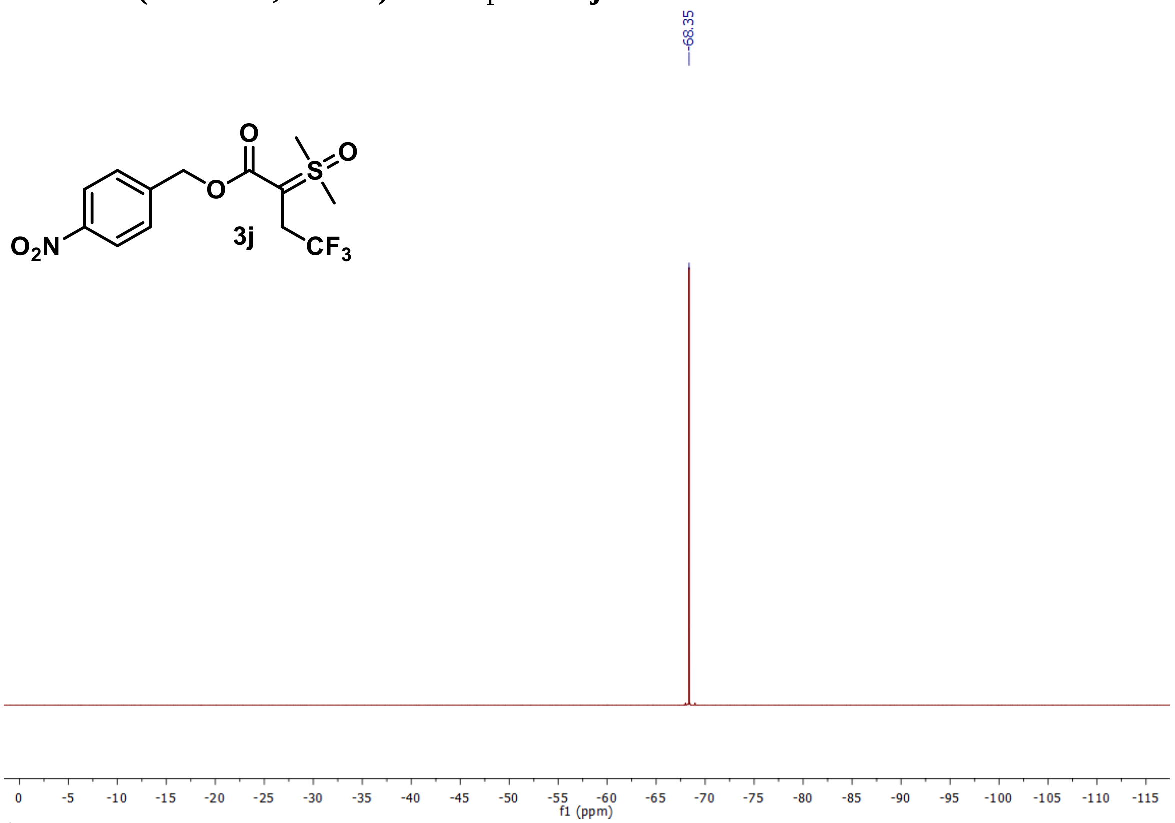

^{1}H NMR (300 MHz, CDCl$_3$) of compound **3k.**

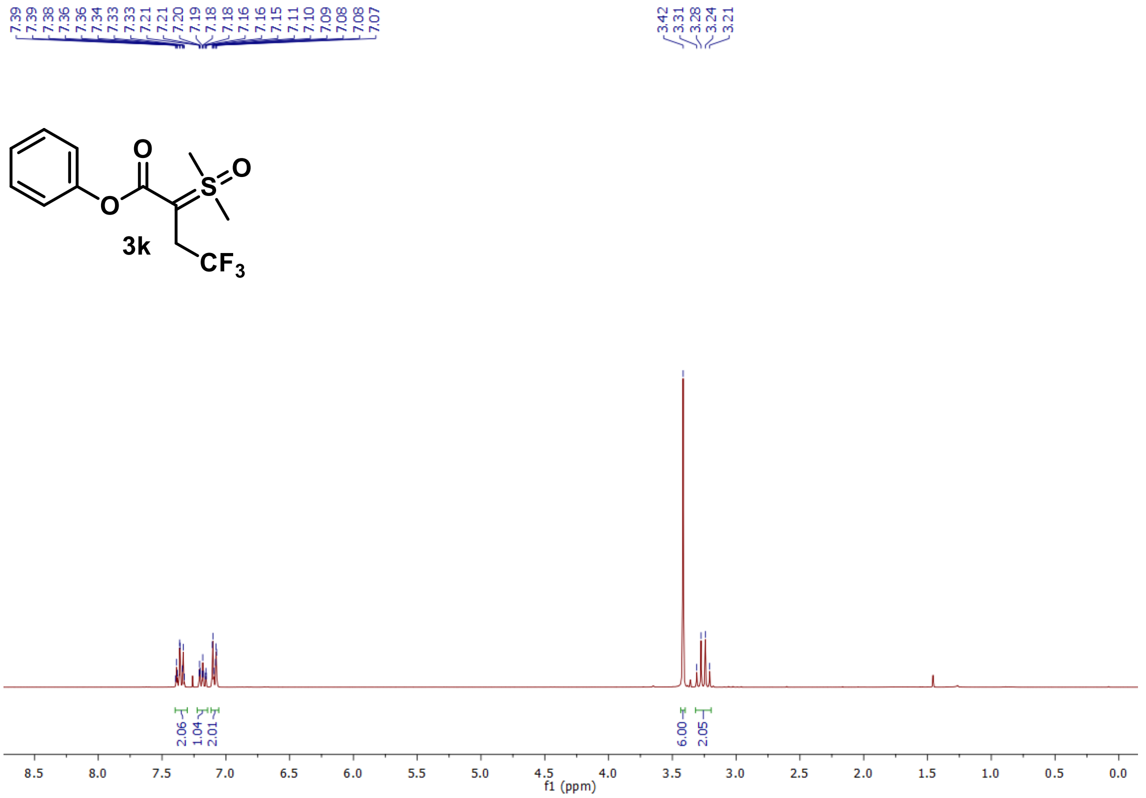

^{13}**C{^{19}F}(151 MHz, CDCl$_3$)** of compound **3k.**

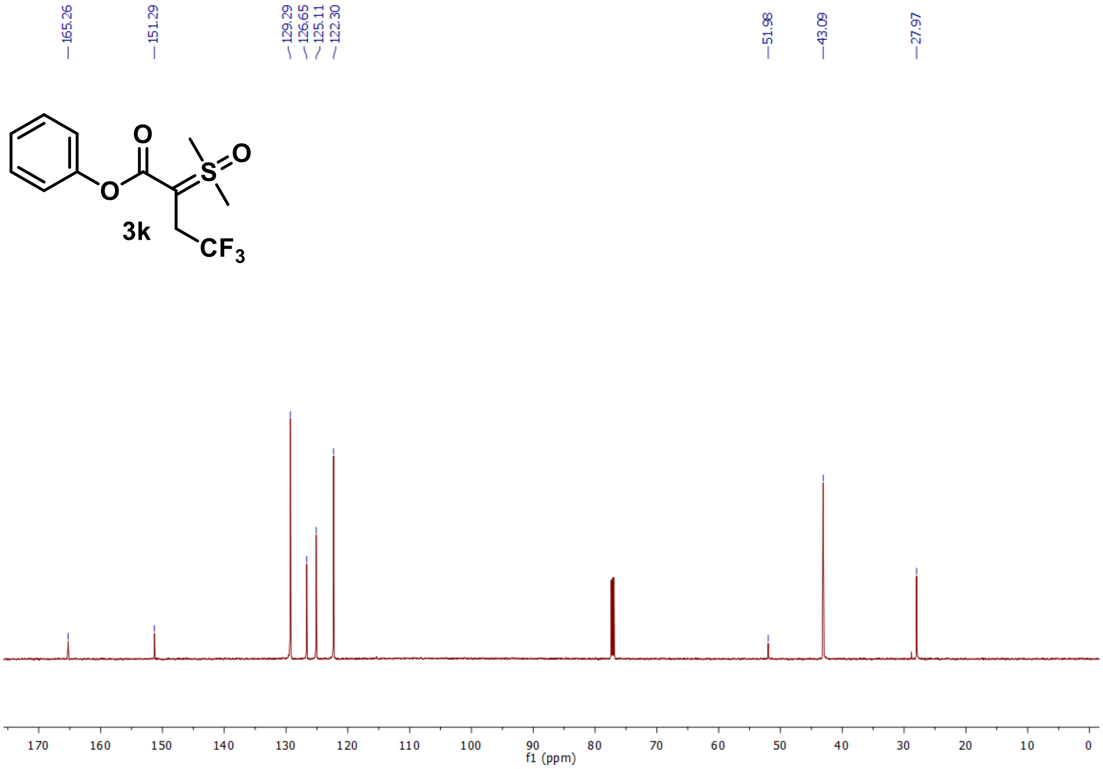

^{19}**F NMR (282 MHz, CDCl$_3$)** of compound **3k.**

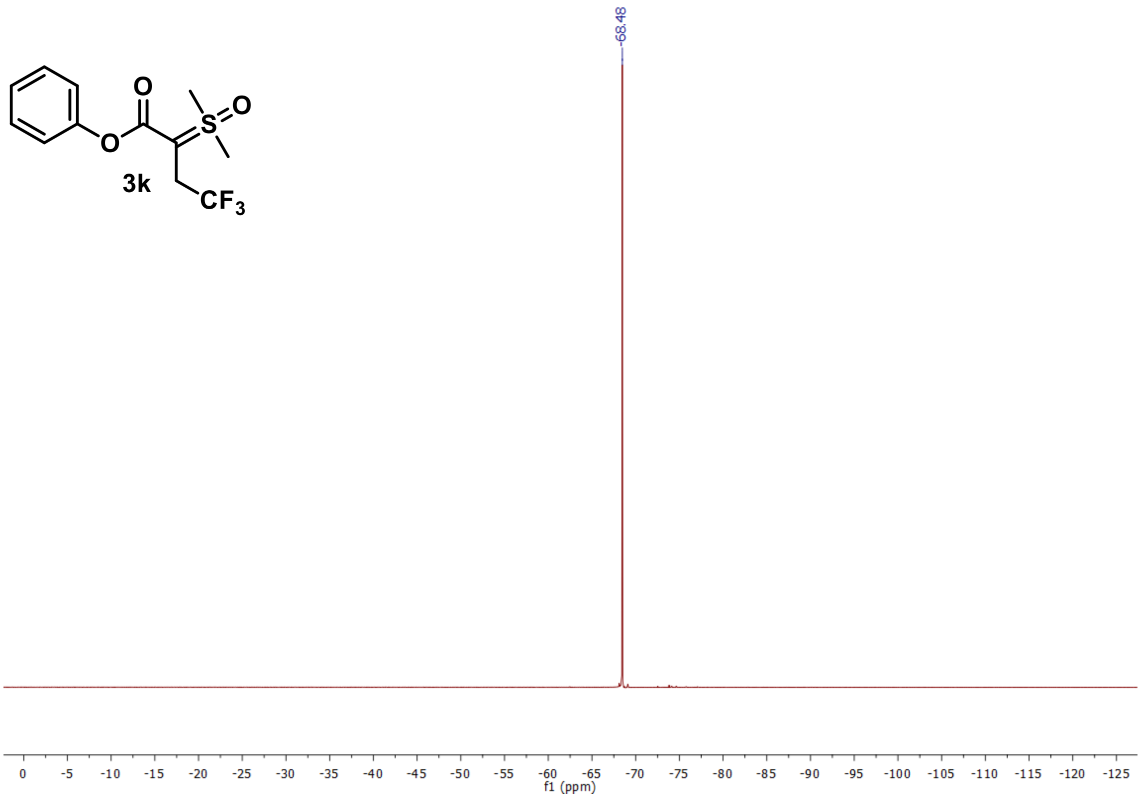

^{1}H NMR (300 MHz, CDCl$_3$) of compound **3l.**

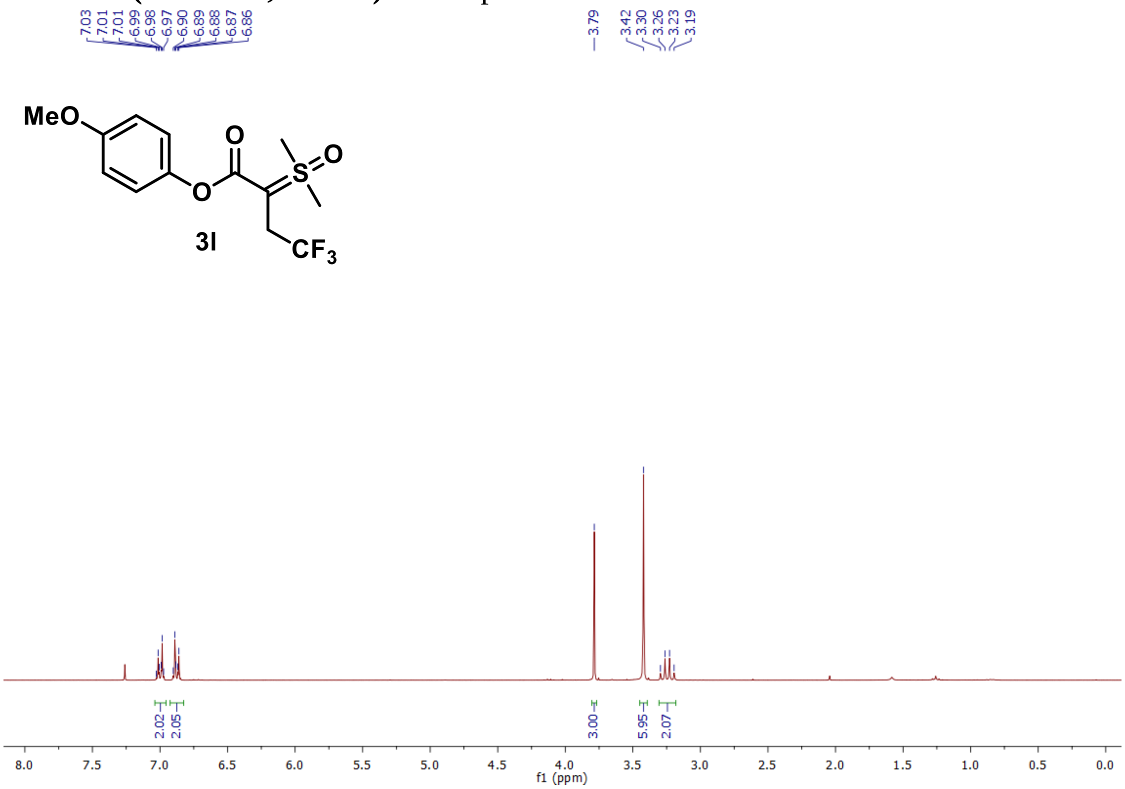

^{13}C{^{19}F}(151 MHz, CDCl$_3$) of compound **3l.**

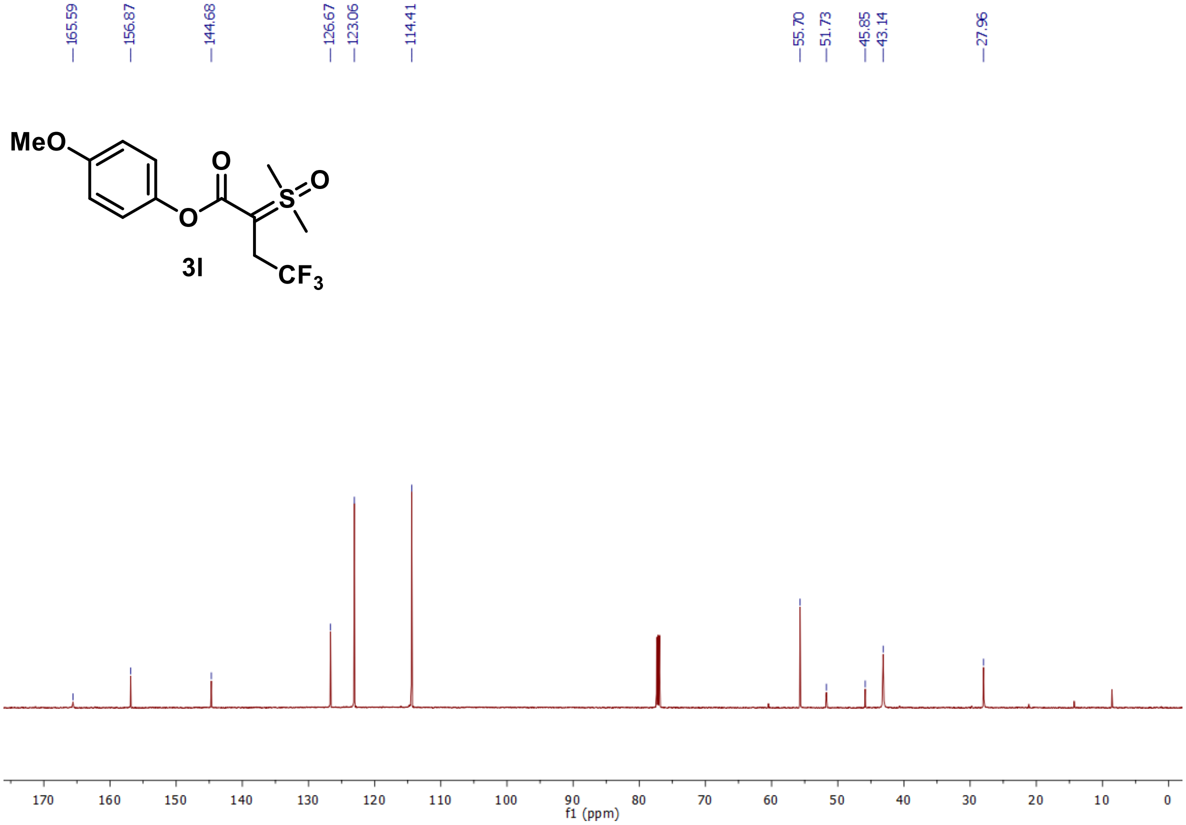

^{19}F NMR (282 MHz, CDCl3) of compound **3l.**

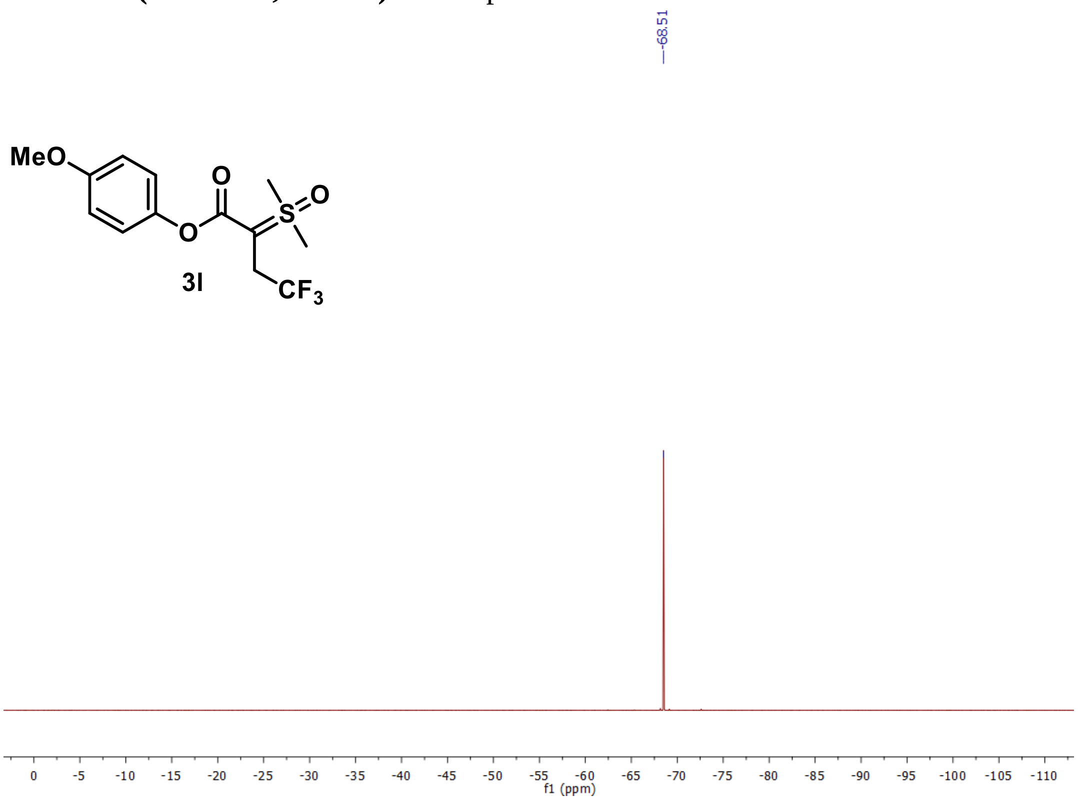

^{1}H NMR (300 MHz, CDCl3) of compound **3m.**

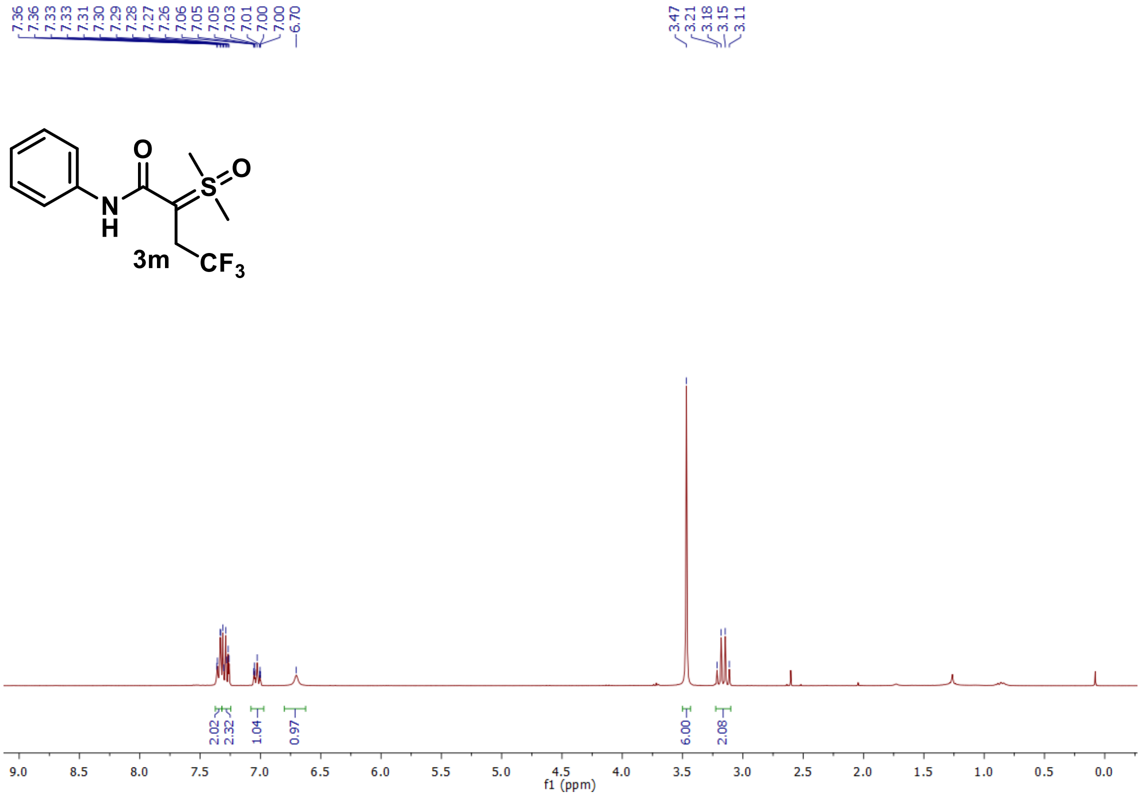

$^{13}C\{^{19}F\}$(151 MHz, $CDCl_3$) of compound **3m.**

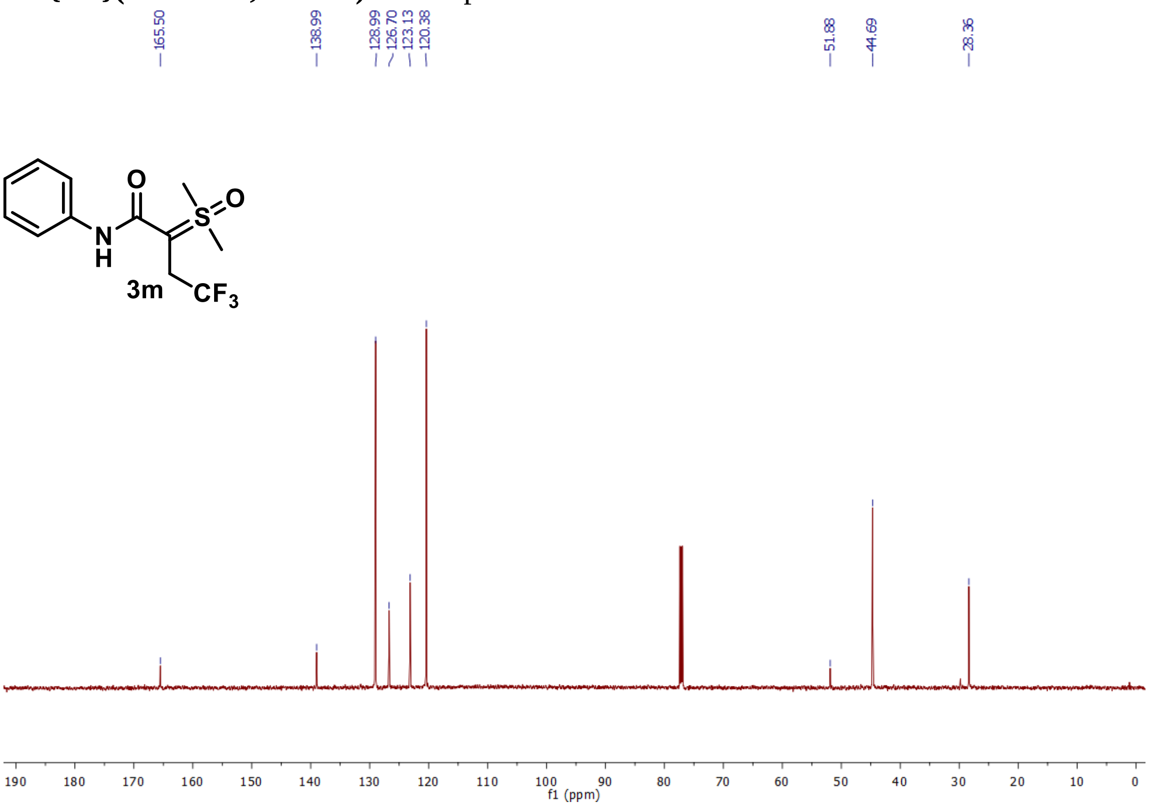

^{19}F NMR (282 MHz, $CDCl_3$) of compound **3m.**

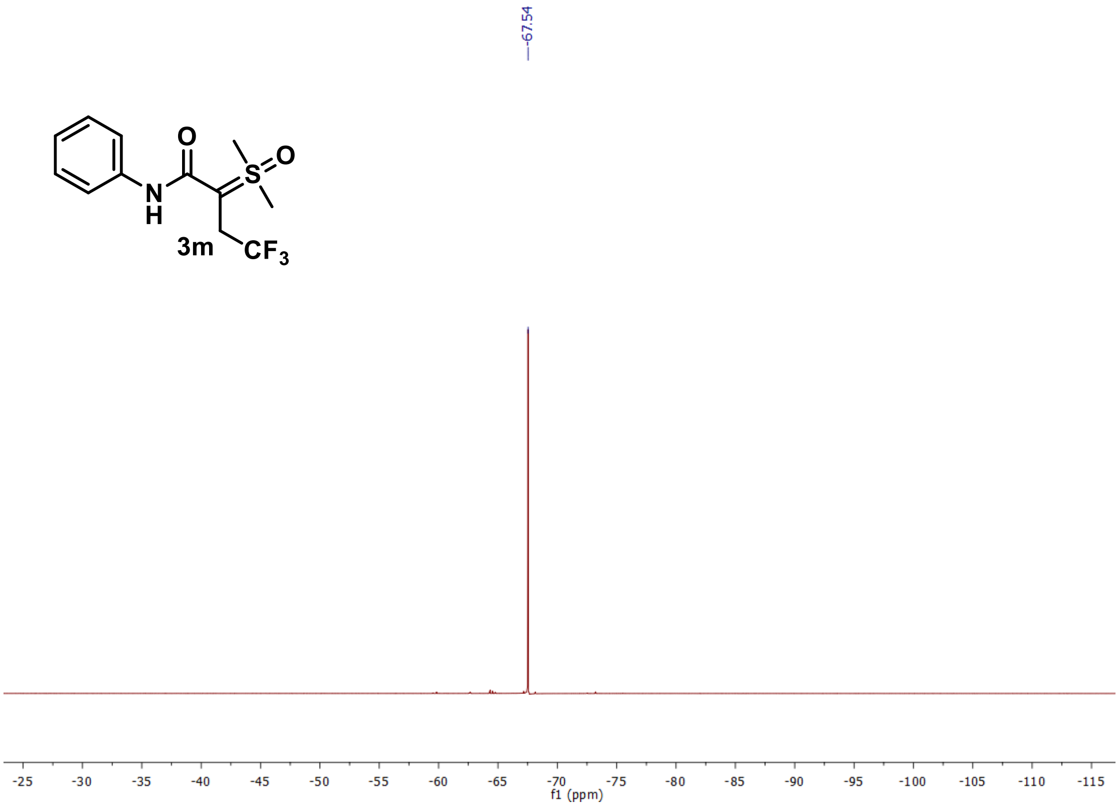

^{1}H NMR (300 MHz, CDCl3) of compound **3n**

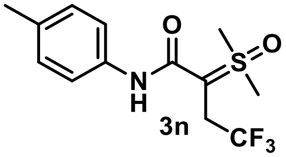

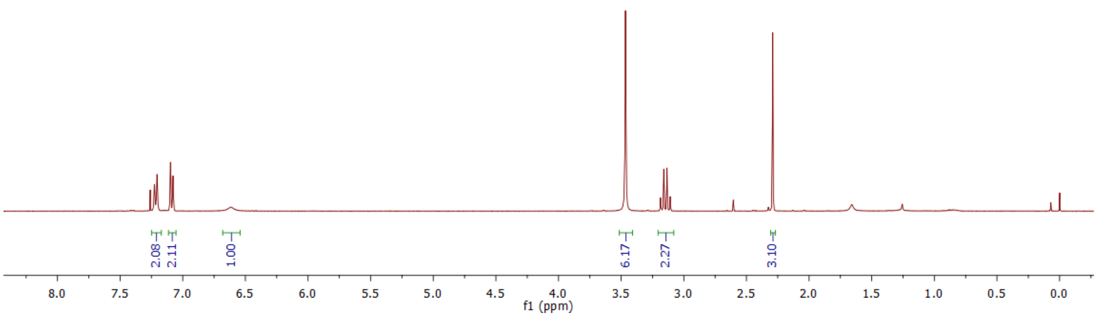

^{13}C(126 MHz, CDCl3) of compound **3n.**

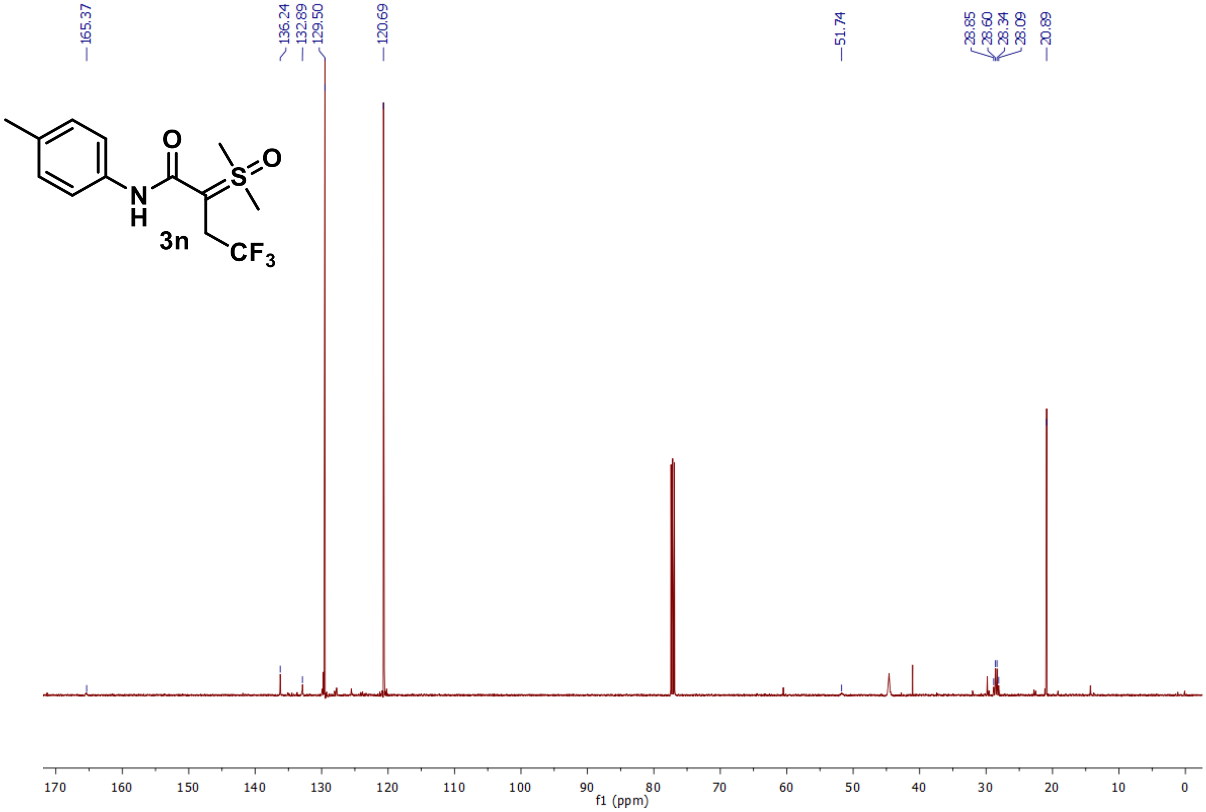

^{19}F NMR (282 MHz, $CDCl_3$) of compound **3m.**

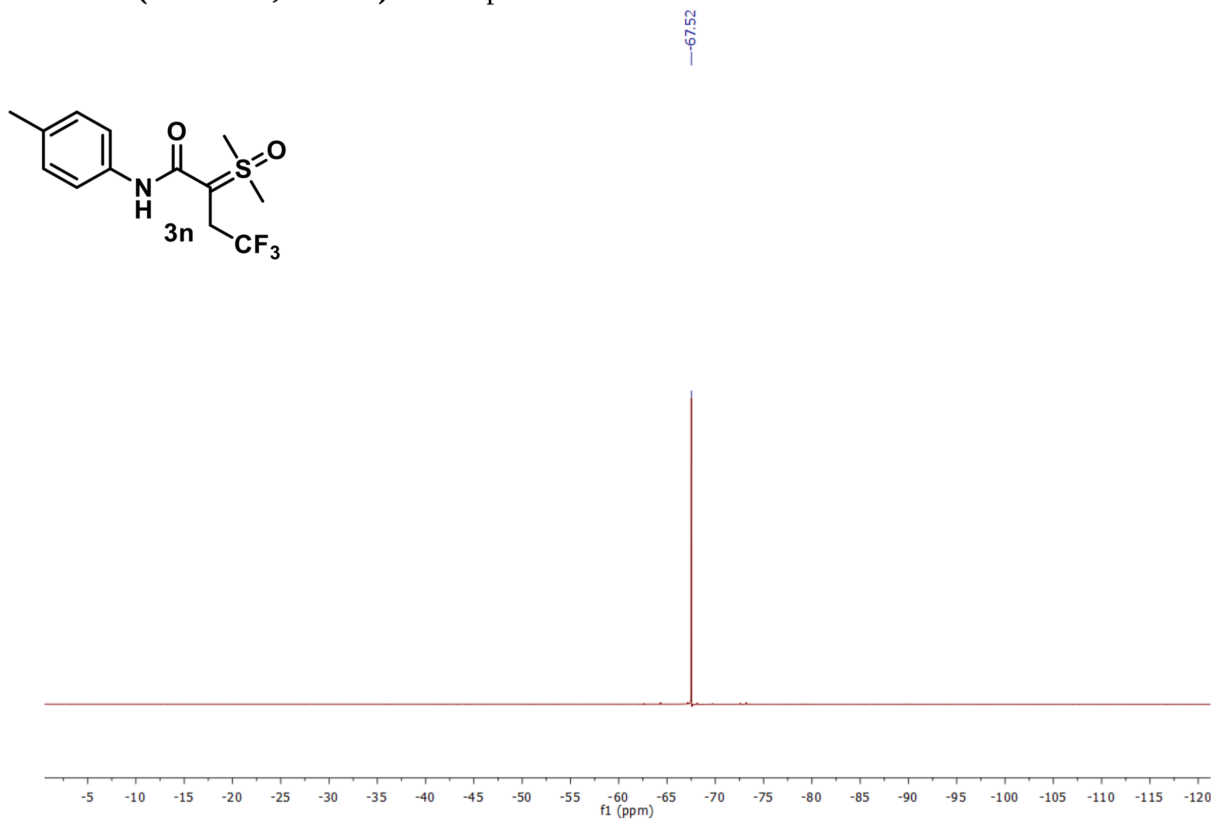

^{1}H NMR (300 MHz, $CDCl_3$) of compound **3o.**

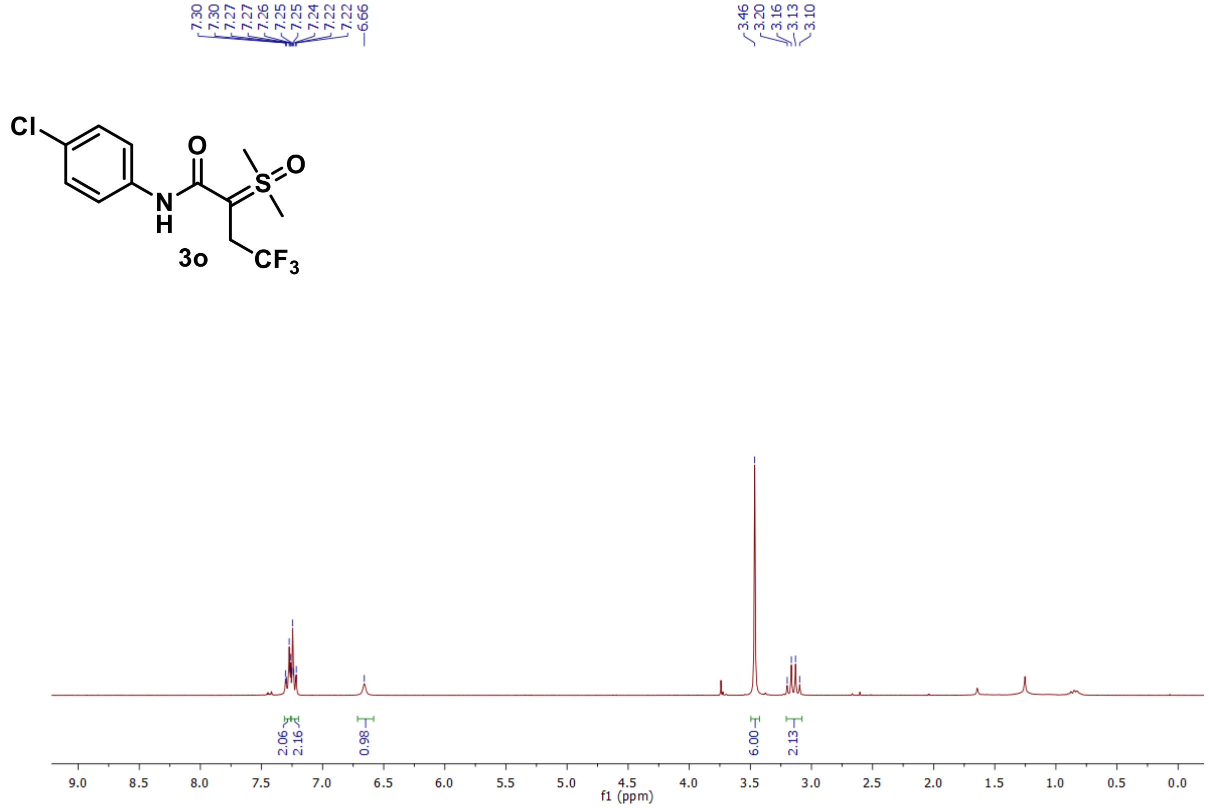

$^{13}C\{^{19}F\}$(151 MHz, $CDCl_3$) of compound **3o.**

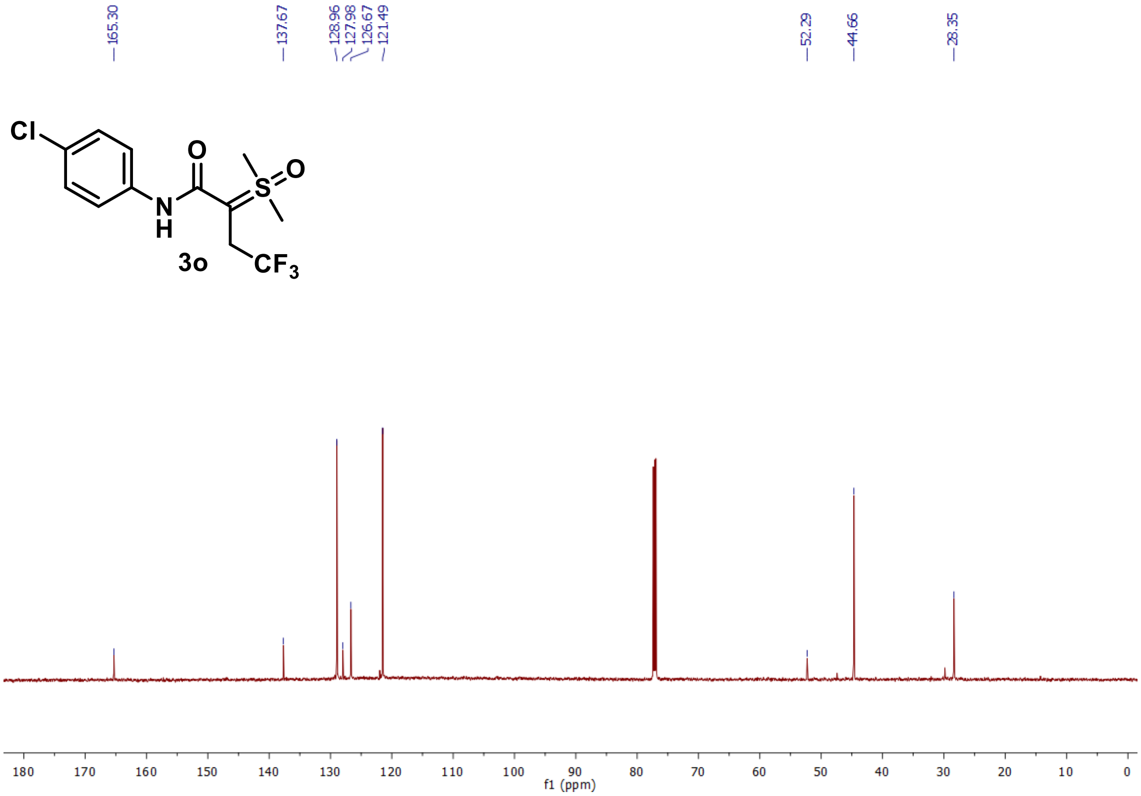

^{19}F NMR (282 MHz, $CDCl_3$) of compound **3o.**

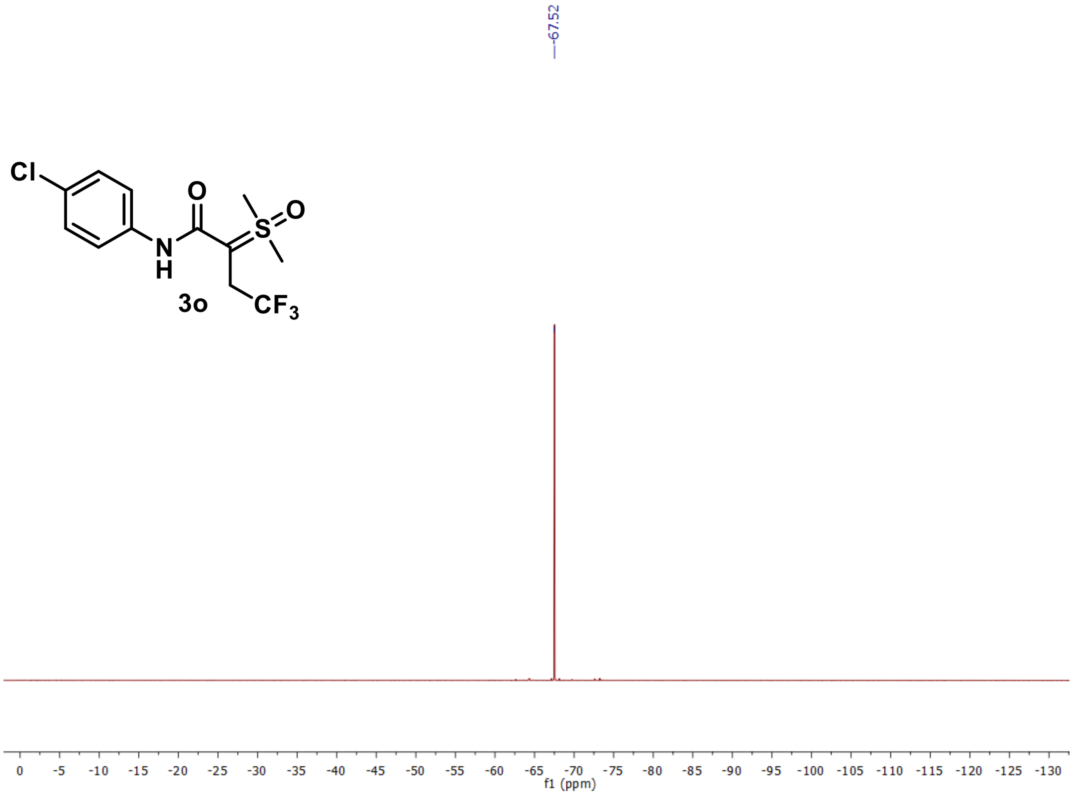

^{1}H NMR (300 MHz, CDCl$_3$) of compound **3p.**

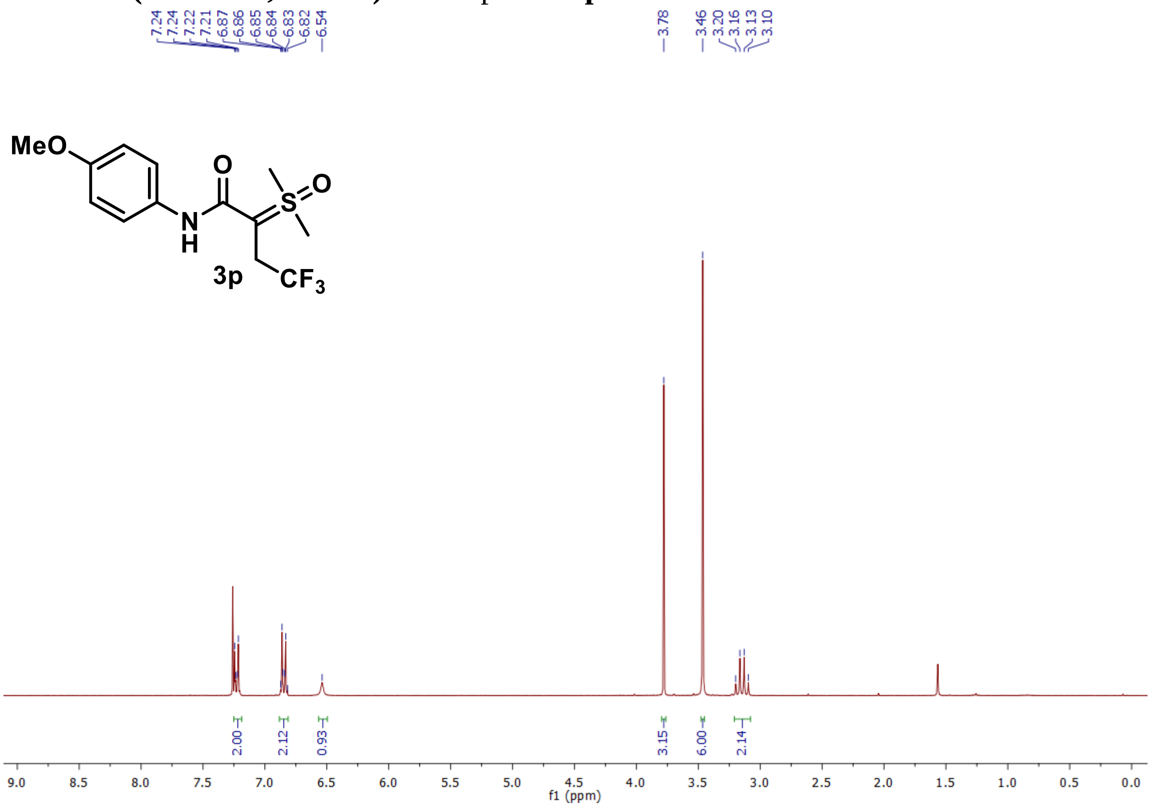

^{13}C{^{19}F}(151 MHz, CDCl$_3$) of compound **3p.**

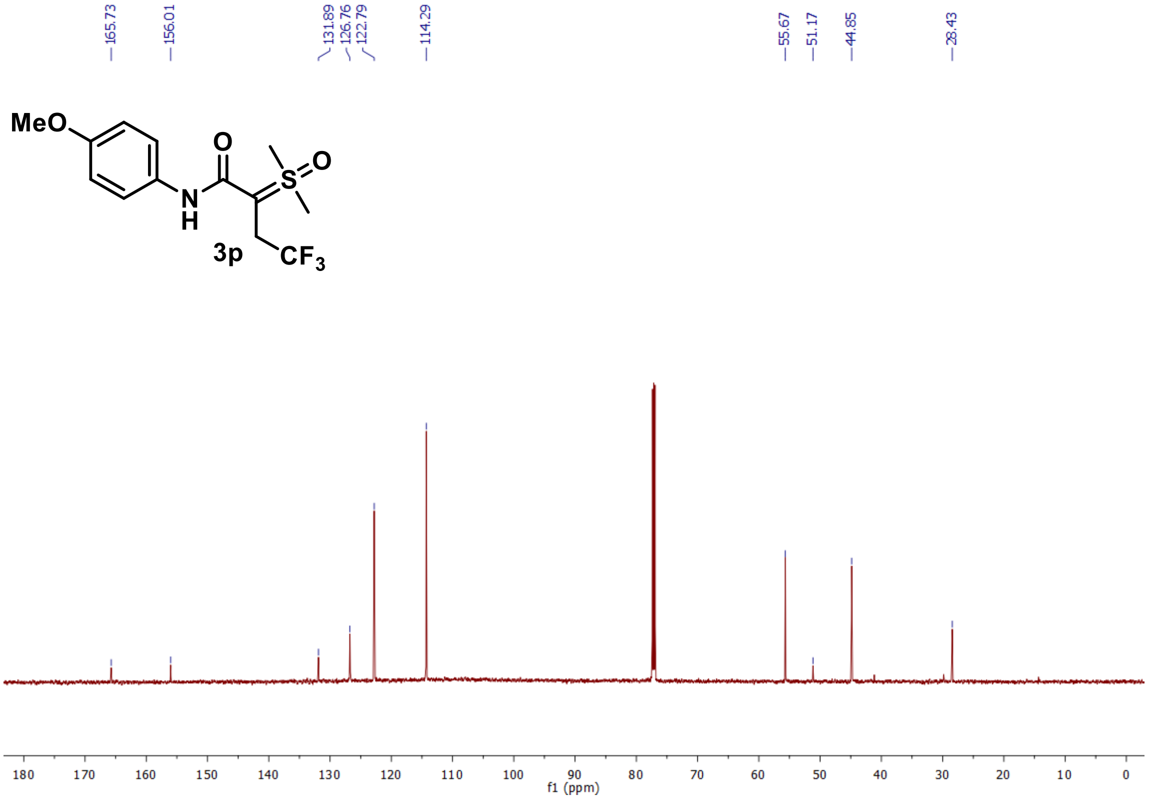

^{19}F NMR (282 MHz, $CDCl_3$) of compound **3p.**

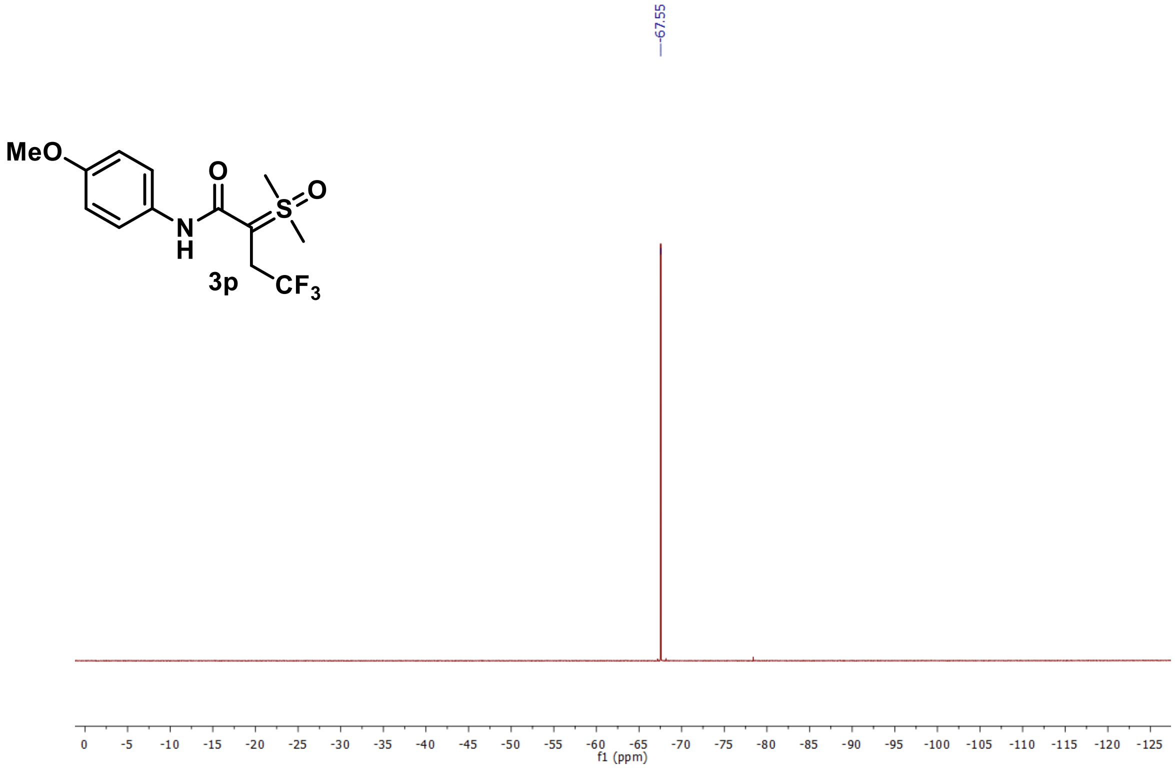

^{1}H NMR (300 MHz, $CDCl_3$) of compound **3q.**

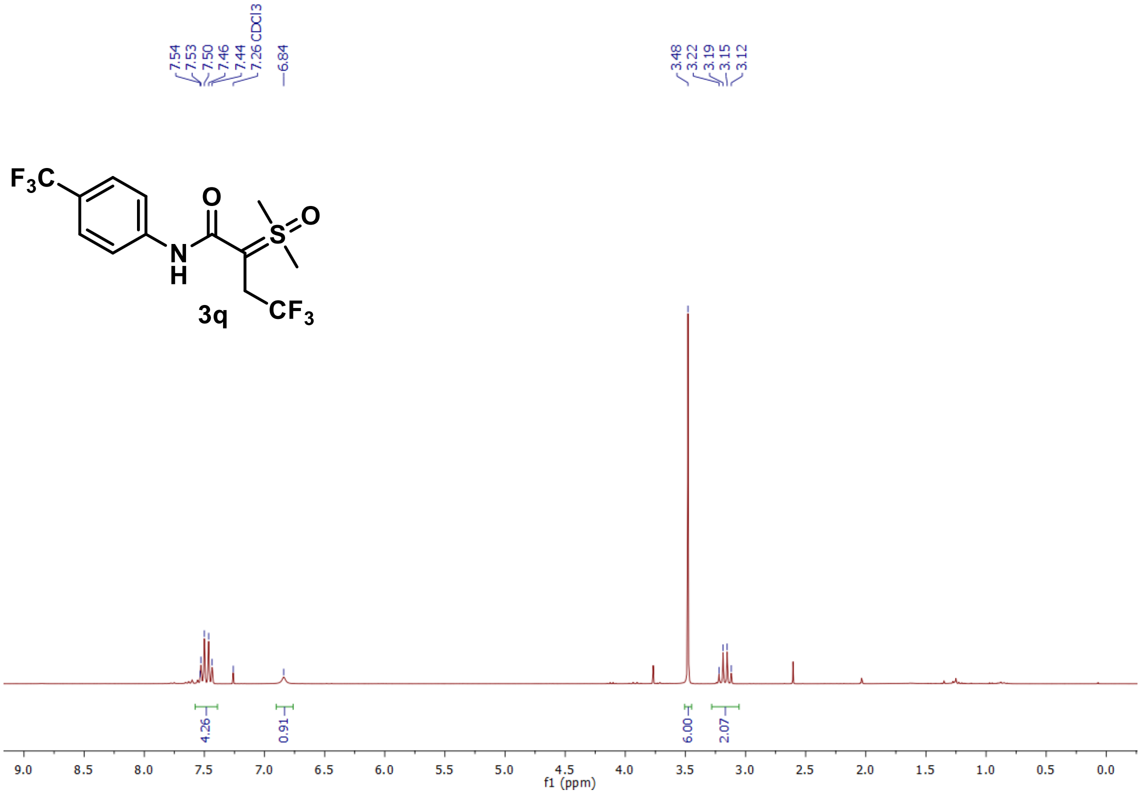

^{13}C{^{19}F}(151 MHz, CDCl$_3$) of compound **3q.**

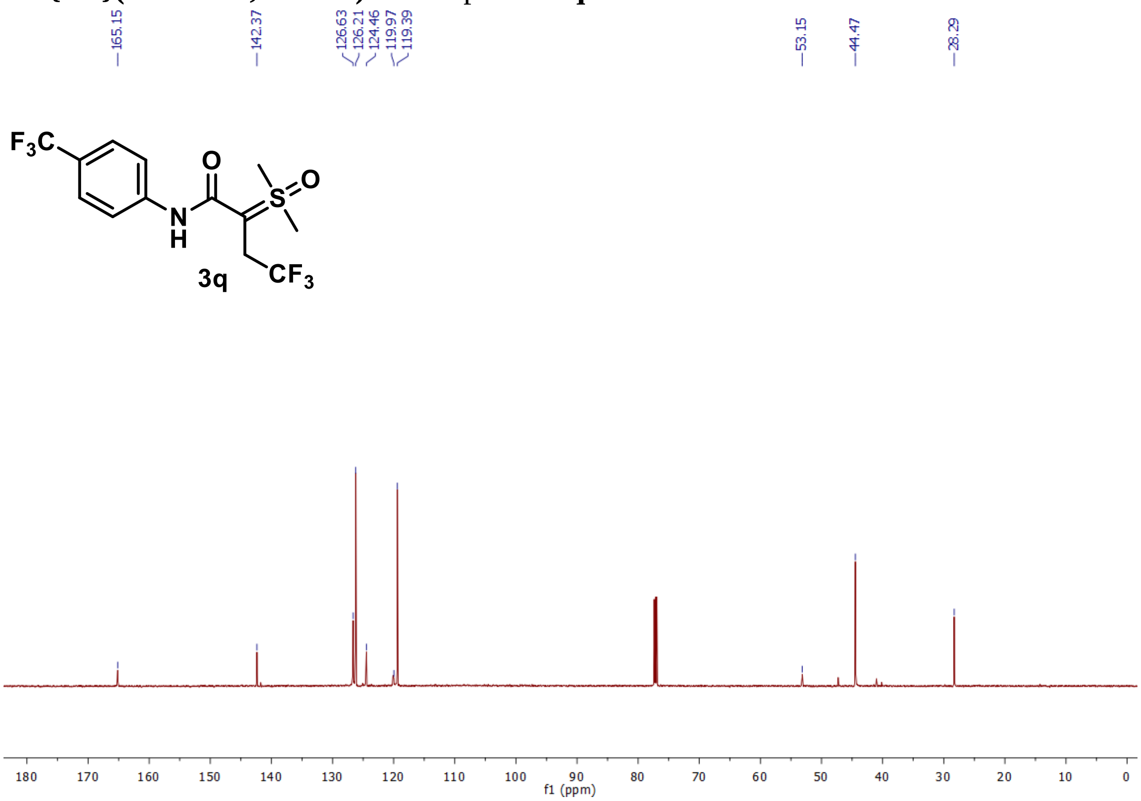

^{19}F NMR (282 MHz, CDCl$_3$) of compound **3q.**

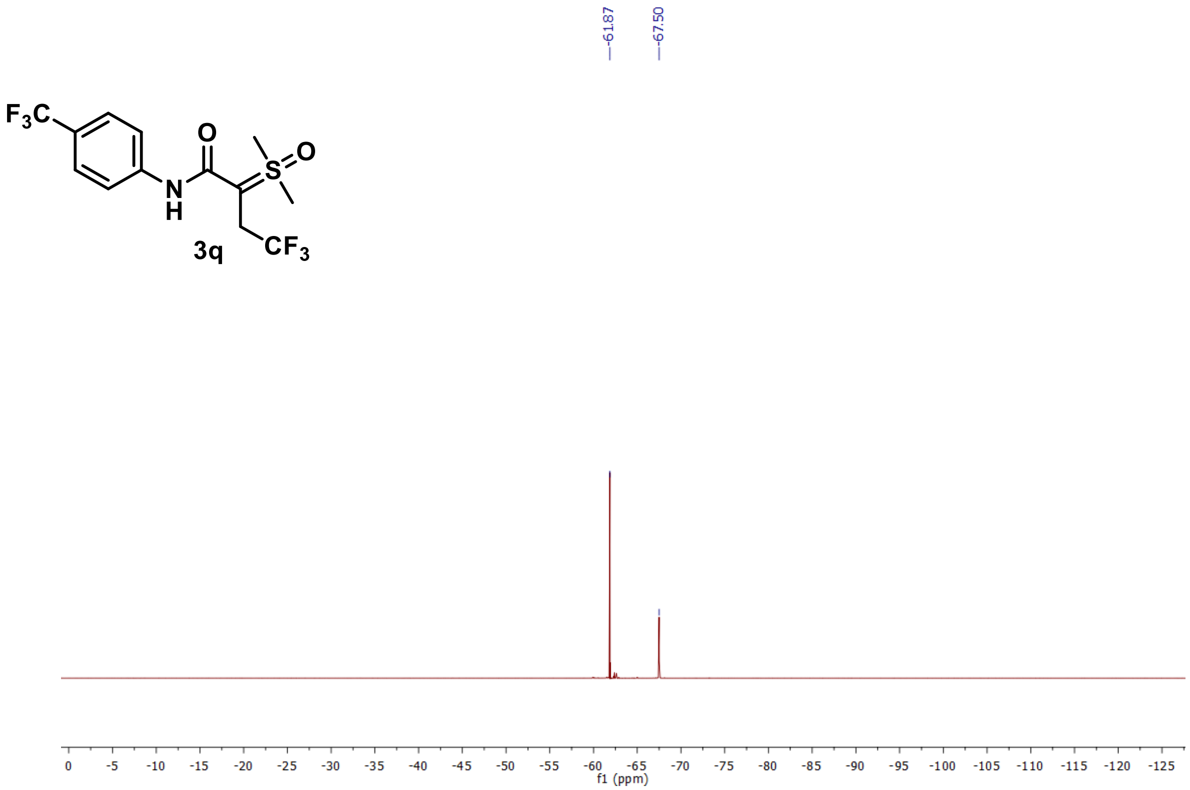

^{1}H NMR (300 MHz, $CDCl_3$) of compound **3r.**

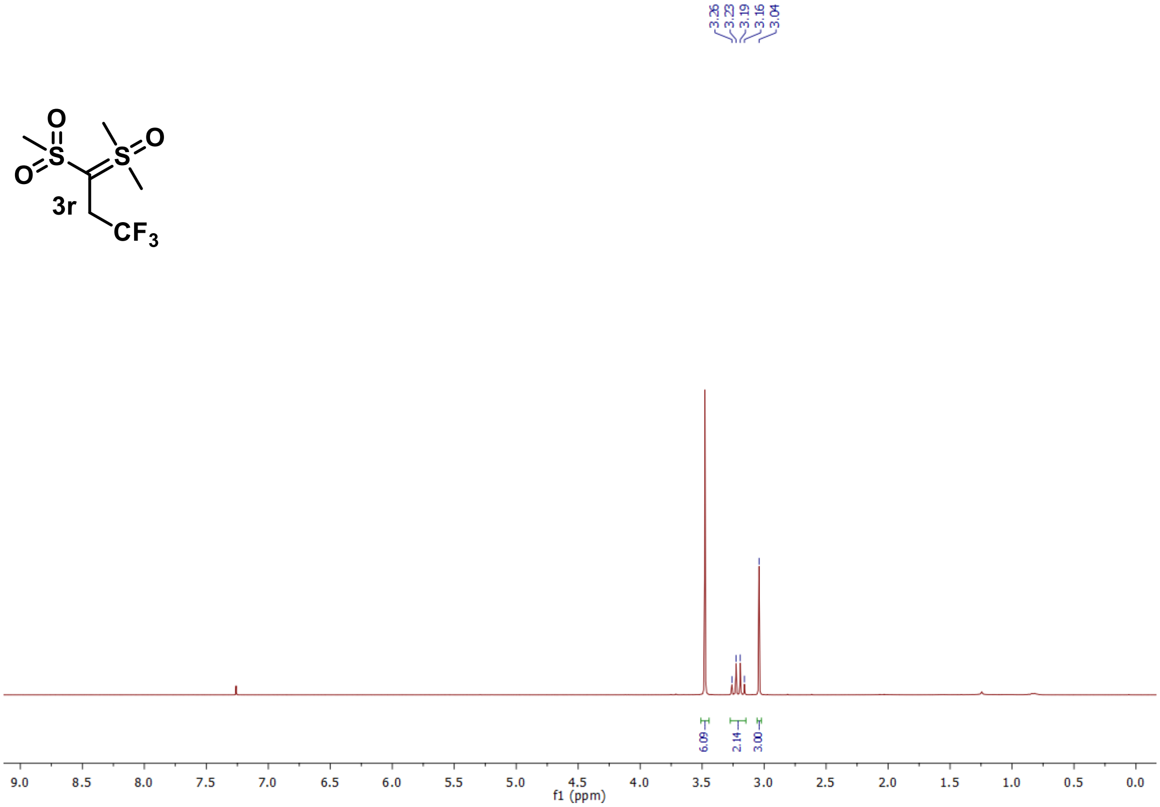

^{13}C{^{19}F}(151 MHz, $CDCl_3$) of compound **3r.**

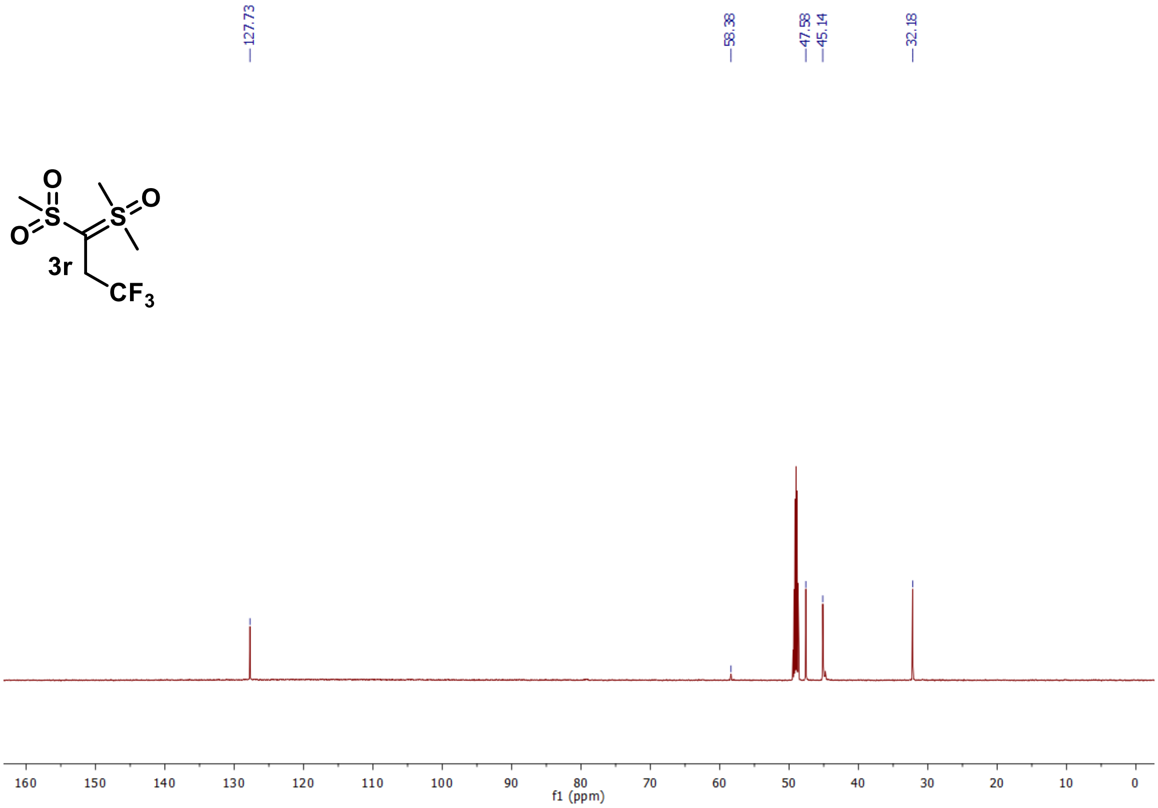

^{19}F NMR (282 MHz, CDCl3) of compound **3r.**

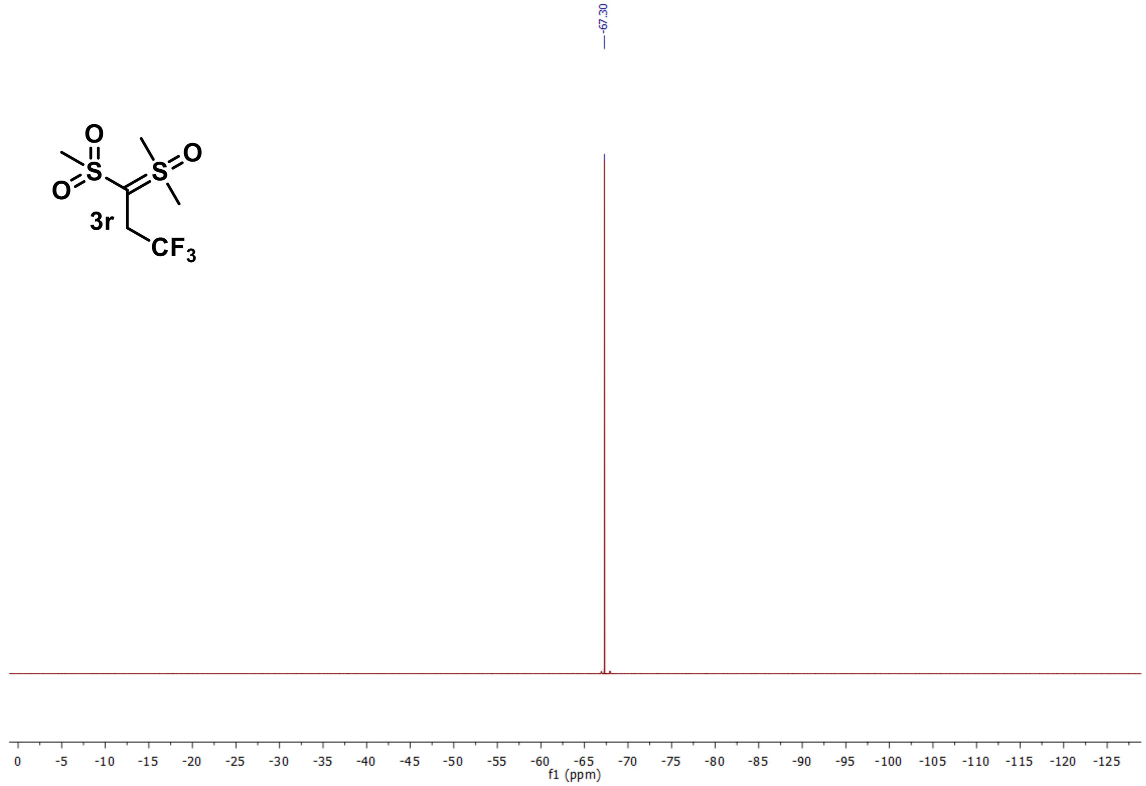

^{1}H NMR (300 MHz, CDCl3) of compound **3s.**

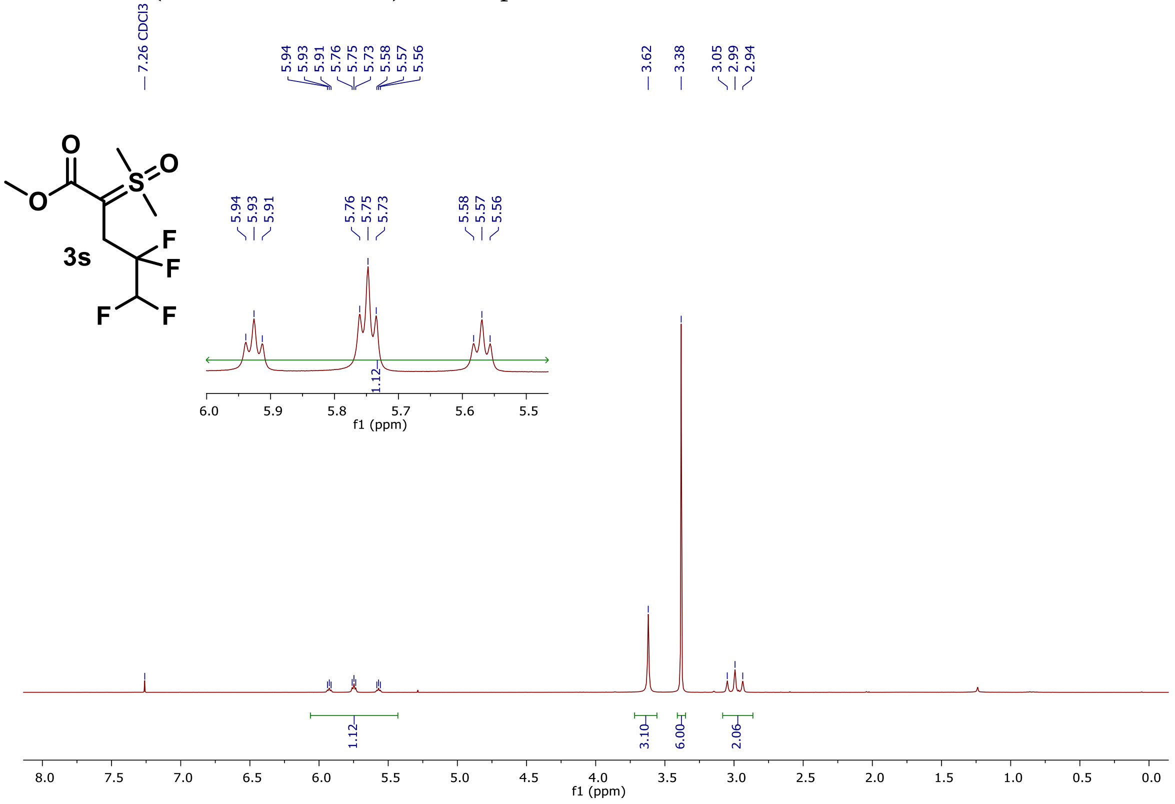

^{13}**C{^{19}F}(151 MHz, CDCl$_3$)** of compound **3s.**

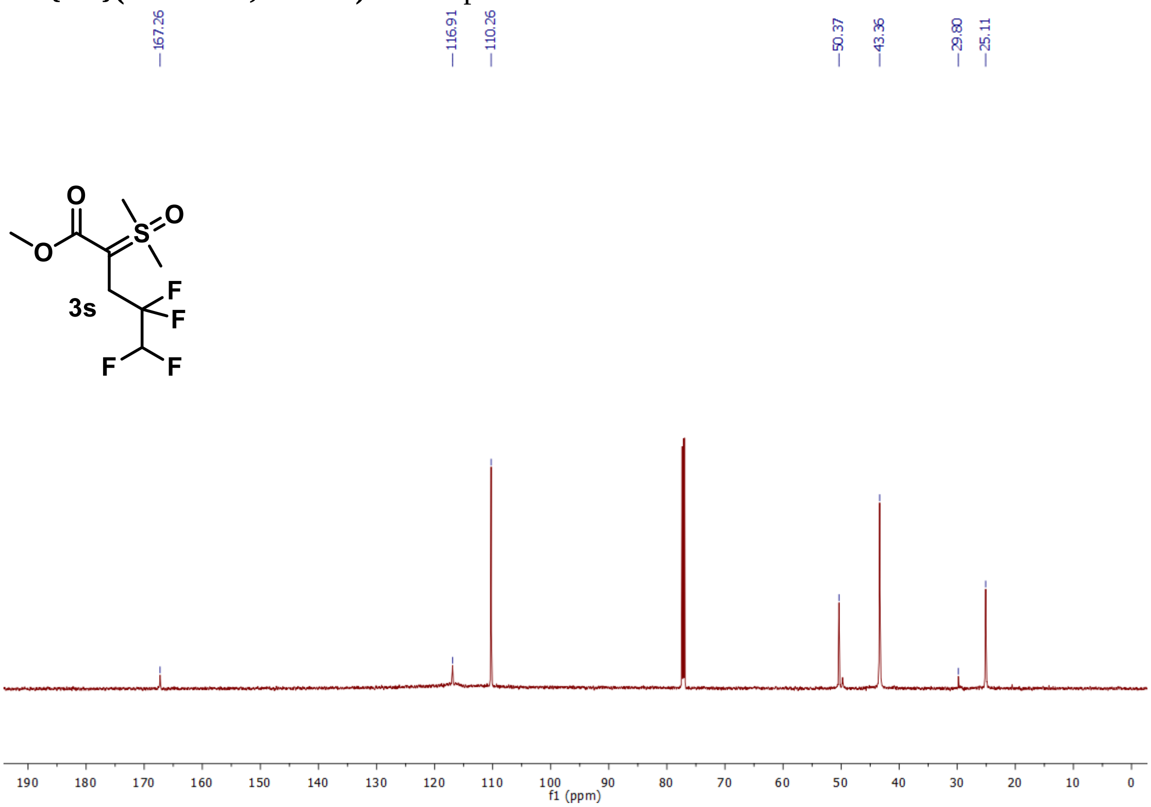

^{19}**F NMR (282 MHz, CDCl$_3$)** of compound **3s.**

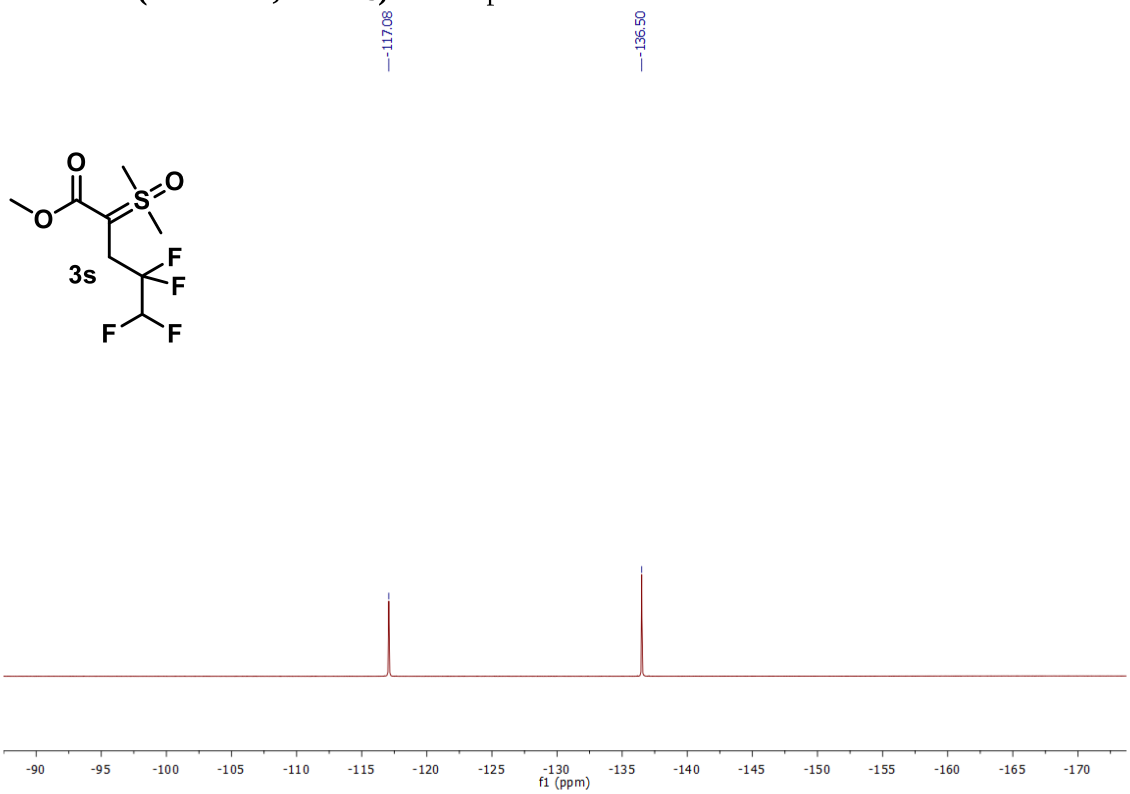

^{1}H NMR (400 MHz, CDCl$_3$) of compound **4.**

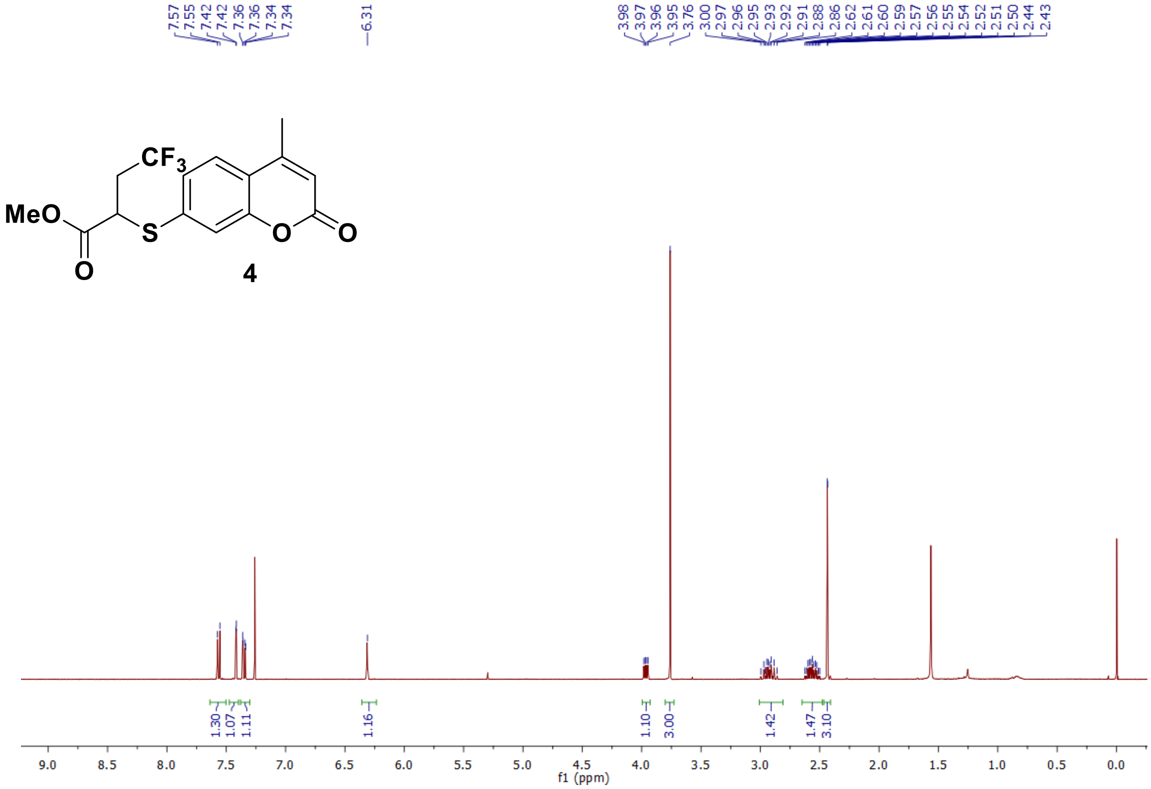

^{13}C NMR (101 MHz, CDCl$_3$) of compound **4**.

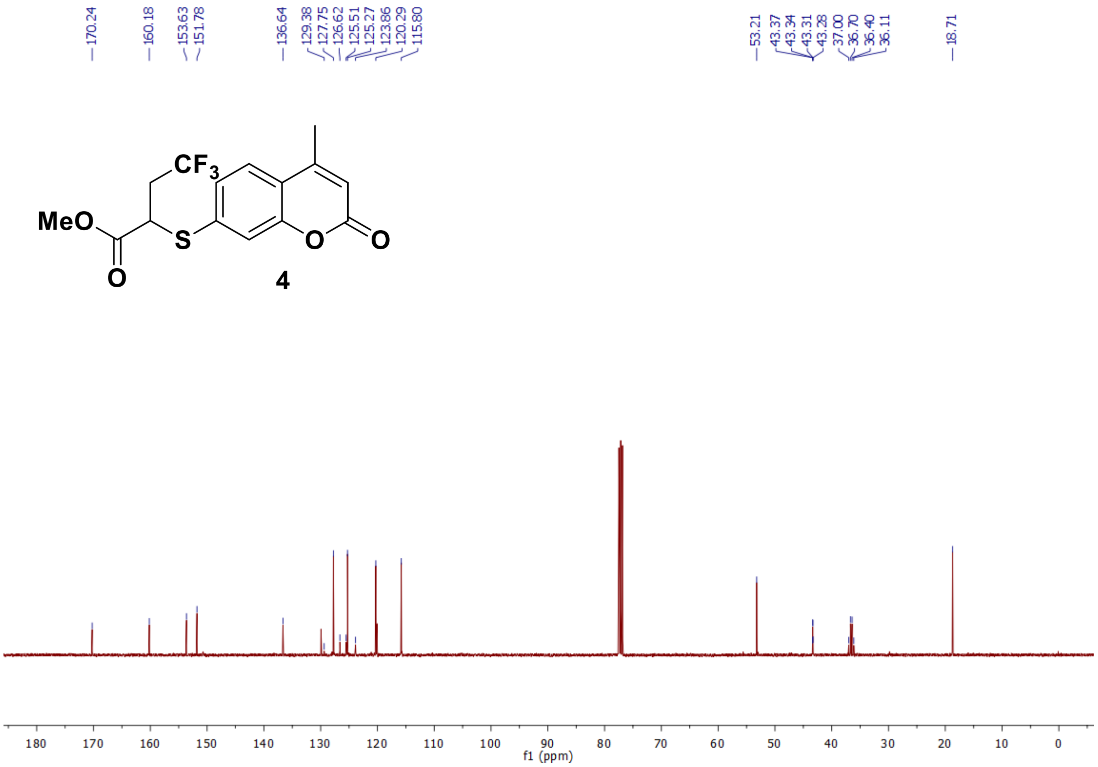

^{19}F NMR (282 MHz, $CDCl_3$) of compound **4.**

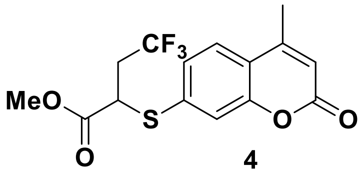

4

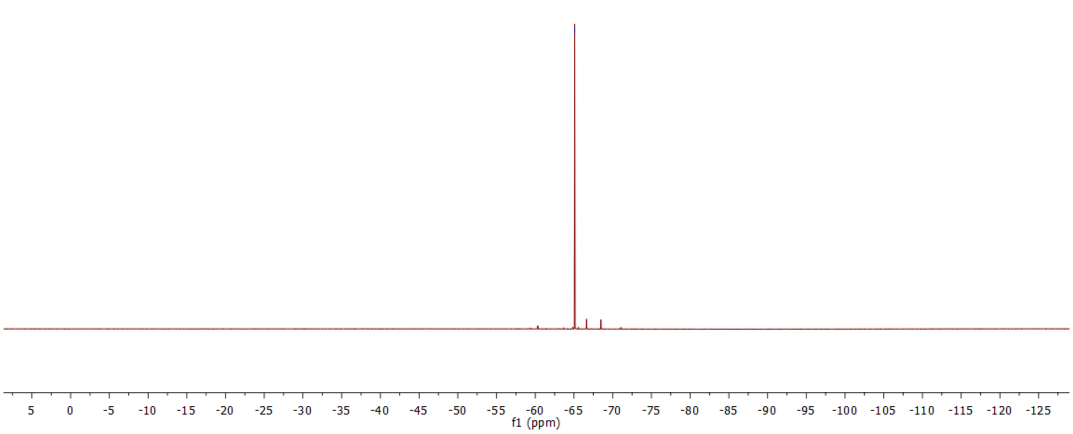

4. Computational general details

For all starting materials, conformational analysis was conducted using the Crest program.[1] Pre-optimization was conducted on the lowest energy conformers using Gaussian16[2] at the PBE0 level of theory[3, 4] with def2-TZVP as the basis set for all atoms except iodine, for which def2-TZVPD was employed.[5-7] Final optimizations and frequency calculations of all structures were conducted using Orca 5.0.1 at the PBE0/def2-TZVPD, the auto auxiliary method, and the defgrid3 integration grid.[8-10] Transition states were confirmed by imaginary frequencies and using the intrinsic reaction coordinate (IRC) method.[11] A continuum solvation model in acetonitrile was used for all calculations either SMD for PES scan or CPCM for all Orca calculations.[12] Visualization of results was conducted in GaussView6[13] for Gaussian16 calculations or Avogadro 1.2.0[14] for all Orca calculations. Final geometries for publication purposes were prepared in the Visual Molecular Dynamics software (VMD).[15] The electrostatic potential map for 2,2,2-

trifuoroethyl(mesityl)iodonium ion **2a’** was prepared from the pre-optimized structure in Gaussview6 with an isodensity surface of 0.001 au.[16]

4.1 PES scan results

Starting from the pre-optimized structures of **XB-2** and **A**, a modredundant potential energy surface (PES) scan was conducted using Gaussian16. Starting at 3.0 Å, the bond length between C2 and C15 was decreased over increments of -0.1 Å (path 1) or -0.08 Å (path 2) to reveal potential saddle points. The level of theory used was semi-empirical/PM6 with the SMD solvation model in acetonitrile. Results were visualized in GaussView6 to reveal two potential saddle points at activation energies of 49.5 kcal/mol and 30.8 kcal/mol for the reductive elimination (Path 1) and nucleophilic substitution (Path 2) mechanisms, respectively.

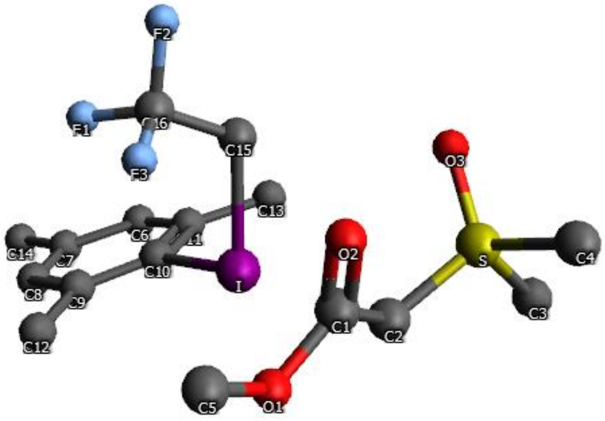

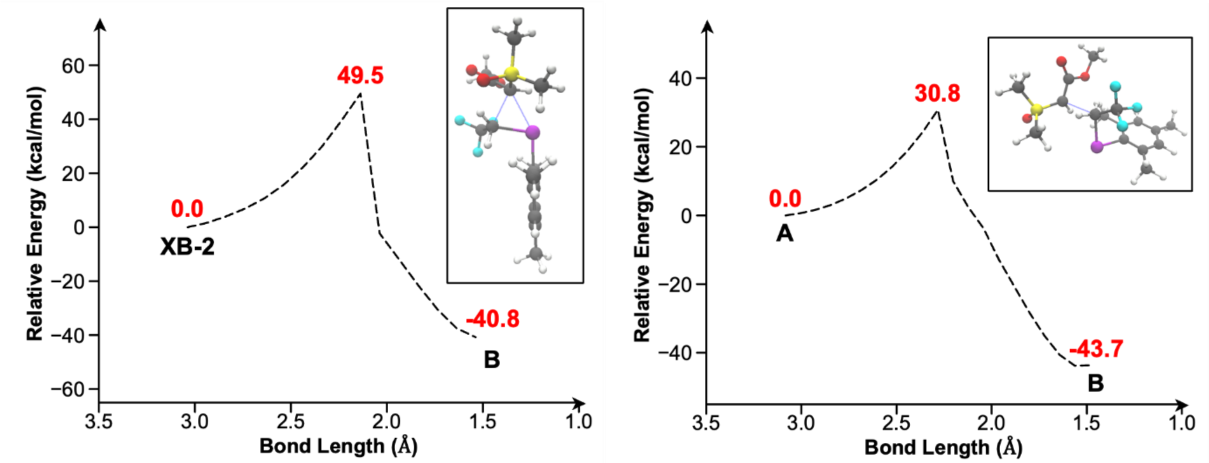

Figure S2. Modredundant scan for both reaction pathways at the semi-empirical/PM6 level of theory from 3.0 Å to 1.5 Å. **Path 1** (left) and **Path 2** (right) and converged geometry identified at the saddle point (black outline).

Table S2. Scan data for **Path 1**

**Scan Coordinate**	**Total Energy (Hartree)**
3.037848765	-0.302713134
2.937848765	-0.300165061
2.837848765	-0.296323878
2.737848765	-0.29167857
2.637848765	-0.285663961
2.537848765	-0.277651153
2.437848765	-0.267398263
2.337848765	-0.255002387
2.237848765	-0.239941282
2.137848765	-0.223794677
2.037848765	-0.306409381
1.937848765	-0.321603442
1.837848765	-0.336938397
1.737848765	-0.351121336
1.637848765	-0.362334099
1.537848765	-0.367711837

Table S3. Scan data for **Path 2**

**Scan Coordinate**	**Total Energy (Hartree)**
3.082745478	-0.298867024
3.002745478	-0.297786069
2.922745478	-0.296180735
2.842745478	-0.293934693
2.762745478	-0.290874335
2.682745478	-0.286995445
2.602745478	-0.282267327
2.522745478	-0.276250213
2.442745478	-0.268927357
2.362745478	-0.26020523
2.282745478	-0.249862372
2.202745478	-0.282908047
2.122745478	-0.295041853
2.042745478	-0.304841368
1.962745478	-0.319249744
1.882745478	-0.33154643
1.802745478	-0.343774251
1.722745478	-0.354668068
1.642745478	-0.363564569
1.562745478	-0.368627882
1.482745478	-0.368430154

4.2 NEB results

Starting from **XB-2**, **A**, and **B**, a climbing image (CI) search was conducted using the nudged elastic band (NEB) method in Orca 5.0.1. PBE/def2-SVP D4[17, 18] level of theory and the CPCM solvation model were implemented with acetonitrile with tight convergence criteria. All structures were pre-optimized at the same level of theory before the NEB-CI run was initiated. For both NEB-CI pathways, the calculation was performed at multiple orientations of **1a** with respect to 2,2,2-trifuoroethyl(mesityl)iodonium ion **2a'**, shown below are the successful calculations that give rise to opposite enantiomers of **B**.

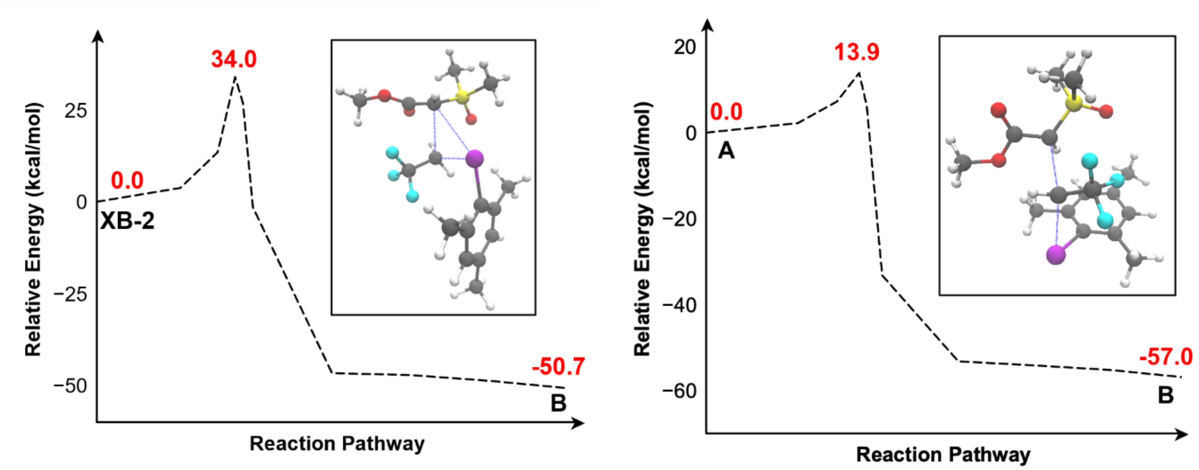

Figure S3. Path summary of the NEB-CI calculation for **Path 1** (left) and **Path 2** (left). Climbing image geometry shown (black outline).

Table S4. Path summary of the NEB-CI calculation for **Path 1**.

Image	Dist (Ang.)	E(Eh)	dE(kcal/mol)	Max(|Fp|)	RMS(Fp)	
0	0	-1841.95593	0	0.00018	0.00004	
1	4.341	-1841.94995	3.75	0.00057	0.00015	
2	6.283	-1841.93443	13.49	0.00197	0.00044	
3	7.188	-1841.90183	33.95	0.00035	0.00015	<=CI
4	7.596	-1841.91315	26.84	0.00088	0.00025	
5	8.114	-1841.95837	-1.53	0.00299	0.00055	
6	12.222	-1842.03028	-46.65	0.00285	0.00038	
7	16.329	-1842.03113	-47.19	0.00099	0.00019	
8	20.406	-1842.03361	-48.75	0.00169	0.00032	

Table S5. Path summary of the NEB-CI calculation for **Path 2**.

Image	Dist (Ang.)	E(Eh)	dE(kcal/mol)	Max(|Fp|)	RMS(Fp)	
0	0	-1841.94571	0	0.00587	0.00112	
1	3.851	-1841.9422	2.2	0.00021	0.00007	
2	5.499	-1841.93417	7.24	0.00025	0.0001	
3	6.433	-1841.92356	13.89	0.00027	0.00011	<=CI
4	6.754	-1841.9359	6.15	0.00029	0.00011	
5	7.4	-1841.99864	-33.21	0.00154	0.00028	
6	10.603	-1842.03054	-53.23	0.00096	0.00016	
7	13.818	-1842.03204	-54.18	0.00214	0.00025	
8	16.981	-1842.03372	-55.23	0.00325	0.0007	

4.3 Cartesian coordinates of fully optimized structures

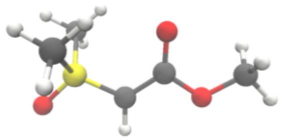

Label: 1a
Charge: 0
Multiplicity: 1
Final Gibbs free energy: -819.83030629 Eh
G-E(el): 0.11335911 Eh

C	-1.264983	-0.035537	0.000005
O	-2.416441	-0.750167	0.000002
O	-1.271981	1.195194	0.000000
C	-0.124304	-0.866151	0.000003
H	-0.143924	-1.945446	0.000003
S	1.401840	-0.216809	0.000000
O	2.424612	-1.251513	-0.000002
C	1.629033	0.866190	1.374181
H	0.811445	1.587571	1.370345
H	1.592985	0.239297	2.265241
H	2.596842	1.357574	1.269664
C	1.629028	0.866191	-1.374181
H	2.596837	1.357575	-1.269667
H	1.592977	0.239298	-2.265240
H	0.811440	1.587572	-1.370342
C	-3.620392	0.008213	-0.000002
H	-3.688365	0.636898	-0.889631
H	-4.428778	-0.720550	-0.000005
H	-3.688371	0.636899	0.889625

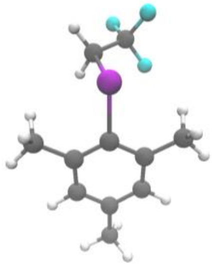

Label: n/a
Charge: 1
Multiplicity: 1
Final Gibbs free energy: -1023.37112150 Eh
G-E(el): 0.17310778 Eh

C	-2.788641	1.188613	-0.735226
C	-3.580942	0.631075	0.264979
C	-3.066008	-0.415024	1.021080
C	-1.782865	-0.916187	0.820901
C	-1.033977	-0.290950	-0.177402
C	-1.496431	0.747633	-0.991141
H	-3.185607	1.994121	-1.344660
H	-3.679188	-0.869131	1.792398
C	-1.296624	-2.063189	1.649158
H	-1.020707	-2.916116	1.023198
H	-0.420916	-1.793537	2.245450
H	-2.080030	-2.385063	2.334305
C	-0.702848	1.368180	-2.097473
H	-1.282719	2.158903	-2.572147
H	0.229673	1.803688	-1.731751
H	-0.446127	0.631471	-2.863559
C	-4.960044	1.153239	0.514496
H	-5.494627	0.536408	1.237055
H	-4.916208	2.175138	0.902395
H	-5.536800	1.187125	-0.412922
I	0.898379	-1.023857	-0.544788
C	2.003623	-0.115759	1.044184
H	1.283405	0.091334	1.833942
H	2.718493	-0.879966	1.346953
C	2.724885	1.140981	0.621672
F	3.468564	1.568740	1.646633
F	1.889184	2.125891	0.289025
F	3.540601	0.933638	-0.414654

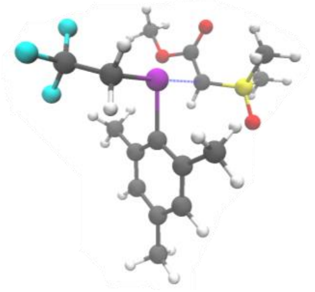

Label: XB-1
Charge: 1
Multiplicity: 1
Final Gibbs free energy: -1843.18525658 Eh
G-E(el): 0.31013005 Eh

C	-1.113792	2.136626	2.321601
O	-2.382571	1.806002	2.613728
O	-0.781127	3.279899	2.046465
C	-0.239619	0.998284	2.426905
H	-0.655192	0.003202	2.305831
S	1.269571	1.074731	1.671724
O	1.968009	-0.193350	1.769964
C	2.204407	2.387483	2.369556
H	1.600392	3.293614	2.325307
H	2.427526	2.107052	3.398743
H	3.117038	2.479486	1.779384
C	1.120145	1.545976	-0.022771
H	2.125782	1.643369	-0.434029
H	0.564770	0.747514	-0.515520
H	0.577172	2.490323	-0.069892
C	-3.331867	2.872054	2.594156

H	-3.383806	3.323844	1.602837
H	-4.286837	2.418351	2.849401
H	-3.070910	3.635225	3.328425
C	-0.816147	-3.332147	4.659573
C	0.441280	-3.924686	4.728749
C	1.544355	-3.118685	4.979694
C	1.433602	-1.742397	5.162401
C	0.144440	-1.215333	5.090763
C	-1.005796	-1.966153	4.835782
H	-1.687159	-3.949097	4.462407
H	2.531532	-3.565884	5.034071
C	2.663439	-0.928350	5.414016
H	2.784089	-0.149229	4.656986
H	2.634390	-0.438251	6.390959
H	3.545467	-1.567464	5.388085
C	-2.384635	-1.391861	4.742936
H	-3.099061	-2.177637	4.499380
H	-2.692232	-0.935326	5.686519
H	-2.451035	-0.621613	3.970203
C	0.596426	-5.398184	4.519390
H	1.605505	-5.730395	4.764587
H	-0.115180	-5.956367	5.132266
H	0.396305	-5.659411	3.476036
I	-0.068267	0.860522	5.364335
C	0.045097	0.834101	7.530412
H	0.537809	-0.093739	7.814324
H	0.661152	1.695917	7.782631
C	-1.295004	0.948520	8.200229
F	-1.129211	1.069004	9.525028
F	-2.067047	-0.124491	7.995691
F	-1.982867	2.020458	7.787440

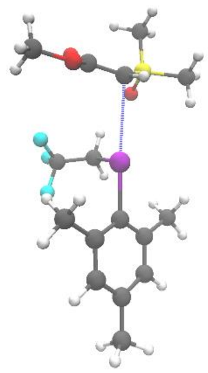

Label: XB-2
Charge: 1
Multiplicity: 1
Final Gibbs free energy: -1843.18700108 Eh
G-E(el): 0.30834917 Eh

C	-3.261356	0.839389	-0.724523
O	-2.881781	1.555573	-1.800446
O	-3.878520	1.336818	0.207989
C	-2.840770	-0.522762	-0.816066
H	-2.504839	-0.964989	-1.743459
S	-3.429450	-1.597592	0.312947
O	-2.909133	-1.472493	1.670305
C	-3.068381	-3.179775	-0.366859
H	-1.986581	-3.240916	-0.486026
H	-3.420497	-3.916277	0.355628
H	-3.580214	-3.285541	-1.322820
C	-5.194551	-1.568629	0.418997
H	-5.491213	-2.308140	1.163278
H	-5.469481	-0.562781	0.733382
H	-5.603906	-1.799606	-0.564225
C	-3.240869	2.935456	-1.791635
H	-4.324410	3.053874	-1.746486
H	-2.785447	3.449808	-0.944158
H	-2.859576	3.344571	-2.724822
C	4.507233	-1.388680	0.340089
C	5.219540	-0.331850	-0.215686
C	4.513989	0.707339	-0.813803
C	3.124308	0.734101	-0.854604
C	2.466665	-0.341686	-0.251494
C	3.116656	-1.431468	0.333217
H	5.045376	-2.217098	0.789542
H	5.057961	1.526664	-1.272870
C	2.427311	1.864464	-1.546411
H	1.858104	1.510059	-2.410347
H	1.730330	2.382828	-0.884494
H	3.158224	2.590186	-1.901712
C	2.410766	-2.610993	0.925538
H	1.819321	-2.333627	1.802233
H	1.733298	-3.075454	0.204353
H	3.136278	-3.360153	1.241061
C	6.714280	-0.305748	-0.162413
H	7.050842	0.269044	0.706421
H	7.125608	-1.311967	-0.071717
H	7.133336	0.171036	-1.050156
I	0.359593	-0.372170	-0.342051
C	-0.101857	0.216177	1.657299
H	-1.137351	-0.105248	1.786766
H	0.567407	-0.346137	2.304705
C	0.045071	1.697154	1.901869
F	1.306638	2.114560	1.761701
F	-0.328315	1.959612	3.159317
F	-0.715936	2.434068	1.090646

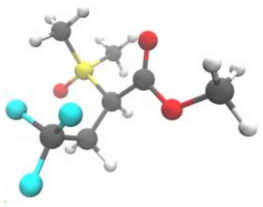

Label: B (path 1)
Charge: 1
Multiplicity: 1
Final Gibbs free energy: -1196.39679629 Eh
G-E(el): 0.15474881 Eh

C	-0.230485	1.590927	0.029805
O	-1.334297	2.018735	0.592099
O	0.331396	2.098841	-0.905384

C	0.294781	0.371674	0.780938
H	0.671965	0.750301	1.741281
S	1.840148	-0.285582	0.087248
O	2.157632	-1.519010	0.763767
C	3.024573	0.971340	0.373479
H	3.084897	1.111126	1.453818
H	3.965627	0.592167	-0.029913
H	2.706841	1.879123	-0.139601
C	1.765761	-0.477390	-1.653636
H	1.526063	0.484500	-2.103363
H	2.766812	-0.816559	-1.931575
H	1.020763	-1.237338	-1.880555
C	-1.936202	3.191576	0.012809
H	-2.209372	2.990162	-1.022494
H	-2.819160	3.386735	0.614182
H	-1.239731	4.027565	0.063607
C	-0.717044	-0.710491	1.146126
H	-0.227479	-1.499986	1.718295
H	-1.450024	-0.239599	1.803453
C	-1.482958	-1.368417	0.024131
F	-2.476850	-2.108263	0.520431
F	-0.709413	-2.189250	-0.706784
F	-2.023492	-0.487719	-0.828664

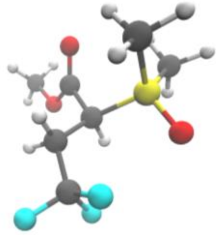

Label: B (path 2)
Charge: 1
Multiplicity: 1
Final Gibbs free energy: -1196.39687119 Eh
G-E(el): 0.15467507 Eh

C	0.799140	-0.129775	1.223808
H	0.285401	-0.763792	0.497155
H	1.707234	0.239380	0.743767
C	-0.122447	1.047707	1.460815
F	0.491589	2.094440	2.021449
F	-1.165636	0.730710	2.237500
F	-0.612335	1.470035	0.287865
C	1.711994	-2.337533	1.909402
O	0.760809	-3.130664	1.479659
O	2.893169	-2.565785	1.882821
C	1.101061	-1.033719	2.421553
H	0.180222	-1.261258	2.970326
S	2.122192	-0.292634	3.718841
O	1.397829	0.771846	4.365586
C	3.640717	0.235912	3.027339
H	4.104915	-0.610690	2.521740
H	3.420576	1.055572	2.342452
H	4.238447	0.585761	3.871755
C	2.495173	-1.594736	4.829023
H	3.022741	-1.116754	5.657328
H	1.542033	-2.009217	5.162403
H	3.119507	-2.337491	4.335421
C	1.182708	-4.375143	0.889435
H	1.716136	-4.970228	1.629635
H	0.267033	-4.871279	0.581813
H	1.823994	-4.177164	0.031312

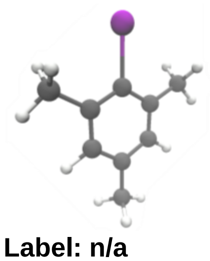

Label: n/a
Charge: 0
Multiplicity: 1
Final Gibbs free energy: -646.88346469 Eh
G-E(el): 0.13609029 Eh

C	2.231482	-1.189523	-0.004321
C	2.945536	0.004263	-0.003980
C	2.230243	1.194078	-0.004211
C	0.835828	1.225325	-0.001496
C	0.166002	0.001401	0.000511
C	0.840071	-1.223423	-0.001673
H	2.769809	-2.132944	-0.008211
H	2.766825	2.138220	-0.007946
C	0.129645	2.544409	-0.002658
H	-0.512346	2.651948	0.876194
H	0.851180	3.361975	-0.004165
H	-0.513776	2.649855	-0.880730
C	0.134594	-2.542892	-0.003146
H	-0.508685	-2.648476	-0.881284
H	0.856740	-3.359913	-0.004771
H	-0.507433	-2.650922	0.875597
C	4.444219	-0.000157	0.008387
H	4.824605	-0.358253	0.969860
H	4.842857	-0.664635	-0.762293
H	4.843914	1.001032	-0.158978
I	-1.938611	-0.001367	0.002515

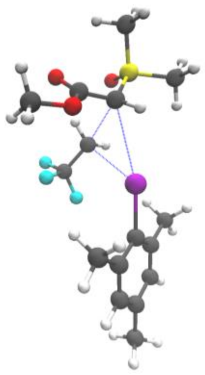

Label: TS_{path1}
Charge: 1
Multiplicity: 1
Final Gibbs free energy: -1843.12676877 Eh
G-E(el): 0.30640801 Eh
Imaginary frequency: -320.72 cm^{**-1}

C	-3.358488	1.051081	-0.455265
O	-2.860578	1.607678	-1.557116
O	-4.077632	1.632726	0.339129
C	-2.891361	-0.306131	-0.274138
H	-2.459192	-0.832834	-1.116933
S	-3.866759	-1.327228	0.692954
O	-3.786464	-1.097624	2.121015
C	-3.352378	-2.946291	0.256564
H	-2.298260	-3.032262	0.523023
H	-3.963594	-3.628900	0.848234
H	-3.509426	-3.094734	-0.812048
C	-5.545284	-1.225640	0.168437
H	-6.102000	-1.949625	0.766147
H	-5.877375	-0.208184	0.371477
F	-0.335771	2.514662	0.875646
H	-5.594457	-1.459170	-0.895348
C	-3.194988	2.982371	-1.775096
H	-4.274538	3.099896	-1.872107
H	-2.830306	3.597044	-0.951605
H	-2.699707	3.259053	-2.702292
C	4.473255	-1.065851	0.462558
C	5.117798	-0.064025	-0.256954
C	4.349925	0.781731	-1.050605
C	2.966934	0.663728	-1.138124
C	2.368144	-0.361640	-0.396308
C	3.094781	-1.238498	0.418808
H	5.059294	-1.738222	1.081450
H	4.839417	1.562656	-1.624225
C	2.198413	1.608090	-2.008265
H	1.651888	1.074142	-2.790317
H	1.463441	2.174495	-1.430119
H	2.875520	2.315625	-2.486677
C	2.459545	-2.328400	1.223479
H	1.739263	-1.924924	1.940584
H	1.919142	-3.032147	0.584480
H	3.219011	-2.881179	1.776203
C	6.599791	0.115984	-0.156143
H	7.003406	0.592764	-1.050701
H	6.847978	0.754781	0.697901
H	7.105485	-0.839374	-0.004991
I	0.291897	-0.580885	-0.493422
C	-1.293158	0.460185	1.581146
H	-2.300511	0.841402	1.657556
H	-1.089364	-0.460081	2.113122
C	-0.316942	1.578586	1.823629
F	0.944088	1.191578	2.002379
F	-0.709904	2.163591	2.972867

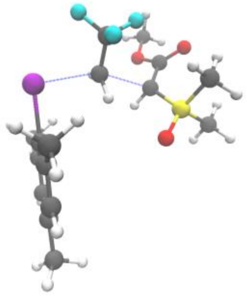

Label: TS$_{path2}$
Charge: 1
Multiplicity: 1
Final Gibbs free energy: -1843.16396726 Eh
G-E(el): 0.30613941 Eh
Imaginary frequency: -331.95 cm^{-1}**

C	-3.039454	2.482935	-1.105484
C	-4.012050	1.665853	-0.541040
C	-3.919621	0.291350	-0.734787
C	-2.887568	-0.287246	-1.465949
C	-1.937652	0.584184	-2.005992
C	-1.979957	1.971634	-1.849439
H	-3.101075	3.558015	-0.969071
H	-4.674686	-0.360039	-0.305930
C	-2.840295	-1.773185	-1.635895
H	-2.872325	-2.055562	-2.691551
H	-1.922688	-2.193493	-1.214478
H	-3.688647	-2.235804	-1.132023
C	-0.964133	2.904023	-2.430441
H	-1.209978	3.934441	-2.174773
H	0.038417	2.688099	-2.050807
H	-0.923530	2.821136	-3.519874
C	-5.121719	2.248286	0.277338
H	-4.870258	2.208905	1.342099
H	-5.298811	3.293130	0.018482
H	-6.048894	1.688459	0.141687
I	-0.367833	-0.233909	-3.126317
C	1.118355	-0.578090	-1.137031
H	0.617109	0.217863	-0.611707
H	0.821837	-1.606940	-1.014884
C	2.466540	-0.300195	-1.775002
F	3.497865	-0.700440	-1.031741
F	2.626750	1.005104	-2.006687
F	2.587511	-0.939611	-2.950158
C	2.699805	-2.024687	1.378831
O	2.309627	-3.215257	0.905109
O	3.806571	-1.830224	1.848665
C	1.645308	-1.044926	1.216536
H	0.624339	-1.413832	1.252221
S	1.711378	0.404109	2.098289
O	0.581100	1.249555	1.760816
C	3.244821	1.202973	1.804422
H	4.043972	0.489247	2.002268
H	3.244605	1.526420	0.764306
H	3.283338	2.057732	2.480716
C	1.731505	0.087570	3.833301
H	1.801242	1.047283	4.347334
H	0.794670	-0.420094	4.065257
H	2.589023	-0.548100	4.057373
C	3.277534	-4.264253	0.965549
H	3.577169	-4.451197	1.997329
H	2.784914	-5.141160	0.552207
H	4.155850	-4.010044	0.370917

5. X-ray crystallography data for compound 3f.

Table S6. Crystal data and structure refinement for compound **3f (CCDC2353834)**

Empirical formula	$C_{10}H_{17}F_3O_3S$
Formula weight	274.29
Temperature	296(2) K
Wavelength	0.71073 Å
Crystal system	Triclinic
Space group	*P*-1
Unit cell dimensions	*a* = 9.328(3) Å, *b* = 11.030(3) Å, *c* = 13.370(4) Å
	α = 84.945(8)°, β = 79.939(5)°, γ = 89.426(7)°.
Volume	1349.1(6) $Å^3$
Z	4
Density (calculated)	1.350 g/cm^3
Absorption coefficient	0.270 mm^{-1}
F(000)	576
Crystal size	0.130 x 0.120 x 0.020 mm^3
Theta range for data collection	2.312 to 25.998°.
Index ranges	-11<=h<=11, -13<=k<=13, -16<=l<=16
Reflections collected	23208
Independent reflections	5321 [R(int) = 0.0691]
Completeness to theta = 25.242°	99.9 %
Absorption correction	Semi-empirical from equivalents
Max. and min. transmission	0.7460 and 0.6743
Refinement method	Full-matrix least-squares on F^2
Data / restraints / parameters	5321 / 0 / 318
Goodness-of-fit on F^2	1.176
Final R indices [I>2sigma(I)]	R1 = 0.0594, wR2 = 0.1123
R indices (all data)	R1 = 0.1020, wR2 = 0.1258
Largest diff. peak and hole	0.655 and -0.318 e.$Å^{-3}$

Table S7. Atomic coordinates (x 10^4) and equivalent isotropic displacement parameters (Å^{2}x 10^3) for $C_{10}H_{17}F_3O_3S$

	x	y	z	U(eq)
C(1A)	185(4)	1377(3)	6972(3)	46(1)
C(2A)	-1043(4)	780(4)	7736(3)	71(1)
C(3A)	55(5)	2737(4)	6794(3)	76(1)
C(4A)	382(5)	819(4)	5968(3)	74(1)
O(5A)	1586(2)	1069(2)	7321(2)	49(1)
C(6A)	1973(4)	1532(3)	8136(3)	41(1)
O(7A)	1108(2)	2066(2)	8762(2)	53(1)
C(8A)	3456(3)	1298(3)	8190(2)	40(1)
S(9A)	4100(1)	1856(1)	9156(1)	38(1)
O(10A)	5631(2)	1559(2)	9130(2)	55(1)
C(11A)	3852(4)	3445(3)	9150(3)	58(1)
C(12A)	3086(4)	1337(3)	10347(3)	53(1)
C(13A)	4496(4)	679(3)	7415(3)	49(1)
C(14A)	5185(5)	1478(5)	6519(3)	69(1)
F(15A)	5949(3)	2392(3)	6753(2)	109(1)
F(16A)	6156(3)	871(3)	5882(2)	124(1)
F(17A)	4271(3)	1997(3)	5969(2)	124(1)
C(1B)	3650(4)	3779(3)	3116(3)	48(1)
C(2B)	3362(5)	4365(4)	4115(3)	72(1)
C(3B)	3389(5)	2423(4)	3299(3)	72(1)
C(4B)	2801(4)	4396(4)	2354(3)	71(1)
O(5B)	5221(2)	4028(2)	2769(2)	47(1)
C(6B)	5996(4)	3538(3)	1948(3)	40(1)
O(7B)	5445(2)	3058(2)	1306(2)	54(1)
C(8B)	7520(3)	3695(3)	1908(2)	37(1)
S(9B)	8638(1)	3151(1)	926(1)	36(1)
O(10B)	10158(2)	3400(2)	962(2)	51(1)
C(11B)	8379(4)	1572(3)	889(3)	56(1)
C(12B)	8228(4)	3736(3)	-258(3)	54(1)
C(13B)	8190(4)	4212(3)	2718(3)	47(1)
C(14B)	8427(5)	3330(5)	3567(3)	75(1)

F(15B)	9304(3)	2415(3)	3262(2)	104(1)
F(16B)	7230(3)	2822(4)	4093(2)	135(1)
F(17B)	9086(3)	3843(4)	4243(2)	128(1)
H(2AA)	-1950	941	7503	107
H(2AB)	-1074	1101	8383	107
H(2AC)	-888	-83	7806	107
H(3AA)	-751	2928	6450	114
H(3AB)	935	3064	6381	114
H(3AC)	-102	3086	7437	114
H(4AA)	-465	974	5659	112
H(4AB)	513	-44	6081	112
H(4AC)	1222	1171	5523	112
H(11A)	2832	3627	9219	87
H(11B)	4358	3837	8520	87
H(11C)	4230	3736	9709	87
H(12A)	2086	1570	10373	80
H(12B)	3469	1692	10877	80
H(12C)	3147	467	10444	80
H(13A)	5257	305	7745	59
H(13B)	3978	32	7180	59
H(2BA)	2355	4259	4418	108
H(2BB)	3588	5218	3990	108
H(2BC)	3960	3988	4569	108
H(3BA)	2398	2268	3622	108
H(3BB)	4032	2072	3732	108
H(3BC)	3571	2067	2660	108
H(4BA)	1778	4328	2622	106
H(4BB)	3011	4011	1728	106
H(4BC)	3074	5239	2227	106
H(11D)	7389	1418	826	83
H(11E)	8584	1147	1506	83
H(11F)	9023	1293	315	83
H(12D)	7224	3576	-278	80
H(12E)	8835	3353	-796	80
H(12F)	8402	4598	-348	80
H(13C)	9120	4578	2404	57

H(13D)	7568	4855	2995	57

U(eq) is defined as one third of the trace of the orthogonalized U^{ij} tensor.

Table S8. Anisotropic displacement parameters (Å^{2}x 10^3) for $C_{10}H_{17}F_3O_3S$

	U^{11}	U^{22}	U^{33}	U^{23}	U^{13}	U^{12}
C(1A)	44(2)	56(2)	45(2)	-11(2)	-19(2)	1(2)
C(2A)	46(2)	97(3)	72(3)	3(3)	-20(2)	-16(2)
C(3A)	106(4)	63(3)	66(3)	-3(2)	-35(3)	5(3)
C(4A)	87(3)	92(3)	56(3)	-25(2)	-35(2)	8(3)
O(5A)	38(1)	67(2)	48(2)	-24(1)	-12(1)	4(1)
C(6A)	40(2)	48(2)	37(2)	-9(2)	-7(2)	-1(2)
O(7A)	38(1)	78(2)	50(2)	-29(1)	-13(1)	17(1)
C(8A)	34(2)	43(2)	43(2)	-9(2)	-9(2)	3(2)
S(9A)	34(1)	36(1)	45(1)	-5(1)	-11(1)	2(1)
O(10A)	32(1)	68(2)	69(2)	-16(1)	-17(1)	10(1)
C(11A)	69(3)	38(2)	73(3)	-4(2)	-27(2)	-3(2)
C(12A)	54(2)	57(2)	49(2)	-1(2)	-14(2)	-2(2)
C(13A)	44(2)	56(2)	47(2)	-8(2)	-7(2)	8(2)
C(14A)	47(2)	93(4)	62(3)	4(3)	-4(2)	-1(2)
F(15A)	96(2)	108(2)	111(2)	29(2)	-3(2)	-35(2)
F(16A)	100(2)	173(3)	78(2)	-18(2)	41(2)	20(2)
F(17A)	84(2)	192(3)	85(2)	63(2)	-17(2)	3(2)
C(1B)	32(2)	61(2)	46(2)	-3(2)	1(2)	2(2)
C(2B)	64(3)	90(3)	56(3)	-15(2)	8(2)	13(2)
C(3B)	77(3)	69(3)	65(3)	2(2)	2(2)	-5(2)
C(4B)	38(2)	91(3)	80(3)	8(3)	-11(2)	11(2)
O(5B)	33(1)	64(2)	45(2)	-17(1)	-3(1)	6(1)
C(6B)	39(2)	43(2)	39(2)	-4(2)	-7(2)	1(2)
O(7B)	38(1)	79(2)	48(2)	-24(1)	-9(1)	-5(1)
C(8B)	29(2)	41(2)	42(2)	-6(2)	-7(1)	2(1)
S(9B)	32(1)	36(1)	41(1)	-4(1)	-6(1)	-1(1)
O(10B)	30(1)	65(2)	60(2)	-15(1)	-3(1)	-4(1)
C(11B)	62(2)	37(2)	65(3)	-9(2)	-2(2)	3(2)
C(12B)	56(2)	59(2)	44(2)	2(2)	-6(2)	1(2)
C(13B)	38(2)	55(2)	51(2)	-20(2)	-8(2)	1(2)
C(14B)	57(3)	116(4)	56(3)	-10(3)	-22(2)	13(3)
F(15B)	118(2)	102(2)	94(2)	7(2)	-33(2)	41(2)

F(16B)	87(2)	207(4)	95(2)	75(2)	-14(2)	-20(2)
F(17B)	111(2)	221(4)	73(2)	-46(2)	-55(2)	20(2)

The anisotropic displacement factor exponent takes the form: $-2\pi^2[h^2 a^{*2}U^{11} + ... + 2 h k a^* b^* U^{12}]$

Table S9. Bond lengths [Å] and angles [°] for $C_{10}H_{17}F_3O_3S$

C(1A)-O(5A)	1.489(4)
C(1A)-C(3A)	1.505(5)
C(1A)-C(4A)	1.508(5)
C(1A)-C(2A)	1.509(5)
O(5A)-C(6A)	1.348(4)
C(6A)-O(7A)	1.241(4)
C(6A)-C(8A)	1.419(4)
C(8A)-C(13A)	1.497(4)
C(8A)-S(9A)	1.680(3)
S(9A)-O(10A)	1.457(2)
S(9A)-C(12A)	1.756(4)
S(9A)-C(11A)	1.766(3)
C(13A)-C(14A)	1.477(5)
C(14A)-F(17A)	1.315(5)
C(14A)-F(15A)	1.331(5)
C(14A)-F(16A)	1.346(5)
C(1B)-O(5B)	1.478(4)
C(1B)-C(4B)	1.509(5)
C(1B)-C(3B)	1.510(5)
C(1B)-C(2B)	1.515(5)
O(5B)-C(6B)	1.356(4)
C(6B)-O(7B)	1.232(4)
C(6B)-C(8B)	1.426(4)
C(8B)-C(13B)	1.497(4)
C(8B)-S(9B)	1.678(3)
S(9B)-O(10B)	1.457(2)
S(9B)-C(12B)	1.760(4)

S(9B)-C(11B)	1.766(3)
C(13B)-C(14B)	1.474(6)
C(14B)-F(16B)	1.313(5)
C(14B)-F(15B)	1.339(5)
C(14B)-F(17B)	1.344(5)
O(5A)-C(1A)-C(3A)	109.7(3)
O(5A)-C(1A)-C(4A)	101.9(3)
C(3A)-C(1A)-C(4A)	109.7(3)
O(5A)-C(1A)-C(2A)	108.7(3)
C(3A)-C(1A)-C(2A)	114.3(3)
C(4A)-C(1A)-C(2A)	111.8(3)
C(6A)-O(5A)-C(1A)	122.5(3)
O(7A)-C(6A)-O(5A)	123.0(3)
O(7A)-C(6A)-C(8A)	125.5(3)
O(5A)-C(6A)-C(8A)	111.5(3)
C(6A)-C(8A)-C(13A)	124.8(3)
C(6A)-C(8A)-S(9A)	116.6(2)
C(13A)-C(8A)-S(9A)	118.4(2)
O(10A)-S(9A)-C(8A)	111.51(15)
O(10A)-S(9A)-C(12A)	109.76(16)
C(8A)-S(9A)-C(12A)	111.92(17)
O(10A)-S(9A)-C(11A)	110.11(17)
C(8A)-S(9A)-C(11A)	111.46(17)
C(12A)-S(9A)-C(11A)	101.66(19)
C(14A)-C(13A)-C(8A)	115.2(3)
F(17A)-C(14A)-F(15A)	105.0(4)
F(17A)-C(14A)-F(16A)	106.6(4)
F(15A)-C(14A)-F(16A)	104.3(4)
F(17A)-C(14A)-C(13A)	114.7(4)
F(15A)-C(14A)-C(13A)	113.6(4)
F(16A)-C(14A)-C(13A)	111.7(4)
O(5B)-C(1B)-C(4B)	109.4(3)
O(5B)-C(1B)-C(3B)	109.8(3)
C(4B)-C(1B)-C(3B)	113.4(3)
O(5B)-C(1B)-C(2B)	101.8(3)

C(4B)-C(1B)-C(2B)	111.3(3)
C(3B)-C(1B)-C(2B)	110.5(3)
C(6B)-O(5B)-C(1B)	122.3(3)
O(7B)-C(6B)-O(5B)	124.1(3)
O(7B)-C(6B)-C(8B)	124.8(3)
O(5B)-C(6B)-C(8B)	111.0(3)
C(6B)-C(8B)-C(13B)	124.7(3)
C(6B)-C(8B)-S(9B)	117.1(2)
C(13B)-C(8B)-S(9B)	118.0(2)
O(10B)-S(9B)-C(8B)	111.49(15)
O(10B)-S(9B)-C(12B)	109.65(16)
C(8B)-S(9B)-C(12B)	112.05(17)
O(10B)-S(9B)-C(11B)	109.81(16)
C(8B)-S(9B)-C(11B)	111.61(17)
C(12B)-S(9B)-C(11B)	101.81(19)
C(14B)-C(13B)-C(8B)	115.2(3)
F(16B)-C(14B)-F(15B)	106.2(5)
F(16B)-C(14B)-F(17B)	106.1(4)
F(15B)-C(14B)-F(17B)	104.6(4)
F(16B)-C(14B)-C(13B)	114.3(4)
F(15B)-C(14B)-C(13B)	112.9(4)
F(17B)-C(14B)-C(13B)	112.0(4)

Symmetry transformations used to generate equivalent atoms:

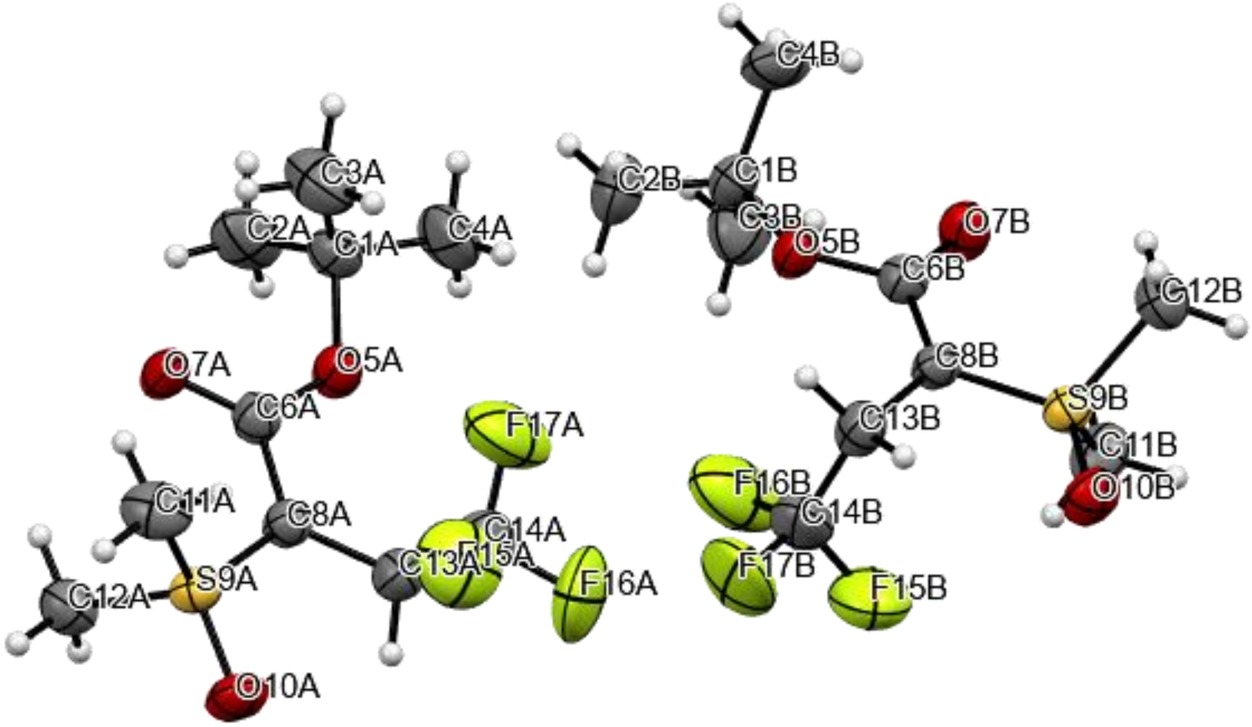

Figure S4: ORTEP type representation of the asymmetric unit of **3f** with labeling scheme.

References

(1) Pracht, P.; Bohle, F.; Grimme, S. Automated exploration of the low-energy chemical space with fast quantum chemical methods. *Phys. Chem. Chem. Phys.* **2020**, *22* (14), 7169-7192.

(2) *Gaussian 16 Rev. C.01*; Wallingford, CT, 2016.

(3) Perdew, J. P.; Burke, K.; Ernzerhof, M. Generalized Gradient Approximation Made Simple. *Phys. Rev. Lett.* **1996**, *77* (18), 3865-3868.

(4) Adamo, C.; Barone, V. Toward reliable density functional methods without adjustable parameters: The PBE0 model. *J. Chem. Phys.* **1999**, *110* (13), 6158-6170.

(5) Weigend, F.; Ahlrichs, R. Balanced basis sets of split valence, triple zeta valence and quadruple zeta valence quality for H to Rn: Design and assessment of accuracy. *Phys. Chem. Chem. Phys.* **2005**, *7* (18), 3297-3305.

(6) Rappoport, D.; Furche, F. Property-optimized Gaussian basis sets for molecular response calculations. *J. Chem. Phys.* **2010**, *133* (13).

(7) Peterson, K. A.; Figgen, D.; Goll, E.; Stoll, H.; Dolg, M. Systematically convergent basis sets with relativistic pseudopotentials. II. Small-core pseudopotentials and correlation consistent basis sets for the post-d group 16–18 elements. *J. Chem. Phys.* **2003**, *119* (21), 11113-11123.

(8) Stoychev, G. L.; Auer, A. A.; Neese, F. Automatic Generation of Auxiliary Basis Sets. *J. Chem. Theory Comput.* **2017**, *13* (2), 554-562.

(9) Neese, F. The ORCA program system. *WIREs Computational Molecular Science* **2012**, *2* (1), 73-78.

(10) Neese, F. Software update: The ORCA program system—Version 5.0. *WIREs Computational Molecular Science* **2022**, *12* (5), e1606.

(11) Fukui, K. The path of chemical reactions - the IRC approach. *Acc. Chem. Res.* **1981**, *14* (12), 363-368.

(12) Marenich, A. V.; Cramer, C. J.; Truhlar, D. G. Universal Solvation Model Based on Solute Electron Density and on a Continuum Model of the Solvent Defined by the Bulk Dielectric Constant and Atomic Surface Tensions. *J. Chem. Phys. B* **2009**, *113* (18), 6378-6396.

(13) *GaussView Version6*; 2019.

(14) Hanwell, M. D.; Curtis, D. E.; Lonie, D. C.; Vandermeersch, T.; Zurek, E.; Hutchison, G. R. Avogadro: an advanced semantic chemical editor, visualization, and analysis platform. *J. Cheminform.* **2012**, *4* (1), 17.

(15) Humphrey, W.; Dalke, A.; Schulten, K. VMD: visual molecular dynamics. *J. Mol. Graph.* **1996**, *14* (1), 33-38, 27-38.

(16) Bader, R. F. W.; Carroll, M. T.; Cheeseman, J. R.; Chang, C. Properties of atoms in molecules: atomic volumes. *J. Am. Chem. Soc.* **1987**, *109* (26), 7968-7979.

(17) Caldeweyher, E.; Bannwarth, C.; Grimme, S. Extension of the D3 dispersion coefficient model. *J. Chem. Phys.***2017**, *147* (3).

(18) Ásgeirsson, V.; Birgisson, B. O.; Bjornsson, R.; Becker, U.; Neese, F.; Riplinger, C.; Jónsson, H. Nudged Elastic Band Method for Molecular Reactions Using Energy-Weighted Springs Combined with Eigenvector Following. *J. Chem. Theory Comput.* **2021**, *17* (8), 4929-4945.
